# Supplementary figures and images for: Child acute illness presentation and referrals at primary health clinics in Malawi: a secondary analysis of ASPIRE
Source: BMJ Open. 2024 Apr 25;14(4):e079589. doi: 10.1136/bmjopen-2023-079589 (PMC11057250; doi:10.1136/bmjopen-2023-079589)

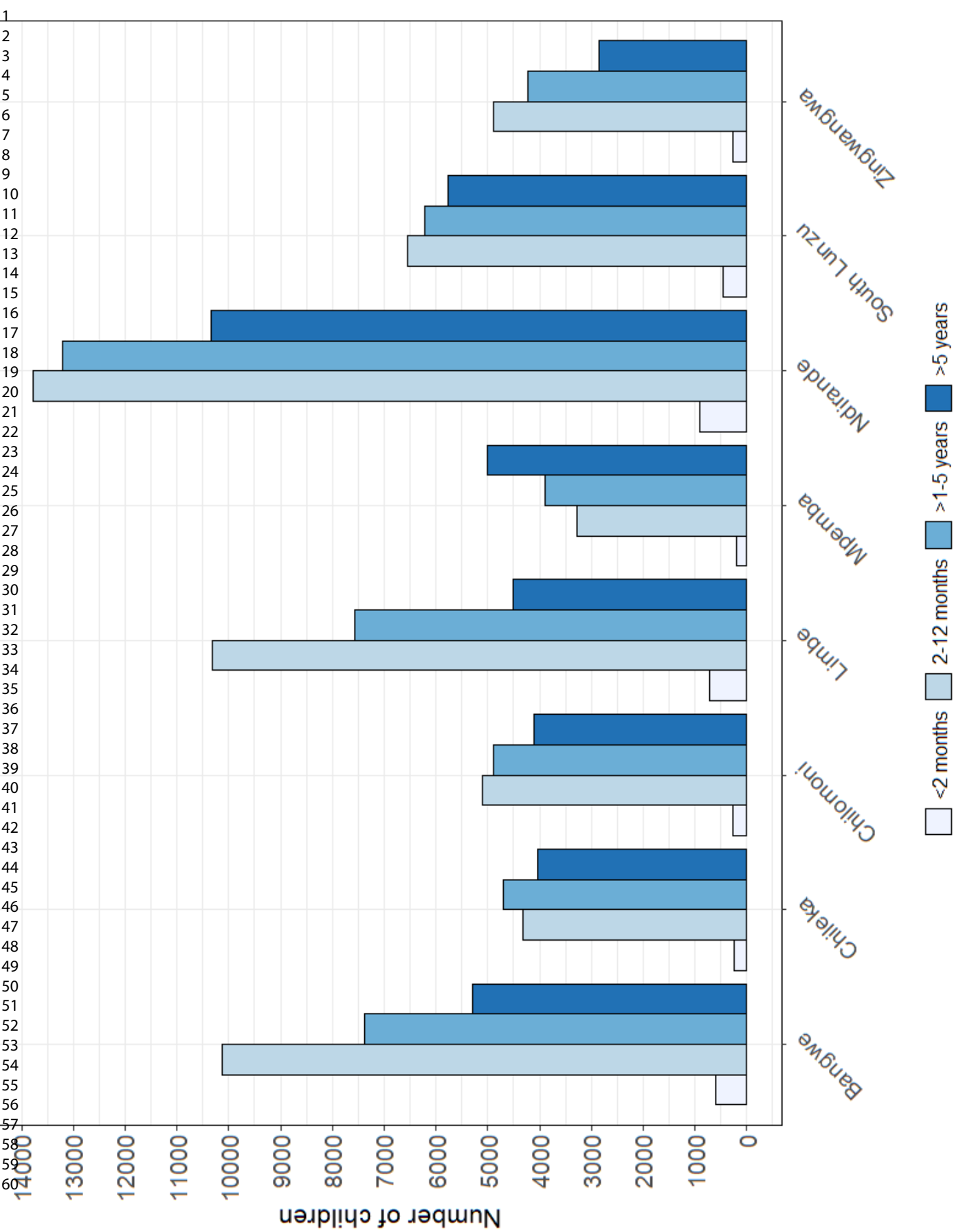

Supplement: Supplementary data [file bmjopen-2023-079589supp002.pdf]
