# Supplementary material for: Child acute illness presentation and referrals at primary health clinics in Malawi: a secondary analysis of ASPIRE
Source: BMJ Open. 2024 Apr 25;14(4):e079589. doi: 10.1136/bmjopen-2023-079589 (PMC11057250; doi:10.1136/bmjopen-2023-079589)
Supplement: Supplementary data [file bmjopen-2023-079589supp001.pdf]

Supplementary Table 1. Complete characteristics of children attending primary health centre

Characteristics	Total		<2 months		2-12 months		>1-5 years		>5 years	
	N=155,931	%	N=3,654	%	N=58,337	%	N=52,064	%	N=41,876	%
Male	77,915	50·0	1,889	51·7	29,885	51·2	26,204	50·3	19,937	47·6
Weight, kg (mean | SD)	15·34	8·33	4·09	1·22	8·48	2·39	13·74	3·42	24·59	7·18
**PHC**										
Bangwe	23,389	15·0	604	16·5	10,115	17·3	7,388	14·2	5,282	12·6
Chileka	13,266	8·5	236	6·5	4,309	7·4	4,694	9·0	4,027	9·6
Chilomoni	14,342	9·2	265	7·3	5,097	8·7	4,878	9·4	4,102	9·8
Limbe	23,099	14·8	713	19·5	10,313	17·7	7,564	14·5	4,509	10·8
Mpemba	12,388	7·9	207	5·7	3,277	5·6	3,904	7·5	5,000	11·9
Ndirande	38,243	24·5	912	25·0	13,799	23·6	13,210	25·4	10,342	24·7
South Lunzu	18,990	12·2	457	12·5	6,557	11·2	6,207	11·9	5,769	13·8
Zingwangwa	12,214	7·8	260	7·1	4,890	8·4	4,219	8·1	2,845	6·8
**PHC mHealth triage**										
Emergency	392	0·3	19	0·5	162	0·3	136	0·3	75	0·2
Priority	48,248	30·9	2,974	81·4	15,694	26·9	16,912	32·5	12,668	30·3
Queue	107,291	68·8	661	18·1	42,481	72·8	35,016	67·3	29,133	69·6
**PHC clinician triage**										
Emergency	186	0·1	7	0·2	78	0·1	57	0·1	44	0·1
Priority	33,633	21·6	2,005	54·9	11,370	19·5	11,564	22·2	8,694	20·8
Queue	122,112	78·3	1,642	44·9	46,889	80·4	40,443	77·7	33,138	79·1
**PHC symptoms**										
Fever	515	0·3	23	0·7	231	0·4	166	0·3	93	0·2
Convulsions	100	0·1	1	<0·1	26	<0·1	46	0·1	27	0·1
Diarrhoea	149	0·1	1	<0·1	86	0·1	37	0·1	25	0·1
Vomiting	271	0·2	9	0·2	127	0·2	85	0·2	50	0·1
Cough	340	0·2	24	0·7	201	0·3	74	0·1	41	0·1
Difficulty breathing	309	0·2	26	0·7	199	0·3	65	0·1	19	<0·1
Rash	102	0·1	5	0·1	26	<0·1	35	0·1	36	0·1
Pallor	37	<0·1	1	<0·1	10	<0·1	19	<0·1	7	<0·1
Jaundice	13	<0·1	2	0·1	3	<0·1	5	<0·1	3	<0·1
Oedema	27	<0·1	0	0	10	<0·1	11	<0·1	6	<0·1
Urination problems	13	<0·1	3	0·1	1	<0·1	6	<0·1	3	<0·1
Feeding problems	97	0·1	5	0·1	46	0·1	27	0·1	19	<0·1
Other	1,498	1·0	50	1·4	227	0·4	557	1·1	664	1·6
**PHC laboratory test**	503	0·3	10	0·3	203	0·3	168	0·3	122	0·3
Haemoglobin	46	<0·1	1	<0·1	11	<0·1	20	<0·1	14	<0·1
Glucose	7	<0·1	0	0	1	<0·1	4	<0·1	2	<0·1
MPS	16	<0·1	0	0	3	<0·1	9	<0·1	4	<0·1
MRDT	483	0·3	9	0·2	201	0·3	159	0·3	114	0·3
Urine	4	<0·1	0	0	0	0	3	<0·1	1	<0·1
**PHC outcome**										
Admitted to short stay	181	0·1	3	0·1	67	0·1	62	0·1	49	0·1
Referred after triage	2,487	1·6	109	3·0	562	1·0	846	1·6	970	2·3
Referred after short stay	517[a]	0·3	28	0·8	128	0·2	188	0·4	173	0·4
Total referred from HC	3,004	1·9	137	3·7	690	1·2	1,034	2·0	1,143	2·7
Successful referral[b]	1,644/ 3,004	54·7	72/ 137	52·6	393/ 690	57·0	582/ 1,034	56·3	597/ 1,143	52·2
Self-referral[c]	372	0·2	21	0·6	132	0·2	130	0·2	89	0·2
**QECH A&E**										
Successful referral arrived by ambulance	53/ 1,644	3·2	2/ 137	1·5	24/ 690	3·5	16/ 1,034	1·5	11/ 1,143	1·0
Self-referral arrived by ambulance	22/ 372	5·9	3/ 21	14·3	9/ 132	6·8	5/ 130	3·8	5/ 89	5·6
Total arrived by ambulance	75/ 2,016	3·7	5/ 93	5·4	33/ 525	6·3	21/ 712	4·4	16/ 686	2·3
**QECH symptoms**										

Fever	205/ 2,016	10·2	20/ 93	21·5	111/ 525	21·1	39/ 712	5·5	35/ 686	5·1
Convulsions	32/ 2,016	1·6	1/ 93	1·1	7/ 525	1·3	20/ 712	2·8	4/ 686	0·6
Diarrhoea	49/ 2,016	2·4	2/ 93	2·2	39/ 525	7·4	4/ 712	0·6	4/ 686	0·6
Vomiting	75/ 2,016	3·7	5/ 93	5·4	41/ 525	7·8	13/ 712	1·8	16/ 686	2·3
Cough	159/ 2,016	7·9	15/ 93	16·1	94/ 525	17·9	28/ 712	3·9	22/ 686	2·8
Difficulty breathing	148/ 2,016	7·3	15/ 93	16·1	89/ 525	17·0	25/ 712	3·5	19/ 686	3·2
Rash	7/ 2,016	0·3	0	0	4/ 525	0·8	3/ 712	0·4	0	0
Pallor	25/ 2,016	1·2	0	0	6/ 525	1·1	8/ 712	1·1	11/ 686	1·6
Jaundice	11/ 2,016	0·5	7/ 93	7·5	0	0	3/ 712	0·4	1/ 686	0·1
Oedema	16/ 2,016	0·8	0	0	7/ 525	1·3	3/ 712	0·4	6/ 686	0·9
Urination problems	3/ 2,016	0·1	1/ 93	1·1	0	0	1/ 712	0·1	1/ 686	0·1
Feeding problems	52/ 2,016	2·6	8/ 93	8·6	27/ 525	5·1	6/ 712	0·8	11/ 686	1·6
Other	117/ 2,016	5·8	10/ 93	10·8	42/ 525	8·0	26/ 712	3·7	39/ 686	5·7
**QECH diagnosis**										
Trauma	9/ 2,016	0·4	0	0	2/ 525	0·4	4/ 712	0·6	3/ 686	0·4
Gastroenteritis	35/ 2,016	1·7	2/ 93	2·2	29/ 525	5·5	2/ 712	0·3	2/ 686	0·3
Pneumonia	52/ 2,016	2·6	4/ 93	4·3	37/ 525	7·0	5/ 712	0·7	6/ 686	0·9
Meningitis	6/ 2,016	0·3	0	0	2/ 525	0·4	3/ 712	0·4	1/ 686	0·1
Malnutrition	25/ 2,016	1·2	1/ 93	1·1	16/ 525	3·0	4/ 712	0·6	4/ 686	0·6
Sepsis	16/ 2,016	0·8	13/ 93	14·0	3/ 525	0·6	0	0	0	0
Malaria	41/ 2,016	2·0	0	0	14/ 525	2·7	18/ 712	2·5	9/ 686	1·3
Anaemia	26/ 2,016	1·3	1/ 93	1·1	8/ 525	1·5	7/ 712	0·9	10/ 686	1·5
Other	201/ 2,016	10·0	19/ 93	2-·4	97/ 525	18·5	41/ 712	5·8	44/ 686	6·4
**QECH laboratory test**	193/ 2,016	9·6	18/ 93	19·4	97/ 525	18·5	47/ 712	5·8	36/ 686	5·2
CSF culture	11/ 2,016	0·5	4/ 93	4·3	2/ 525	0·4	3/ 712	0·4	2/ 686	0·3
Blood culture	41/ 2,016	2·0	10/ 93	10·8	15/ 525	2·9	5/ 712	0·7	11/ 686	1·6
Blood glucose	23/ 2,016	1·1	2/ 93	2·2	15/ 525	2·9	3/ 712	0·4	3/ 686	0·4
HIV	148/ 2,016	7·3	12/ 93	12·9	74/ 525	14·1	32/ 712	4·5	30/ 686	4·4
MRDT	90/ 2,016	4·5	7/ 93	7·5	42/ 525	8·0	22/ 712	3·1	19/ 686	2·8
MPS	127/ 2,016	6·3	13/ 93	14·0	66/ 525	12·6	23/ 712	3·2	25/ 686	3·6
PCV	127/ 2,016	6·3	13/ 93	14·0	67/ 525	12·8	22/ 712	3·1	25/ 686	3·6
Other	38/ 2,016	1·9	1/ 93	1·1	13/ 525	2·5	8/ 712	1·1	16/ 686	2·3
**QECH laboratory test result**										
Positive blood culture	5/ 41	12·2	1/ 10	10	0/ 15	0	1/ 5	20	3/ 11	27
Positive CSF culture	11/ 11	100	4/ 4	100	2/ 2	100	3/ 3	100	2/2	100
Positive HIV test	8/ 148	5·4	0/ 12	0	2/ 74	27	0/ 32	0	6/ 30	20
Positive MRDT	17/ 90	18·9	0/ 7	0	5/ 42	12	7/ 22	32	5/ 19	26
Positive MPS	19/ 127	15·0	0/ 13	0	5/ 66	8	7/ 23	30	5/ 25	20
Low blood glucose	5/ 23	21·7	1/ 2	50	2/ 15	14	0/ 3	0	2/ 3	67
PCV (mean | SD)	32·17	8·11	35·62	7·59	32·76	6·11	33·68	8·12	27·44	11·12
**QECH A&E outcome**										
Died	2/ 2290	0·1	0/ 119	0	2/ 663	0·1	0/ 770	0	0/ 738	0

A&E, accident and emergency; CSF, cerebrospinal fluid; HIV, human immunodeficiency virus; mHealth, mobile health; MRDT, malaria rapid diagnostic test; MPS, malaria parasite screen; PCV, packed cell volume; PHC, primary health centre; QECH, Queen Elizabeth Central Hospital; SD, standard deviation

[a]517 cases that were referred to QECH from PHC were still admitted to short stay at the PHC, where they were again referred to QECH.

[b]Referred by PHC and arrived at QECH.

[c]Not referred by PHC but arrived at QECH.

Supplementary Table 2A. For young infants <2 months, emergency and priority signs, referral, and successful referral

	Referred		Not referred		Successfully referred		Not successfully referred	
	N=137	%	N=3,517	%	N=72	%	N=65	%
**Emergency signs**								
**Breathing**								
Obstructed breathing	1	0.7	8	0.2	0	0	1	1.5
Central cyanosis	0	0	1	<0.1	0	0	0	0
Respiratory distress	1	0.7	8	0.2	0	0	1	1.5
**Circulation**								
Cold hands	0	0	45	1.3	0	0	0	0
Capillary refill >3 secs	0	0	1	2.2	0	0	0	0
Weak and fast pulse	0	0	1	2.2	0	0	0	0
**Consciousness**								
Coma	0	0	0	0	0	0	0	0
Convulsions	1	0.7	0	0	0	0	1	1.5
**Dehydration**								
Diarrhoea	4	3.0	195	5.6	1	1.4	3	4.6
Lethargic or unconscious	0	0	3,306	94.0	0	0	0	0
Sunken eyes	0	0	0	0	0	0	0	0
Very slow skin pinch	0	0	0	0	0	0	0	0
**Priority signs**								
Tiny baby	108	78.2	2,616	74.4	60	83.3	49	74.2
Temperature	7	5.1	181	5.1	3	4.2	4	6.1
Trauma	2	1.5	10	0.3	1	1.4	1	1.5
Severe pain	0	0	28	0.8	0	0	0	0
Respiratory distress	0	0	21	0.6	0	0	0	0
Burns	0	0	1	<0.1	0	0	0	0

Supplementary Table 2B. For infants 2-12 months, emergency and priority signs, referral, and successful referral

	Referred		Not referred		Successfully referred		Not successfully referred	
	N=690	%	N=57,647	%	N=393	%	N=297	%
**Emergency signs**								
**Breathing**								
Obstructed breathing	23	3.3	49	<0.1	17	4.3	6	2.0
Central cyanosis	0	0	0	0	0	0	0	0

Respiratory distress	25	3.6	44	<0.1	16	4.1	9	3.0
**Circulation**								
Cold hands	3	0.4	621	1.1	1	0.3	2	0.7
Capillary refill >3 secs	0	0	5	<0.1	0	0	0	0
Weak and fast pulse	0	0	8	<0.1	0	0	0	0
**Consciousness**								
Coma	0	0	1	<0.1	0	0	0	0
Convulsions	7	1.1	9	<0.1	5	1.3	2	0.7
**Dehydration**								
Diarrhoea	89	12.9	13,828	24.0	62	15.8	27	9.1
Lethargic or unconscious	3	0.4	66	0.1	2	0.5	1	0.3
Sunken eyes	15	2.2	182	0.3	13	3.3	2	0.7
Very slow skin pinch	1	0.1	34	0.1	1	0.3	0	0
**Priority signs**								
Tiny baby	29	4.2	1,152	2.0	16	4.1	13	4.4
Temperature	133	19.3	12,383	21.5	80	20.4	53	17.8
Trauma	94	13.6	580	1.0	61	15.5	33	11.1
Severe pallor	1	0.1	32	<0.1	0	0	1	0.3
Poisoning	3	0.4	7	<0.1	3	0.8	0	0
Severe pain	33	4.8	391	0.7	16	4.1	17	5.7
Respiratory distress	31	4.5	533	0.9	22	5.6	9	3.0
Restless/lethargic	4	0.6	81	0.1	4	1.0	0	0
Urgent referral	4	0.6	8	<0.1	3	0.8	1	0.3
Malnutrition	7	1.0	26	<0.1	6	1.5	1	0.3
Oedema	2	0.3	18	<0.1	1	0.3	1	0.3
Burns	17	2.5	125	0.2	3	0.8	14	4.7

Supplementary Table 2C. For children >1-5 years, emergency and priority signs, referral, and successful referral

	**Referred**		**Not referred**		**Successfully referred**		**Not successfully referred**	
	N=1,034	%	N=51,030	%	N=582	%	N=452	%
**Emergency signs**								
**Breathing**								
Obstructed breathing	4	0.4	45	<0.1	2	0.3	2	0.4
Central cyanosis	1	<0.1	3	<0.1	0	0	1	0.2
Respiratory distress	9	0.9	30	<0.1	5	0.9	4	0.9
**Circulation**								
Cold hands	8	0.8	548	1.1	4	0.7	4	0.9
Capillary refill >3 secs	0	0	2	<0.1	0	0	0	0
Weak and fast pulse	1	0.1	11	<0.1	1	0.2	0	0
**Consciousness**								
Coma	3	0.3	2	<0.1	1	0.2	2	0.4
Convulsions	16	1.6	20	<0.1	10	1.7	6	1.3
**Dehydration**								

Diarrhoea	37	3.7	8,298	16.3	22	3.8	15	3.3
Lethargic or unconscious	3	0.3	52	0.1	0	0	3	0.7
Sunken eyes	1	0.1	98	0.2	0	0	1	0.2
Very slow skin pinch	0	0	26	0.1	0	0	0	0
**Priority signs**								
Tiny baby	0	0	88	0.2	0	0	0	0
Temperature	89	8.6	13,826	27.1	53	9.1	36	8.0
Trauma	401	38.8	1,029	2.0	242	41.6	159	35.2
Severe pallor	2	0.2	209	0.4	0	0	2	0.4
Poisoning	1	<0.1	12	<0.1	0	0	1	0.2
Severe pain	67	6.5	631	1.2	36	6.2	31	6.9
Respiratory distress	16	1.5	244	0.5	13	2.2	3	0.7
Restless/lethargic	1	<0.1	44	<0.1	0	0	1	0.2
Urgent referral	3	0.3	8	<0.1	0	0	3	0.7
Malnutrition	5	0.5	15	<0.1	0	0	5	1.1
Oedema	7	0.7	28	<0.1	2	0.3	5	1.1
Burns	38	3.7	148	0.3	16	2.7	22	4.9

Supplementary Table 2D. For children >5 years, emergency and priority signs, referral, and successful referral

	Referred		Not referred		Successfully referred		Not successfully referred	
	N=1,143	%	N=40,733	%	N=597	%	N=546	%
**Emergency signs**								
**Breathing**								
Obstructed breathing	1	<0.1	31	<0.1	0	0	1	0.2
Central cyanosis	0	0	0	0	0	0	0	0
Respiratory distress	1	<0.1	13	<0.1	1	0.2	0	0
**Circulation**								
Cold hands	2	0.2	367	0.9	1	0.2	1	0.2
Capillary refill >3 secs	0	0	4	1.1	0	0	0	0
Weak and fast pulse	1	<0.1	10	2.7	1	0.2	0	0
**Consciousness**								
Coma	3	0.3	8	<0.1	2	0.3	1	0.2
Convulsions	4	0.4	14	<0.1	3	0.5	1	0.2
**Dehydration**								
Diarrhoea	30	2.6	5,040	12.4	16	2.7	14	2.6
Lethargic or unconscious	0	0	18	0.4	0	0	0	0
Sunken eyes	0	0	46	0.9	0	0	0	0
Very slow skin pinch	0	0	9	0.2	0	0	0	0
**Priority signs**								
Tiny baby	0	0	36	<0.1	0	0	0	0
Temperature	84	7.3	9,928	24.3	46	7.7	38	7.0
Trauma	514	45.0	920	2.3	294	49.2	220	40.3
Severe pallor	0	0	55	0.1	0	0	0	0

Poisoning	2	0.2	11	<0.1	1	0.2	1	0.2
Severe pain	105	9.2	750	1.8	58	9.7	47	8.6
Respiratory distress	2	0.2	128	0.3	2	0.3	0	0
Restless/lethargic	1	<0.1	29	<0.1	0	0	1	0.2
Urgent referral	1	<0.1	7	<0.1	1	0.2	0	0
Malnutrition	0	0	6	<0.1	0	0	0	0
Oedema	4	0.3	8	<0.1	2	0.3	2	0.4
Burns	10	0.9	67	0.2	2	0.3	8	1.5

Supplementary Table 3. Referrals, and successful referrals by triage outcome and age group

Age group	Triage outcome	Referred		Not referred		Successfully referred		Not successfully referred	
		N	%	N	%	N	%	N	%
Overall	Emergency	97/ 3,004	3.2	89/ 152,927	0.1	52/ 1,644	3.2	45/ 1,361	3.3
	Priority	2,126/ 3,004	70.8	31,507/ 152,927	20.6	1,216/ 1,644	74.0	911/ 1,361	66.9
	Queue	781/ 3,002	26.0	121,331/ 152,927	79.3	376/ 1,644	22.9	405/ 1,361	29.8
<2 months	Emergency	2/ 137	1.5	5/ 3,517	0.1	0/ 72	0	2/ 65	3.0
	Priority	126/ 137	92.0	1,879	53.4	67/ 72	93.1	59/ 65	90.8
	Queue	9/ 137	6.6	1,633	46.4	5/ 72	6.9	4/ 65	6.2
2-12 months	Emergency	55/ 690	8.0	23/ 57,647	<0.1	32/ 393	8.1	23/ 297	7.7
	Priority	452/ 690	65.5	10,918/ 57,647	18.9	267/ 393	67.9	185/ 297	62.3
	Queue	183/ 690	26.5	46,706/ 57,647	81.0	94/ 393	23.9	89/ 297	30.0
>1-5 years	Emergency	28/ 1,034	2.7	29/ 51,030	0.1	14/ 582	2.4	14/ 452	3.1
	Priority	737/ 1,034	71.3	10,827/ 51,030	21.2	436/ 582	74.9	301/ 452	66.6
	Queue	269/ 1,034	26.0	40,174/ 51,030	78.7	132/ 582	22.7	137/ 452	30.3
>5 years	Emergency	12/ 1,143	1.0	32/ 40,733	0.1	6/ 597	1.0	6/ 546	1.1
	Priority	811/ 1,143	71.0	7,883/ 40,733	19.4	446/ 597	74.7	365/ 546	66.8
	Queue	320/ 1,143	28.0	32,818/ 40,733	80.6	145/ 597	24.3	175/ 546	32.1
