# Supplementary material for: Child acute illness presentation and referrals at primary health clinics in Malawi: a secondary analysis of ASPIRE
Source: BMJ Open. 2024 Apr 25;14(4):e079589. doi: 10.1136/bmjopen-2023-079589 (PMC11057250; doi:10.1136/bmjopen-2023-079589)
Supplement: Author's manuscript [file bmjopen-2023-079589.draft_revisions.pdf]

BMJ Open is committed to open peer review. As part of this commitment we make the peer review history of every article we publish publicly available.

When an article is published we post the peer reviewers' comments and the authors' responses online. We also post the versions of the paper that were used during peer review. These are the versions that the peer review comments apply to.

The versions of the paper that follow are the versions that were submitted during the peer review process. They are not the versions of record or the final published versions. They should not be cited or distributed as the published version of this manuscript.

BMJ Open is an open access journal and the full, final, typeset and author-corrected version of record of the manuscript is available on our site with no access controls, subscription charges or pay-per-view fees (http://bmjopen.bmj.com).

If you have any questions on BMJ Open's open peer review process please email info.bmjopen@bmj.com

Child acute illness presentation to primary health clinics and referrals in urban Blantyre, Malawi: a secondary analysis of ASPIRE

Journal:	*BMJ Open*
Manuscript ID	bmjopen-2023-079589
Article Type:	Original research
Date Submitted by the Author:	05-Sep-2023

Complete List of Authors:	Iroh Tam, Pui-Ying ; Malawi-Liverpool-Wellcome Trust Clinical Research Programme, ; Liverpool School of Tropical Medicine,<br>Twabi, Hussein; Malawi-Liverpool-Wellcome Trust Clinical Research Programme<br>Gondwe, Mtisunge; Malawi-Liverpool-Wellcome Trust Clinical Research Programme<br>O'Byrne, Thomasena; Malawi-Liverpool-Wellcome Trust Clinical Research Programme<br>Lufesi, Norman; Government of Malawi Ministry of Health, Acute Respiratory Illness Unit<br>Desmond, Nicola; Liverpool School of Tropical Medicine, International Public Health

Keywords:	PAEDIATRICS, EPIDEMIOLOGY, International health services < HEALTH SERVICES ADMINISTRATION & MANAGEMENT, Community child health < PAEDIATRICS, Primary Care < Primary Health Care, PUBLIC HEALTH

SCHOLARONE™
Manuscripts

I, the Submitting Author has the right to grant and does grant on behalf of all authors of the Work (as defined in the below author licence), an exclusive licence and/or a non-exclusive licence for contributions from authors who are: i) UK Crown employees; ii) where BMJ has agreed a CC-BY licence shall apply, and/or iii) in accordance with the terms applicable for US Federal Government officers or employees acting as part of their official duties; on a worldwide, perpetual, irrevocable, royalty-free basis to BMJ Publishing Group Ltd ("BMJ") its licensees and where the relevant Journal is co-owned by BMJ to the co-owners of the Journal, to publish the Work in this journal and any other BMJ products and to exploit all rights, as set out in our licence.

The Submitting Author accepts and understands that any supply made under these terms is made by BMJ to the Submitting Author unless you are acting as an employee on behalf of your employer or a postgraduate student of an affiliated institution which is paying any applicable article publishing charge ("APC") for Open Access articles. Where the Submitting Author wishes to make the Work available on an Open Access basis (and intends to pay the relevant APC), the terms of reuse of such Open Access shall be governed by a Creative Commons licence – details of these licences and which Creative Commons licence will apply to this Work are set out in our licence referred to above.

Other than as permitted in any relevant BMJ Author's Self Archiving Policies, I confirm this Work has not been accepted for publication elsewhere, is not being considered for publication elsewhere and does not duplicate material already published. I confirm all authors consent to publication of this Work and authorise the granting of this licence.

For peer review only

Child acute illness presentation to primary health clinics and referrals in urban Blantyre, Malawi: a secondary analysis of ASPIRE

Pui-Ying Iroh Tam, DMed,[1,2,3*] Hussein H Twabi, MSc,[1,3,5] Mtisunge Gondwe, PhD,[1,3] Thomasena O'Byrne, PhD,[1,2] Norman Lufesi, MPhil,[4] Nicola Desmond, PhD[2]

*Corresponding author: irohtam@mlw.mw

1. Malawi-Liverpool-Wellcome Research Programme, Blantyre, Malawi
2. Liverpool School of Tropical Medicine, Liverpool, United Kingdom
3. Kamuzu University of Health Sciences, Blantyre, Malawi
4. Ministry of Health Malawi, Lilongwe, Malawi
5. University of Liverpool, Liverpool, United Kingdom

Word count: 2,441

For peer review only

Introduction: We aimed to assess the prevalence, presentation, and referral patterns of children with acute illness attending primary health centres (PHCs) in a low-resource setting.

Methods: We conducted a secondary analysis of ASPIRE, which included an integrated clinician and mobile health (mHealth) data surveillance system following primary-level Emergency Triage Assessment and Treatment (ETAT) guidelines set up at eight PHCs in urban Blantyre district in southern Malawi, and supported through an in-house data platform to document the identification, prioritization, and referral of severe illness in children.

Results: Between April 2017 to September 2018, a total of 204,924 children were triaged, of whom 155,931 had both recorded clinician and mHealth triage data. The proportion of emergency and priority clinician triage were highest among young infants <2 months ((0·2%) and 81·4%, respectively). The most common presenting symptoms at PHCs were fever (0·3%), cough (0·2%), and difficulty breathing (0·2%). The most common signs associated with referral for under-5 children were trauma (26·7%) and temperature (7·4%). Of the 3,004 referrals (1·9%), 1,644 were successful (54·7%). Additionally, 372 children were sent home from PHC who subsequently self-referred to the referral facility (18·7%).

Conclusions: Fever and respiratory symptoms were the most common presenting symptoms, and trauma was the most common reason for referral. Rates of referral were low, and of successful referral were moderate. Self-referrals constituted a substantial proportion of attendance at the referral facility. Reducing gaps in care and addressing dropouts as well as self-referrals along the referral pathway could improve child health outcomes.

Word count: 248

Keywords: primary level; child; global health

For peer review only

What is already known on this topic:

- In low-resource settings, presenting symptoms and referral patterns of children at primary health centres (PHCs) in sub-Saharan Africa have been based primarily on qualitative research and have been poorly quantified.

What this study adds:

- The current study is, to our knowledge, the first to prospectively and systematically quantify child acute illness presentation and referral patterns in an urban district setting in sub-Saharan Africa. Using a mHealth triage and surveillance platform at PHC and referral hospitals, we documented 155,931 children attending PHCs in urban Blantyre, and a referral rate of <2%. In addition, the mHealth patient surveillance system allowed us to not only track successful referrals to QECH, but also to identify and describe patients that presented to PHCs, were sent home, and then self-referred to the referral facility.

How this study might affect research, practice or policy:

- Referral of severely ill children to hospital is a key component of the Integrated Management of Childhood Illness (IMCI). Reducing gaps in care and addressing dropouts along the referral pathway could strengthen the implementation of IMCI, and improve child health outcomes.

For peer review only

Introduction

The leading cause of under-five deaths globally is preventable infections, namely pneumonia and diarrhoea,[1] and WHO developed the Integrated Management of Childhood Illness (IMCI)[2] to reduce mortality due to these causes. The guidelines provided by IMCI, the integrated Community Case Management (iCCM)[3] and Emergency Triage Assessment and Treatment (ETAT)[4] are part of a comprehensive effort to improve the quality of care of ill children at different levels of the health system in low-resource settings, and to promote access and early initiation of first-line treatment or referral of severe cases to inpatient facilities.

Referral is a critical component of the IMCI strategy, intended to recognise children with severe conditions and ensure appropriate management and optimise quality care at the proper level of the health system.[5,6] In low-resource settings in sub-Saharan Africa prior to the implementation of IMCI, <2% of children presenting at primary level facilities were referred;[5,7] after the introduction of IMCI, referral rates for children were 6-20%.[8] However, for children who have been referred, successful referral (i.e. arrival at the referral facility) has been documented to be as low as 28%[9] and can be hindered by multiple barriers, from parental education[6,10] to communication,[6] transport,[6,9,11,12] and cost.[9,11]

While social factors affect presentation at and referrals from PHCs, the clinical features, healthcare utilization, and referral patterns in this setting have been poorly quantified. Elucidating these features can help identify where to allocate scarce resources, and reduce inequities in access. We sought to determine the most common features of acute illness among under-five children presenting to primary health centres (PHCs) in an urban, low-

For peer review only

resource setting within reasonable proximity to a referral facility, and what features are related to referral and successful referral by age group.

Methods

Study design, setting, and participants

The current study is a secondary analysis of data from the Achieving Sustainable Primary Improvement and Engagement in health (ASPIRE) project. ASPIRE implemented a mobile health (mHealth) triage algorithm based on ETAT for primary-level care, and healthcare workers positioned at both PHCs and the referral facility (Queen Elizabeth Central Hospital – QECH) used the mHealth algorithm to document the characteristics of all patients up to 14 years of age, including their symptoms, investigations, triage outcomes, and referral statuses as they presented at these sites in urban Blantyre. In 2014, the district of Blantyre had a population of 1·9 million,[13] about half of whom were under 15 years of age, with the majority residing in peri-urban townships. QECH is a tertiary-level hospital and referral facility in the southern region of the country; patients are thus expected to first be seen in a PHC or district hospital before presentation to QECH.

Public involvement

Between 2013 and 2017, the ASPIRE mHealth primary ETAT was developed and tested, with the involvement of district and primary health level stakeholders and national policymakers, and then implemented from 2017 to 2018 across eight PHCs in Blantyre district in southern Malawi. Details of the study have been published previously.[14,15]

Procedures

For peer review only - http://bmjopen.bmj.com/site/about/guidelines.xhtml

An mHealth patient surveillance system was established to monitor patients from initial presentation at the PHC through referral to QECH and final outcome. Participants were entered into the surveillance system as they entered the PHC and assigned a unique identification number (ID), and the mHealth triage outcome following implementation of the algorithm would be recorded by the healthcare worker, together with the facility name, sex and age of the patient. After review by a clinician, the clinician triage and consultation outcome (sent home, admitted for short stay, or referred) were documented, as well as any laboratory tests that were conducted. For all referrals, patients were tracked through a standard referral stamp to arrival at QECH. At QECH, presenting features, laboratory tests conducted, final diagnosis, and outcome (alive, died, absconded) were documented. A referral was defined as any patient referred from the PHC (either clinic or short stay); successful referral was defined as any patient referred from PHC who arrived at QECH; and self-referral was defined as any patient sent home from PHC who subsequently arrived at QECH.

Ethical approval for this study was obtained from the College of Medicine Research Ethics Committee (P·09/16/2021). Since the only patient identifier collected was age, patient-level consent was waived. Facility-level consent was obtained for data collection through the mHealth tool. All data were analysed at aggregate level and anonymised before analysis.

Statistical analysis

Prior to analysis, we removed records with invalid patient IDs and duplicated patient IDs that could not be matched unambiguously between data collection points. Records from the different data collection points were matched by patient IDs to allow for patient journey monitoring. Data for the different collection points were extracted as csv files from the

For peer review only - http://bmjopen.bmj.com/site/about/guidelines.xhtml

database, then analysed in RStudio version 446 (2023·03·1) using R version 4·3·0 (2023-04-21). Descriptive statistics were used to describe the data. Continuous data were described using means and standard deviations (SD) when normally distributed, and medians and interquartile ranges (IQR) when non-normal. Categorical data was described using proportions.

Results

Between April 2017 to September 2018, a total of 204,924 children attended eight PHCs in urban Blantyre (Figures 1 and Supplementary Figure 1), and 155,931 had complete clinician and mHealth triage outcome data (Table 1). Of these, 3,654 (2·3%) were <2 months, 58,337 (37·4%) were 2-12 months, 52,064 (33·4%) were >1-5 years, and 41,876 (26·9%) were >5 years.

At PHCs, 392 (0·3%) received a mHealth triage of Emergency and 48,248 (30·9%) of Priority, and 186 received a clinician triage of Emergency (0·1%) and 33,633 (21·6%) of Priority (Figure 2). The proportion of emergency and priority clinician triage were highest among young infants <2 months (7 (0·2%) and 2,974 (81·4%) respectively).

At PHCs, the most common documented presenting symptoms were fever (515; 0·3%), cough (340; 0·2%), and difficulty breathing (309; 0·2%). The most commonly performed test was the malaria rapid diagnostic test (MRDT, 483; 0·3%).

There were a total of 3,004 referrals (1·9%) to QECH. Within age groups, 3·7% of young infants <2 months presenting were referred, compared to 1·2% of 2-12 months, 2·0% of >1-5 years, and 2·7% of >5 years (Supplementary Table 1). Infants 2-12 months comprised the

For peer review only - http://bmjopen.bmj.com/site/about/guidelines.xhtml

largest proportion of Emergency triage that were referred (8·0%; Supplementary Table 2). Of these referrals, 1,644 (54·7%) were successful. For children ≤5 years (Table 2), the most common emergency signs related to referral were diarrhoea (130; 7·0%), respiratory distress (35; 1·9%) and obstructed breathing (28; 1·5%), and the most common priority signs were trauma (497; 26·7%), temperature (229; 12·3%), and tiny baby (137; 7·4%); these were also the most common emergency and priority signs related to a successful referral.

At QECH, the most commonly documented diagnoses overall were pneumonia (52/2,016; 2·6%) and gastroenteritis (35/2,016; 1·7%). The most commonly performed tests at QECH were the HIV test (148/2,016; 7·3%), malaria parasite screen, and packed cell volume (127/2,016; 6·3% each).

There were 372 self-referrals, the majority of whom were >1-5 years (Table 3). Of these, 1 (1·3%) received a clinician triage of Emergency, 145 (39·0%) Priority, and 222 (59·7%) Queue. The most common symptoms at PHC among those self-referred were temperature (90; 24·2%) and diarrhoea (54; 14·5%), and the most common diagnoses at QECH was pneumonia (16; 4·3%), malaria (15; 4·0%), and gastroenteritis (14; 3·8%).

Of those successfully referred, 53 arrived by ambulance (3·2%), while 22 who self-referred also arrived by ambulance (5·9%) – this can occur in a setting where an ambulance can provide a dual function to provide local transport in the area.

Forty-one blood and 11 CSF cultures were collected at QECH and 16 were positive (5 blood and 11 CSF). Of the five positive blood cultures, three were triaged as Priority and referred, and two triaged as Queue, sent home, and subsequently self-referred (40%). Of the 11

For peer review only - http://bmjopen.bmj.com/site/about/guidelines.xhtml

positive CSF cultures, one was triaged as Emergency and referred, six triaged as Priority and three referred, and five triaged as Queue and two referred; the remainder (45%) were sent home and then self-referred to QECH. Seven of 16 positive cultures (44%) were among children 2 months of age or less.

Two infants 2 months of age died, both at QECH. One was a female that received a clinician triage of Queue at the PHC and was sent home, then self-referred to QECH. At QECH, she was noted to have fever, cough, difficulty breathing, and 'other' problem. CSF culture, blood culture, HIV, MRDT, and other labs were collected, and the child was given a diagnosis of 'other.' The other was a male who received a clinician triage of Priority and was sent home, then self-referred to QECH, where he was noted to have oedema and 'other' problem, and was given a diagnosis of 'other.' CSF culture, blood culture, HIV, MRDT, glucose, and 'other' labs were collected. CSF cultures were positive for both; however, as the PHC patient identifiers did not link up with the microbiology database, further information was not retrieved on the cultures.

For peer review only

Discussion

This study is, to our knowledge, is the largest description of child acute illness presentation at primary level clinics and the first to prospectively and comprehensively describe clinical features, and quantify healthcare utilisation and referral patterns in sub-Saharan Africa. We documented over 155,000 children attending PHCs in urban Blantyre, and noted a referral rate of <2%. Though infants 2-12 months had the highest absolute numbers of attendance, young infants <2 months accounted for the largest proportion of emergency and priority signs, and the highest proportion of referrals, with fever and respiratory symptoms being the most common symptoms.

For peer review only - http://bmjopen.bmj.com/site/about/guidelines.xhtml

Comprehensive collection of triage at PHCs and prospective tracking of referrals to successful arrival at the referral hospital in a low-resource setting has not been documented previously. Studies of referral patterns in Uganda, Tanzania, Democratic Republic of the Congo, and Nigeria have been based either on retrospective review of records, interviews, focus group discussions, or follow-up visits, and documented successful referral rates ranging from 0·6% to 67%.[5,9,16] The largest prospective evaluation of successful referral involved prereferral rectal artesunate among 6,400 children with suspected severe malaria in the Democratic Republic of the Congo, Nigeria, and Uganda, and determined referral completion with a 28-day follow-up visit.

This study is the first time that successful referrals have been quantified in a systematic way along the referral pathway. We used an mHealth patient surveillance system that allowed us not only to track successful referrals to QECH, but also to identify patients that had been seen in PHCs, sent home, and then self-referred to QECH. Self-referrals made up 18·7% of overall referrals, and to our knowledge is the first time this has been quantified prospectively. Self-referral patterns has been described in other sub-Saharan African settings but those studies have focused on bypassing PHCs entirely,[17-19] rather than self-referral after being sent home from PHC.

We found that young infants were not the largest proportion of attendees at PHCs, but they did account for the largest proportion of emergency and priority signs, and referrals. This supports findings from other studies of sub-Saharan Africa where, compared to those 2-59 months, a higher proportion of young infants <2 months were referred from first-level health facilities in Uganda, Tanzania, and Niger.[8] A systematic review of community-to-facility

For peer review only - http://bmjopen.bmj.com/site/about/guidelines.xhtml

neonatal referral rates in Africa reported high completion rates ranging from 74-93%.[20] Given that neonatal mortality remains high despite gains made by the Sustainable Development Goals, this highlights the importance of the health system referral pathway to support the care of the young infant age group, and suggests that allocation of resources should target young infants as a vulnerable population.

For a healthcare worker applying IMCI guidelines, IMCI symptom referral criteria has a demonstrated sensitivity of 42-74% and specificity of 93-99%.[21] In our study, the most common symptoms at PHC were fever followed by respiratory symptoms, which corresponds with the commonest diagnoses at the referral hospital of pneumonia and malaria. Malaria was investigated for the most frequently at PHCs and diagnosed commonly at tertiary level for children greater than two months of age. This reflects the continued importance of infectious diseases on child morbidity and mortality. Trauma accounted for a sizeable proportion of cases, becoming the predominant cause of referral after the first year of life, highlighting the role and burden of injury in child health.[22]

We collected data on triage, since this has been shown to reduce inpatient mortality by up to two-thirds in low-resource settings.[23] Notably, 59·8% (85/142) of Emergency, 5·3% (1,315/24,939) Priority, and 0·5% (461/88,974) Queue triage cases were referred, which would suggest that there are opportunities to strengthen the IMCI, ETAT, and referral training of healthcare workers. Key health system constraints lead to poor quality paediatric care,[24] and prior assessment of inpatient paediatric care in first-referral level hospitals in Kenya showed that case management guidelines are often not in line with national or international guidelines,[25] which may explain our findings. It is noteworthy that the two deaths that occurred were both in children 2-12 months of age that had been triaged at PHC,

For peer review only

one as Priority and the other as Queue, and then sent home before self-referring to QECH. Given that the Malawi under-5 mortality rate in 2017 was 51 per 1,000 live births, the documentation of only two deaths in this surveillance is low, and may reflect the limitations of the surveillance platform used, as well as prior observations that over half of child deaths were observed to occur at home, and that cases reaching the PHC and hospital may represent only the tip of the iceberg.[5]

This study has limitations. As part of the implementation work around the mHealth surveillance system, existing healthcare workers based at PHCs were trained to collected data as part of their routine duties, but were not research staff trained at the level to conduct a clinical trial. Therefore, the pragmatic approach resulted in issues of data completeness, and did not allow us to interrogate the data more closely. The referral pathway relied on tracking patients through a standard referral stamp; therefore, patients who bypassed the PHC triage system due to acute illness or injury, or who misplaced this stamp would therefore not be captured at the referral hospital, leading to underestimation of successful referrals. The mHealth surveillance system was based on patient identifiers that were separate from the QECH system, and therefore we were unable to retrieve the results of investigations to fully chronicle the patient journey and cross-check results.

Nevertheless, this study is one of the first to prospectively and systematically quantify child acute illness presentation, healthcare utilisation, and referral patterns in an urban district setting in a low-resource setting. A strong health system providing quality health service requires an effective referral pathway, to promote timely access, judicious allocation of human and physical resources, cost-effective care, and to optimise clinical outcomes. Our

For peer review only

For peer review only - http://bmjopen.bmj.com/site/about/guidelines.xhtml

study provides quantitative data that allows us to identify gaps in care, which can form the basis to strengthen the referral pathway and improve child health outcomes.

For peer review only

For peer review only - http://bmjopen.bmj.com/site/about/guidelines.xhtml

Figure legend

Figure 1. ASPIRE flow diagram of triage and referral from primary health centre to tertiary facility, by age group

Figure 2. Proportion triaged as Emergency, Priority, and Queue, by age group and by facility

Table 1. Characteristics of children attending primary health centre

Table 2. Characteristics of triage outcome and emergency and priority signs with referral for children attending primary health centre for children ≤5 years

Table 3. Characteristics of children discharged from the primary health centre with subsequent self-referral to hospital

Appendix

Supplementary Figure 1. Attendance at primary health centre in Blantyre district, by age group

Supplementary Table 1. Emergency and priority signs, triage outcome, referral, and successful referral for A) <2 months; B) 2-12 months; C) >1-5 years; D) >5 years

Supplementary Table 2. Referrals, and successful referrals by triage outcome and age group

Acknowledgements

We thank Marc Henrion for assistance with the ASPIRE dataset and statistical analysis.

Funding

Meningitis Research Foundation, the Scottish Government, Irish AID, and Wellcome.

Data sharing statement

An anonymised, de-identified version of the dataset can be made available on request to allow all results to be reproduced. All requests should be directed to the corresponding author.

For peer review only

References

1. Liu L, Oza S, Hogan D, et al. Global, regional, and national causes of under-5 mortality in 2000-15: an updated systematic analysis with implications for the Sustainable Development Goals. *Lancet* 2016; **388**(10063): 3027-35.
2. WHO. Integrated management of childhood illness chart booklet. 2014.
3. WHO. Joint statement on integrated community case management. Geneva, Switzerland: WHO and UNICEF; 2012.
4. WHO. Emergency Triage Assessment and Treatment (ETAT) course. Geneva, Switzerland: WHO; 2005.
5. Font F, Quinto L, Masanja H, et al. Paediatric referrals in rural Tanzania: the Kilombero District Study - a case series. *BMC Int Health Hum Rights* 2002; **2**(1): 4.
6. Kaunda W, Umali T, Chirwa ME, Nyondo-Mipando AL. Assessing Facilitators and Barriers to Referral of Children Under the Age of Five Years at Ndirande Health Centre in Blantyre, Malawi. *Glob Pediatr Health* 2021; **8**: 2333794X211051815.
7. Nordberg E, Holmberg S, Kiugu S. Exploring the interface between first and second level of care: referrals in rural Africa. *Trop Med Int Health* 1996; **1**(1): 107-11.
8. Simoes EA, Peterson S, Gamatie Y, et al. Management of severely ill children at first-level health facilities in sub-Saharan Africa when referral is difficult. *Bull World Health Organ* 2003; **81**(7): 522-31.
9. Peterson S, Nsungwa-Sabiiti J, Were W, et al. Coping with paediatric referral--Ugandan parents' experience. *Lancet* 2004; **363**(9425): 1955-6.
10. al Fadil SM, Alrahman SH, Cousens S, et al. Integrated Management of Childhood Illnesses strategy: compliance with referral and follow-up recommendations in Gezira State, Sudan. *Bull World Health Organ* 2003; **81**(10): 708-16.
11. Hongoro C, Musonza TG, Macq J, Anozie A. A qualitative assessment of the referral system at district level in Zimbabwe: implications on efficiency and effective delivery of health services. *Cent Afr J Med* 1998; **44**(4): 93-7.
12. Ifeanyichi M, Broekhuizen H, Cheelo M, et al. Surgical ambulance referrals in sub-Saharan Africa - financial costs and coping strategies at district hospitals in Tanzania, Malawi and Zambia. *BMC Health Serv Res* 2021; **21**(1): 728.
13. Review WP. 2014. https://worldpopulationreview.com/ (accessed 28 February 2019).
14. Gondwe MJ, Henrion MYR, O'Byrne T, et al. Clinical diagnosis in paediatric patients at urban primary health care facilities in southern Malawi: a longitudinal observational study. *BMC Health Serv Res* 2021; **21**(1): 150.
15. Majamanda MD, Joshua Gondwe M, Makwero M, et al. Capacity Building for Health Care Workers and Support Staff in Pediatric Emergency Triage Assessment and Treatment (ETAT) at Primary Health Care Level in Resource Limited Settings: Experiences from Malawi. *Compr Child Adolesc Nurs* 2021: 1-16.
16. Brunner NC, Omoluabi E, Awor P, et al. Prereferral rectal artesunate and referral completion among children with suspected severe malaria in the Democratic Republic of the Congo, Nigeria and Uganda. *BMJ Glob Health* 2022; **7**(5).
17. Bell G, Macarayan EK, Ratcliffe H, et al. Assessment of Bypass of the Nearest Primary Health Care Facility Among Women in Ghana. *JAMA Netw Open* 2020; **3**(8): e2012552.
18. Eritero AC, Gebreslasie KZ, Asgedom AT, et al. Self-referrals and associated factors among laboring mothers at Dilla University Referral Hospital, Dilla, Gedeo Zone, Ethiopia: a cross-sectional study. *BMC Womens Health* 2022; **22**(1): 417.

For peer review only

19. Okoli H, Obembe T, Osungbade K, Adeniji F, Adewole D. Self-referral patterns among federal civil servants in oyo state, South-Western Nigeria. *Pan Afr Med J* 2017; **26**: 105.
20. Kozuki N, Guenther T, Vaz L, et al. A systematic review of community-to-facility neonatal referral completion rates in Africa and Asia. *BMC Public Health* 2015; **15**: 989.
21. Integrated management of childhood illness: conclusions. WHO Division of Child Health and Development. *Bull World Health Organ* 1997; **75 Suppl 1**(Suppl 1): 119-28.
22. WHO. Preventing injuries and violence: an overview. WHO. Geneva: WHO; 2022.
23. Molyneux E, Ahmad S, Robertson A. Improved triage and emergency care for children reduces inpatient mortality in a resource-constrained setting. *Bull World Health Organ* 2006; **84**(4): 314-9.
24. English M, Esamai F, Wasunna A, et al. Assessment of inpatient paediatric care in first referral level hospitals in 13 districts in Kenya. *Lancet* 2004; **363**(9425): 1948-53.
25. English M, Esamai F, Wasunna A, et al. Delivery of paediatric care at the first-referral level in Kenya. *Lancet* 2004; **364**(9445): 1622-9.

For peer review only

Table 1. Characteristics of children attending primary health centre

**Characteristics**	**Total**		**<2 months**		**2-12 months**		**>1-5 years**		**>5 years**	
	N=155,931	%	N=3,654	%	N=58,337	%	N=52,064	%	N=41,876	%
Male	77,915	50·0	1,889	51·7	29,885	51·2	26,204	50·3	19,937	47·6
Weight, kg (mean | SD)	15·34	8·33	4·09	1·22	8·48	2·39	13·74	3·42	24·59	7·18
**PHC**										
Bangwe	23,389	15·0	604	16·5	10,115	17·3	7,388	14·2	5,282	12·6
Chileka	13,266	8·5	236	6·5	4,309	7·4	4,694	9·0	4,027	9·6
Chilomoni	14,342	9·2	265	7·3	5,097	8·7	4,878	9·4	4,102	9·8
Limbe	23,099	14·8	713	19·5	10,313	17·7	7,564	14·5	4,509	10·8
Mpemba	12,388	7·9	207	5·7	3,277	5·6	3,904	7·5	5,000	11·9
Ndirande	38,243	24·5	912	25·0	13,799	23·6	13,210	25·4	10,342	24·7
South Lunzu	18,990	12·2	457	12·5	6,557	11·2	6,207	11·9	5,769	13·8
Zingwangwa	12,214	7·8	260	7·1	4,890	8·4	4,219	8·1	2,845	6·8
**PHC mHealth triage**										
Emergency	392	0·3	19	0·5	162	0·3	136	0·3	75	0·2
Priority	48,248	30·9	2,974	81·4	15,694	26·9	16,912	32·5	12,668	30·3
Queue	107,291	68·8	661	18·1	42,481	72·8	35,016	67·3	29,133	69·6
**PHC clinician triage**										
Emergency	186	0·1	7	0·2	78	0·1	57	0·1	44	0·1
Priority	33,633	21·6	2,005	54·9	11,370	19·5	11,564	22·2	8,694	20·8
Queue	122,112	78·3	1,642	44·9	46,889	80·4	40,443	77·7	33,138	79·1
**PHC symptoms**										
Fever	515	0·3	23	0·7	231	0·4	166	0·3	93	0·2
Convulsions	100	0·1	1	<0·1	26	<0·1	46	0·1	27	0·1
Diarrhoea	149	0·1	1	<0·1	86	0·1	37	0·1	25	0·1
Vomiting	271	0·2	9	0·2	127	0·2	85	0·2	50	0·1
Cough	340	0·2	24	0·7	201	0·3	74	0·1	41	0·1
Difficulty breathing	309	0·2	26	0·7	199	0·3	65	0·1	19	<0·1
Rash	102	0·1	5	0·1	26	<0·1	35	0·1	36	0·1
Pallor	37	<0·1	1	<0·1	10	<0·1	19	<0·1	7	<0·1
Jaundice	13	<0·1	2	0·1	3	<0·1	5	<0·1	3	<0·1
Oedema	27	<0·1	0	0	10	<0·1	11	<0·1	6	<0·1
Urination problems	13	<0·1	3	0·1	1	<0·1	6	<0·1	3	<0·1
Feeding problems	97	0·1	5	0·1	46	0·1	27	0·1	19	<0·1
Other	1,498	1·0	50	1·4	227	0·4	557	1·1	664	1·6
**PHC laboratory test**	503	0·3	10	0·3	203	0·3	168	0·3	122	0·3
Haemoglobin	46	<0·1	1	<0·1	11	<0·1	20	<0·1	14	<0·1
Glucose	7	<0·1	0	0	1	<0·1	4	<0·1	2	<0·1
MPS	16	<0·1	0	0	3	<0·1	9	<0·1	4	<0·1
MRDT	483	0·3	9	0·2	201	0·3	159	0·3	114	0·3
Urine	4	<0·1	0	0	0	0	3	<0·1	1	<0·1
**PHC outcome**										
Admitted to short stay	181	0·1	3	0·1	67	0·1	62	0·1	49	0·1

For peer review only

Referred after triage	2,974	1·9	136	3·7	680	1·2	1,020	2·0	1,138	2·7
Referred after short stay	517[a]	0·3	28	0·8	128	0·2	188	0·4	173	0·4
Total referred from HC	3,004	1·9	137	3·7	690	1·2	1,034	2·0	1,143	2·7
Successful referral[b]	1,644/ 3,004	54·7	72/ 137	52·6	393/ 690	57·0	582/ 1,034	56·3	597/ 1,143	52·2
Self-referral[c]	372	0·2	21	0·6	132	0·2	130	0·2	89	0·2
**QECH A&E**										
Successful referral arrived by ambulance	53/ 1,644	3·2	2/ 137	1·5	24/ 690	3·5	16/ 1,034	1·5	11/ 1,143	1·0
Self-referral arrived by ambulance	22/ 372	5·9	3/ 21	14·3	9/ 132	6·8	5/ 130	3·8	5/ 89	5·6
Total arrived by ambulance	75/ 2,016	3·7	5/ 93	5·4	33/ 525	6·3	21/ 712	4·4	16/ 686	2·3
**QECH symptoms**										
Fever	205/ 2,016	10·2	20/ 93	21·5	111/ 525	21·1	39/ 712	5·5	35/ 686	5·1
Convulsions	32/ 2,016	1·6	1/ 93	1·1	7/ 525	1·3	20/ 712	2·8	4/ 686	0·6
Diarrhoea	49/ 2,016	2·4	2/ 93	2·2	39/ 525	7·4	4/ 712	0·6	4/ 686	0·6
Vomiting	75/ 2,016	3·7	5/ 93	5·4	41/ 525	7·8	13/ 712	1·8	16/ 686	2·3
Cough	159/ 2,016	7·9	15/ 93	16·1	94/ 525	17·9	28/ 712	3·9	22/ 686	2·8
Difficulty breathing	148/ 2,016	7·3	15/ 93	16·1	89/ 525	17·0	25/ 712	3·5	19/ 686	3·2
Rash	7/ 2,016	0·3	0	0	4/ 525	0·8	3/ 712	0·4	0	0
Pallor	25/ 2,016	1·2	0	0	6/ 525	1·1	8/ 712	1·1	11/ 686	1·6
Jaundice	11/ 2,016	0·5	7/ 93	7·5	0	0	3/ 712	0·4	1/ 686	0·1
Oedema	16/ 2,016	0·8	0	0	7/ 525	1·3	3/ 712	0·4	6/ 686	0·9
Urination problems	3/ 2,016	0·1	1/ 93	1·1	0	0	1/ 712	0·1	1/ 686	0·1
Feeding problems	52/ 2,016	2·6	8/ 93	8·6	27/ 525	5·1	6/ 712	0·8	11/ 686	1·6
Other	117/ 2,016	5·8	10/ 93	10·8	42/ 525	8·0	26/ 712	3·7	39/ 686	5·7
**QECH diagnosis**										
Trauma	9/ 2,016	0·4	0	0	2/ 525	0·4	4/ 712	0·6	3/ 686	0·4
Gastroenteritis	35/ 2,016	1·7	2/ 93	2·2	29/ 525	5·5	2/ 712	0·3	2/ 686	0·3
Pneumonia	52/ 2,016	2·6	4/ 93	4·3	37/ 525	7·0	5/ 712	0·7	6/ 686	0·9
Meningitis	6/ 2,016	0·3	0	0	2/ 525	0·4	3/ 712	0·4	1/ 686	0·1
Malnutrition	25/ 2,016	1·2	1/ 93	1·1	16/ 525	3·0	4/ 712	0·6	4/ 686	0·6

For peer review only

Sepsis	16/ 2,016	0·8	13/ 93	14·0	3/ 525	0·6	0	0	0	0
Malaria	41/ 2,016	2·0	0	0	14/ 525	2·7	18/ 712	2·5	9/ 686	1·3
Anaemia	26/ 2,016	1·3	1/ 93	1·1	8/ 525	1·5	7/ 712	0·9	10/ 686	1·5
Other	201/ 2,016	10·0	19/ 93	2-·4	97/ 525	18·5	41/ 712	5·8	44/ 686	6·4
**QECH laboratory test**	193/ 2,016	9·6	18/ 93	19·4	97/ 525	18·5	47/ 712	5·8	36/ 686	5·2
CSF culture	11/ 2,016	0·5	4/ 93	4·3	2/ 525	0·4	3/ 712	0·4	2/ 686	0·3
Blood culture	41/ 2,016	2·0	10/ 93	10·8	15/ 525	2·9	5/ 712	0·7	11/ 686	1·6
Blood glucose	23/ 2,016	1·1	2/ 93	2·2	15/ 525	2·9	3/ 712	0·4	3/ 686	0·4
HIV	148/ 2,016	7·3	12/ 93	12·9	74/ 525	14·1	32/ 712	4·5	30/ 686	4·4
MRDT	90/ 2,016	4·5	7/ 93	7·5	42/ 525	8·0	22/ 712	3·1	19/ 686	2·8
MPS	127/ 2,016	6·3	13/ 93	14·0	66/ 525	12·6	23/ 712	3·2	25/ 686	3·6
PCV	127/ 2,016	6·3	13/ 93	14·0	67/ 525	12·8	22/ 712	3·1	25/ 686	3·6
Other	38/ 2,016	1·9	1/ 93	1·1	13/ 525	2·5	8/ 712	1·1	16/ 686	2·3
**QECH laboratory test result**										
Positive blood culture	5/ 41	12·2	1/ 10	10	0/ 15	0	1/ 5	20	3/ 11	27
Positive CSF culture	11/ 11	100	4/ 4	100	2/ 2	100	3/ 3	100	2/2	100
Positive HIV test	8/ 148	5·4	0/ 12	0	2/ 74	27	0/ 32	0	6/ 30	20
Positive MRDT	17/ 90	18·9	0/ 7	0	5/ 42	12	7/ 22	32	5/ 19	26
Positive MPS	19/ 127	15·0	0/ 13	0	5/ 66	8	7/ 23	30	5/ 25	20
Low blood glucose	5/ 23	21·7	1/ 2	50	2/ 15	14	0/ 3	0	2/ 3	67
PCV (mean | SD)	32·17	8·11	35·62	7·59	32·76	6·11	33·68	8·12	27·44	11·12
**QECH A&E outcome**										
Died	2/ 2290	0·1	0/ 119	0	2/ 663	0·1	0/ 770	0	0/ 738	0

A&E, accident and emergency; CSF, cerebrospinal fluid; HIV, human immunodeficiency virus; mHealth, mobile health; MRDT, malaria rapid diagnostic test; MPS, malaria parasite screen; PCV, packed cell volume; PHC, primary health centre; QECH, Queen Elizabeth Central Hospital; SD, standard deviation
[a]517 cases that were referred to QECH from PHC were still admitted to short stay at the PHC, where they were again referred to QECH.
[b]Referred by PHC and arrived at QECH.
[c]Not referred by PHC but arrived at QECH.

For peer review only

Table 2. Characteristics of triage outcome and emergency and priority signs with referral for children attending primary health centre for children ≤5 years

Characteristics	Referred		Not referred		Successfully referred		Not successfully referred		Self-referred	
	N=1,861	%	N=112,194	%	N=1,047	%	N=815	%	N=283	%
**Emergency signs**										
**Breathing**										
Obstructed breathing	28	1·5	102	0·1	19	1·8	9	1·1	0	0·0
Central cyanosis	1	0·1	4	<0·1	0	0	1	0·1	0	0
Respiratory distress	35	1·9	82	0·1	21	2·0	14	1·7	5	1·8
**Circulation**										
Cold hands	11	0·6	1,214	1·1	5	0·5	6	0·7	7	2·5
Capillary refill >3 seconds	0	0	8	<0·1	0	0	0	0	0	0
Weak and fast pulse	1	0·1	20	<0·1	1	0·1	0	0	0	0
**Consciousness**										
Coma	3	0·2	3	<0·1	1	0·1	2	0·2	1	0·4
Convulsions	24	1·3	29	<0·1	15	1·4	9	1·1	3	1·1
**Dehydration**										
Diarrhoea	130	7·0	22,321	19·9	85	8·1	45	5·5	48	17·0
Lethargic or unconscious	6	0·3	118	0·1	2	0·2	4	0·5	2	0·7
Sunken eyes	16	0·9	280	0·2	13	1·2	3	0·4	6	2·1
Very slow skin pinch	1	0·1	60	0·1	1	0·1	0	0	0	0·0
**Priority signs**										
Tiny baby	137	7·4	3,856	3·4	76	7·3	62	7·6	26	9·2
Temperature	229	12·3	26,390	23·5	136	13·0	93	11·4	74	26·1
Trauma	497	26·7	1,619	1·4	304	29·1	193	23·7	17	6·0
Severe pallor	3	0·2	241	0·2	0	0	3	0·4	0	0
Poisoning	4	0·2	19	<0·1	3	0·3	1	0·1	0	0
Severe pain	100	5·4	1,050	0·9	52	5·0	48	5·9	14	5·0
Respiratory distress	47	2·5	798	0·7	35	3·3	12	1·5	9	3·2
Restless/lethargic	5	0·3	125	0·1	4	0·4	1	0·1	3	1·1
Urgent referral	7	0·4	16	<0·1	3	0·3	4	0·5	0	0
Malnutrition	12	0·6	41	<0·1	6	0·6	6	0·7	2	0·7
Oedema	9	0·5	46	<0·1	3	0·3	6	0·7	0	0
Burns	55	0·3	274	0·2	19	1·8	36	4·4	2	0·7

For peer review only - http://bmjopen.bmj.com/site/about/guidelines.xhtml

**Triage outcome**										
Emergency	85	4·6	57	0·1	46	4·4	39	4·8	5	1·8
Priority	1,315	70·7	23,624	21·1	770	73·5	546	67·0	116	41·0
Queue	461	24·8	88,513	78·9	231	22·1	230	28·2	162	57·2

For peer review only

Table 3. Characteristics of children discharged from the primary health centre with subsequent self-referral to hospital

Characteristics	Total		<2 months		2-12 months		>1-5 years		>5 years	
	N=372	%	N=21	%	N=132	%	N=130	%	N=89	%
Male	215	57·9	9	42·9	72	54·5	72	55·4	62	69·7
Weight, kg (mean | SD)	11·80	4·18	-	-	4·90	-	13·03	1·95	15.00	-
**PHC mHealth triage**										
Emergency	11	3·0	0	0	5	3·8	5	3·8	1	1·1
Priority	197	53·0	21	100	68	51·5	58	44·6	50	56·2
Queue	164	44·1	0	0	59	44·7	67	51·5	38	42·7
**PHC clinician triage**										
Emergency	1	1·3	0	0	2	1·5	3	2·3	0	0
Priority	145	39·0	17	81·0	55	41·7	44	33·8	29	32·6
Queue	222	59·7	4	19·0	75	56·8	83	63·8	60	67·4
**Emergency signs**										
**Breathing**										
Obstructed breathing	1	0·3	0	0	0	0	0	0	1	1·1
Central cyanosis	0	0	0	0	0	0	0	0	0	0
Respiratory distress	5	1·3	0	0	2	1·5	3	2·3	0	0
**Circulation**										
Cold hands	9	2·4	0	0	5	3·8	2	1·5	2	2·2
Capillary refill >3 seconds	0	0	0	0	0	0	0	0	0	0
Weak and fast pulse	0	0	0	0	0	0	0	0	0	0
**Consciousness**										
Coma	1	0·3	0	0	1	0·8	0	0	0	0
Convulsions	3	0·8	0	0	2	1·5	1	0·8	0	0
**Dehydration**										
Diarrhoea	54	14·5	3	14·3	35	26·5	10	7·7	6	6·7
Lethargic or unconscious	2	0·5	0	0	1	0·8	1	0·8	0	0
Sunken eyes	6	1·6	0	0	5	3·8	1	0·8	0	0
Very slow skin pinch	0	0	0	0	0	0	0	0	0	0
**Priority signs**										
Tiny baby	26	7·0	19	90·5	6	4·5	1	0·8	0	0
Temperature	90	24·2	2	9·5	40	30·3	32	24·6	16	18·0
Trauma	36	9·7	0	0	5	3·8	12	9·2	19	21·3
Severe pallor	0	0	0	0	0	0	0	0	0	0
Poisoning	0	0	0	0	0	0	0	0	0	0
Severe pain	23	6·2	0	0	5	3·8	9	6·9	9	10·1
Respiratory distress	11	3·0	0	0	7	5·3	2	1·5	2	2·2
Restless/ lethargic	5	1·3	0	0	2	1·5	1	0·8	2	2·2
Urgent referral	1	0·3	0	0	0	0	0	0	1	1·1

For peer review only - http://bmjopen.bmj.com/site/about/guidelines.xhtml

For peer review only

Malnutrition	2	0·5	0	0	2	1·5	0	0	0	0
Oedema	0	0	0	0	0	0	0	0	0	0
Burns	3	0·8	0	0	1	0·8	1	0·8	1	1·1
**QECH diagnosis**										
Trauma	1	0·3	0	0	0	0	0	0	1	1·1
Gastroenteritis	14	3·8	1	4·8	12	9·1	1	0·8	0	0
Pneumonia	16	4·3	1	4·8	12	9·1	3	2·3	0	0
Meningitis	2	0·5	0	0	1	0·8	1	0·8	0	0
Malnutrition	10	2·7	0	0	6	4·5	2	1·5	2	2·2
Sepsis	4	1·1	3	14·3	1	0·8	0	0	0	0
Malaria	15	4·0	0	0	5	3·8	7	5·4	3	3·4
Anaemia	6	1·6	0	0	3	2·3	2	1·5	1	1·1
Other	55	14·8	4	19·0	25	18·9	12	9·2	14	15·7
**QECH laboratory test result**										
Positive blood culture	2	0·5	0	0	0	0	0	0	2	2·2
Positive CSF culture	6	1·6	1	4·8	2	1·5	2	1·5	1	1·1
Positive HIV test	0	0	0	0	0	0	0	0	0	0
Positive MRDT	7	1·9	0	0	2	1·5	3	2·3	2	2·2
Positive MPS	9	2·4	0	0	6	4·5	1	0·8	2	2·2
Low blood glucose	2	0·5	0	0	1	0·8	0	0	1	1·1
PCV, (mean | SD)	32·34	6·61	36·25	1·89	31·00	5·89	35·60	2·30	30·80	11·99
**QECH A&E outcome**										
Died	2	0·5	0	0	2	1·5	0	0	0	0

A&E, accident and emergency; CSF, cerebrospinal fluid; HIV, human immunodeficiency virus; mHealth, mobile health; MPS, malaria parasite smear; MRDT, malaria rapid diagnostic test; PCV, packed cell volume; PHC, primary health clinic; QECH, Queen Elizabeth Central Hospital

For peer review only - http://bmjopen.bmj.com/site/about/guidelines.xhtml

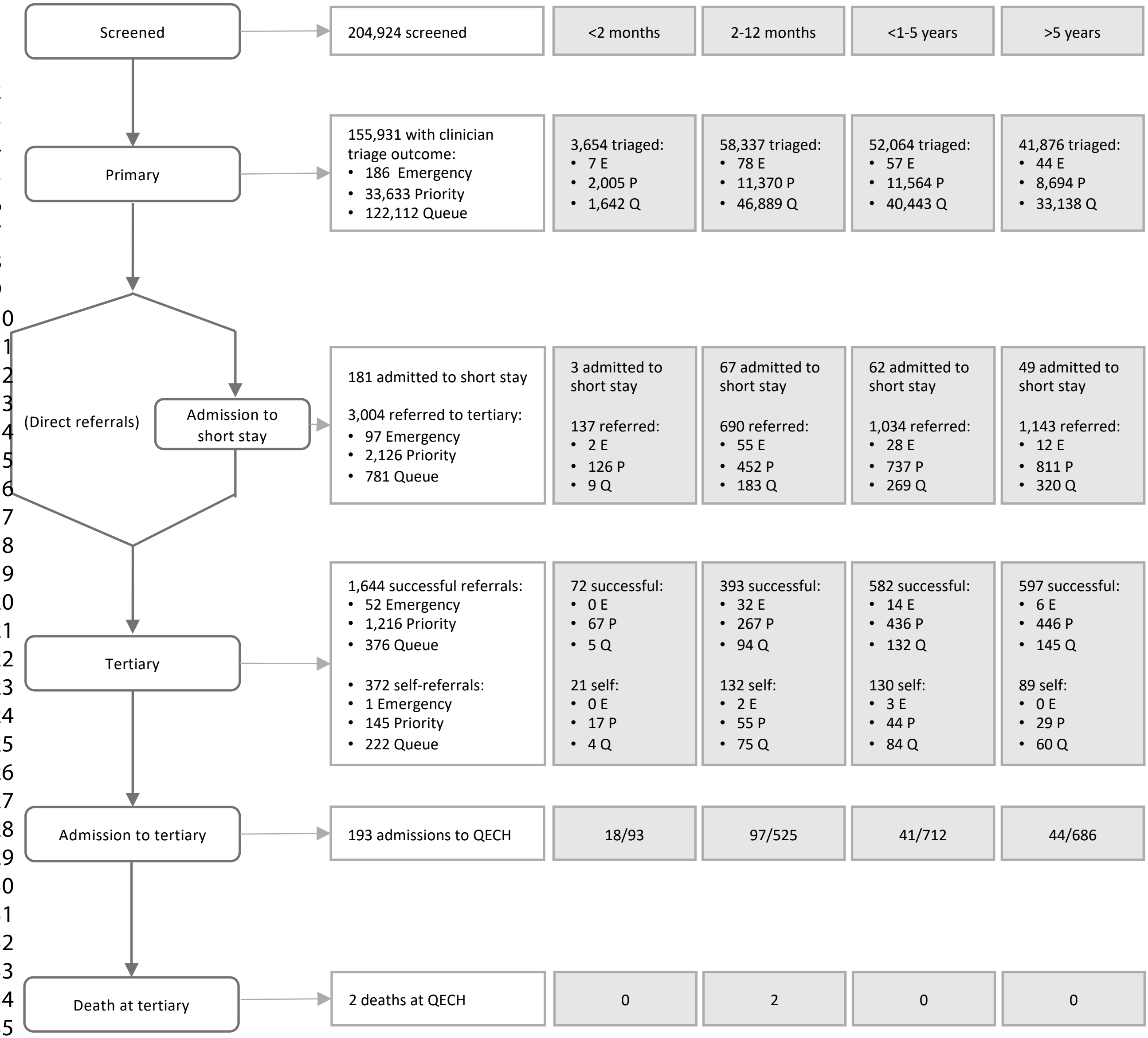
Screened
204,924 screened
<2 months
2-12 months
<1-5 years
>5 years
Primary
155,931 with clinician triage outcome:
• 186 Emergency
• 33,633 Priority
• 122,112 Queue
3,654 triaged:
• 7 E
• 2,005 P
• 1,642 Q
58,337 triaged:
• 78 E
• 11,370 P
• 46,889 Q
52,064 triaged:
• 57 E
• 11,564 P
• 40,443 Q
41,876 triaged:
• 44 E
• 8,694 P
• 33,138 Q
(Direct referrals)
Admission to short stay
181 admitted to short stay
3,004 referred to tertiary:
• 97 Emergency
• 2,126 Priority
• 781 Queue
3 admitted to short stay
137 referred:
• 2 E
• 126 P
• 9 Q
67 admitted to short stay
690 referred:
• 55 E
• 452 P
• 183 Q
62 admitted to short stay
1,034 referred:
• 28 E
• 737 P
• 269 Q
49 admitted to short stay
1,143 referred:
• 12 E
• 811 P
• 320 Q
Tertiary
1,644 successful referrals:
• 52 Emergency
• 1,216 Priority
• 376 Queue
• 372 self-referrals:
• 1 Emergency
• 145 Priority
• 222 Queue
72 successful:
• 0 E
• 67 P
• 5 Q
21 self:
• 0 E
• 17 P
• 4 Q
393 successful:
• 32 E
• 267 P
• 94 Q
132 self:
• 2 E
• 55 P
• 75 Q
582 successful:
• 14 E
• 436 P
• 132 Q
130 self:
• 3 E
• 44 P
• 84 Q
597 successful:
• 6 E
• 446 P
• 145 Q
89 self:
• 0 E
• 29 P
• 60 Q
Admission to tertiary
193 admissions to QECH
18/93
97/525
41/712
44/686
Death at tertiary
2 deaths at QECH
0
2
0
0

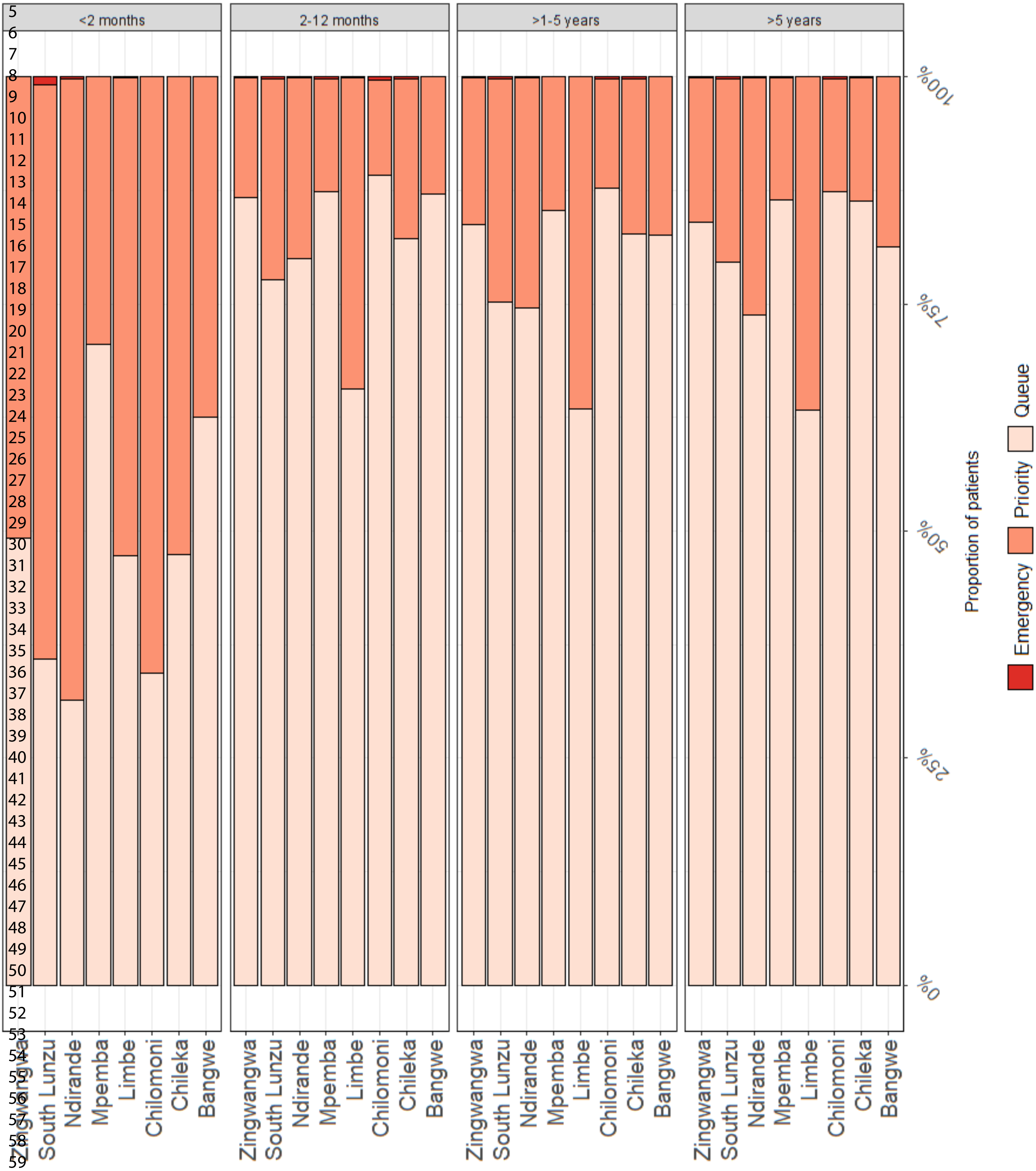
<2 months
2-12 months
>1-5 years
>5 years
Zingwangwa
South Lunzu
Ndirande
Mpemba
Limbe
Chilomoni
Chileka
Bangwe
0%
25%
50%
75%
100%
Proportion of patients
Emergency
Priority
Queue

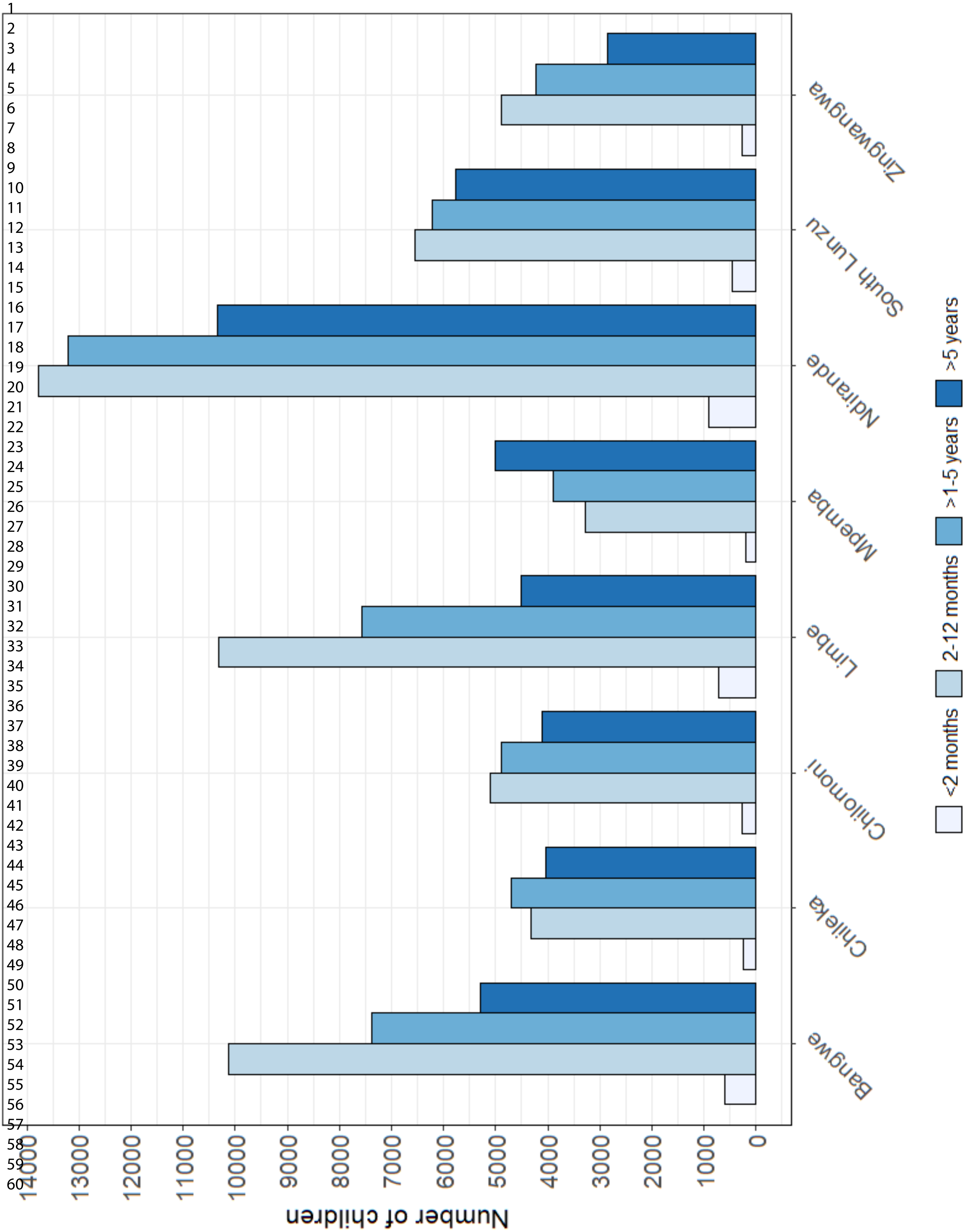
Zingwangwa
South Lunzu
Ndirande
Mpemba
Limbe
Chilomoni
Chileka
Bangwe
<2 months
2-12 months
>1-5 years
>5 years
Number of children
0
1000
2000
3000
4000
5000
6000
7000
8000
9000
10000
11000
12000
13000
14000

BMJ Open

Child acute illness presentation and referrals at primary health clinics in Malawi: a secondary analysis of ASPIRE

Journal:	*BMJ Open*
Manuscript ID	bmjopen-2023-079589.R1
Article Type:	Original research
Date Submitted by the Author:	21-Dec-2023
Complete List of Authors:	Iroh Tam, Pui-Ying ; Malawi-Liverpool-Wellcome Trust Clinical Research Programme, ; Liverpool School of Tropical Medicine,<br>Twabi, Hussein; Malawi-Liverpool-Wellcome Trust Clinical Research Programme<br>Gondwe, Mtisunge; Malawi-Liverpool-Wellcome Trust Clinical Research Programme<br>O'Byrne, Thomasena; Malawi-Liverpool-Wellcome Trust Clinical Research Programme<br>Lufesi, Norman; Government of Malawi Ministry of Health, Acute Respiratory Illness Unit<br>Desmond, Nicola; Liverpool School of Tropical Medicine, International Public Health
**Primary Subject Heading**:	Paediatrics
Secondary Subject Heading:	Global health, Public health
Keywords:	PAEDIATRICS, EPIDEMIOLOGY, International health services < HEALTH SERVICES ADMINISTRATION & MANAGEMENT, Community child health < PAEDIATRICS, Primary Care < Primary Health Care, PUBLIC HEALTH

SCHOLARONE™
Manuscripts

I, the Submitting Author has the right to grant and does grant on behalf of all authors of the Work (as defined in the below author licence), an exclusive licence and/or a non-exclusive licence for contributions from authors who are: i) UK Crown employees; ii) where BMJ has agreed a CC-BY licence shall apply, and/or iii) in accordance with the terms applicable for US Federal Government officers or employees acting as part of their official duties; on a worldwide, perpetual, irrevocable, royalty-free basis to BMJ Publishing Group Ltd ("BMJ") its licensees and where the relevant Journal is co-owned by BMJ to the co-owners of the Journal, to publish the Work in this journal and any other BMJ products and to exploit all rights, as set out in our licence.

The Submitting Author accepts and understands that any supply made under these terms is made by BMJ to the Submitting Author unless you are acting as an employee on behalf of your employer or a postgraduate student of an affiliated institution which is paying any applicable article publishing charge ("APC") for Open Access articles. Where the Submitting Author wishes to make the Work available on an Open Access basis (and intends to pay the relevant APC), the terms of reuse of such Open Access shall be governed by a Creative Commons licence – details of these licences and which Creative Commons licence will apply to this Work are set out in our licence referred to above.

Other than as permitted in any relevant BMJ Author's Self Archiving Policies, I confirm this Work has not been accepted for publication elsewhere, is not being considered for publication elsewhere and does not duplicate material already published. I confirm all authors consent to publication of this Work and authorise the granting of this licence.

For peer review only

Child acute illness presentation and referrals at primary health clinics in Malawi: a secondary analysis of ASPIRE

Pui-Ying Iroh Tam, DMed,[1,2,3*] Hussein H Twabi, MSc,[1,3,5] Mtisunge Gondwe, PhD,[1,3] Thomasena O'Byrne, PhD,[1,2] Norman Lufesi, MPhil,[4] Nicola Desmond, PhD[2]

*Corresponding author: irohtam@mlw.mw

1. Malawi-Liverpool-Wellcome Research Programme, Blantyre, Malawi
2. Liverpool School of Tropical Medicine, Liverpool, United Kingdom
3. Kamuzu University of Health Sciences, Blantyre, Malawi
4. Ministry of Health Malawi, Lilongwe, Malawi
5. University of Liverpool, Liverpool, United Kingdom

Word count: 2,599

For peer review only

Introduction: We aimed to assess the prevalence, presentation, and referral patterns of children with acute illness attending primary health centres (PHCs) in a low-resource setting.

Methods: We conducted a secondary analysis of ASPIRE. Children presenting at eight PHCs in urban Blantyre district in southern Malawi with both recorded clinician and mHealth (non-clinician) triage data were included, and patient records from different data collection points along the patient healthcare seeking pathway were consolidated and analysed.

Results: Between April 2017 to September 2018, a total of 204,924 children were triaged, of whom 155,931 had both recorded clinician and mHealth triage data. The most common presenting symptoms at PHCs were fever (0·3%), cough (0·2%), and difficulty breathing (0·2%). The most common signs associated with referral for under-5 children were trauma (26·7%) and temperature (7·4%). The proportion of emergency and priority clinician triage were highest among young infants <2 months ((0·2%) and 81·4%, respectively). Of the 3,004 referrals (1·9%), 1,644 successfully reached the referral facility (54·7%). Additionally, 372 children were sent home from PHC who subsequently self-referred to the referral facility (18·7%).

Conclusions: Fever and respiratory symptoms were the most common presenting symptoms, and trauma was the most common reason for referral. Rates of referral were low, and of successful referral were moderate. Self-referrals constituted a substantial proportion of attendance at the referral facility. Reducing gaps in care and addressing dropouts as well as self-referrals along the referral pathway could improve child health outcomes.

Word count: 240

For peer review only

Strengths and limitations of this study:

- This study prospectively and systematically quantifies child acute illness presentation and referral patterns in an urban district setting in sub-Saharan Africa.
- Analyses a mHealth triage and surveillance dataset of 155,931 children.
- Leverages a mHealth patient surveillance system that tracked those successful referred to the referral facility, as well as patients that presented to primary health centres, were sent home, and then later self-referred to the referral facility.
- Limitations were those inherent in a mHealth surveillance system reliant on existing healthcare workers rather than research-trained staff, including issues of data completeness.

Keywords: primary level; child; global health

For peer review only

Introduction

The leading global cause of mortality in children under five year is preventable infections, namely pneumonia and diarrhoea,[1] and WHO developed the Integrated Management of Childhood Illness (IMCI)[2] to reduce mortality due to these causes. The guidelines provided by IMCI, the integrated Community Case Management (iCCM)[3] and Emergency Triage Assessment and Treatment (ETAT)[4] are part of a comprehensive effort to improve the quality of care of ill children at different levels of the health system in low-resource settings, and to promote access and early initiation of first-line treatment or referral of severe cases to inpatient facilities, and to reduce mortality.[5-7]

Referral is a critical component of the IMCI strategy, intended to recognise children with severe conditions and ensure appropriate management and optimise quality care at the proper level of the health system.[8, 9] In Malawi in 2013-2014, only 3.5% of hospitals were equipped to provide basic paediatric emergency care in a national facility census.[10] In low-resource settings in sub-Saharan Africa prior to the implementation of IMCI, <2% of children presenting at primary level facilities were referred;[8, 11] after the introduction of IMCI, referral rates for children were 6-20%.[12] Challenges with the referral pathway range from parental education[9, 13] to communication,[9] transport,[9, 14-16] and cost.[14, 16] Therefore, for children who have been referred, successful referral (i.e. arrival at the referral facility) has been documented to be as low as 28%.[16]

While social factors affect presentation at and referrals from PHCs, the clinical features, healthcare utilization, and referral patterns in this setting have been poorly quantified. Elucidating these features can help identify where to allocate scarce resources, and reduce inequities in access. We sought to determine the most common features of acute illness

For peer review only - http://bmjopen.bmj.com/site/about/guidelines.xhtml

among children presenting to primary health centres (PHCs) in an urban, low-resource setting within reasonable proximity to a referral facility, and what features are related to referral and successful referral by age group.

Methods

Study design, setting, and participants

The current study is a secondary analysis of data from the Achieving Sustainable Primary Improvement and Engagement in health (ASPIRE) project. At the primary level in Malawi, clinical care is provided by clinically trained health workers (clinicians, nurses, medical assistants) and non-clinically trained health workers (health surveillance assistants, security guards, and other health centre staff). Human resource shortages mean that non-clinically trained health workers are often required to fill the gaps in service provision.

ASPIRE implemented a mobile health (mHealth) triage algorithm based on Emergency Triage and Treatment (ETAT) for primary-level care. For ASPIRE, non-clinical health workers positioned at both PHCs and the referral facility (Queen Elizabeth Central Hospital – QECH) were trained and used the mHealth algorithm to document the characteristics of all patients up to 14 years of age, including their symptoms, investigations, triage outcomes, and referral statuses as they presented at these sites in urban Blantyre. As part of the same PHC visit, data were first collected by the non-clinical health worker using the mHealth application (mHealth triage), and then separately by the clinical health worker (clinician triage).

In 2014, the district of Blantyre had a population of 1·9 million,[17] about half of whom were under 15 years of age, with the majority residing in peri-urban townships. QECH is a tertiary-

For peer review only - http://bmjopen.bmj.com/site/about/guidelines.xhtml

level hospital and referral facility in the southern region of the country; patients are thus expected to first be seen in a PHC or district hospital before presentation to QECH.

Public involvement

Between 2013 and 2017, the ASPIRE mHealth primary ETAT was developed and tested, with the involvement of district and primary health level stakeholders and national policymakers, and then implemented from 2017 to 2018 across eight PHCs in Blantyre district in southern Malawi. Details of the study have been published previously.[18, 19]

Procedures

An mHealth patient surveillance system was established to monitor patients from initial presentation at the PHC through referral to QECH and final outcome. Participants were entered into the surveillance system as they entered the PHC and assigned a unique identification number (ID), and the mHealth triage outcome following implementation of the algorithm would be recorded by the healthcare worker, together with the facility name, sex and age of the patient. After review by a clinician, the clinician triage and consultation outcome (sent home, admitted for short stay, or referred) were documented, as well as any laboratory tests that were conducted. For all referrals, patients were tracked through a standard referral stamp to arrival at QECH. At QECH, presenting features, laboratory tests conducted, final diagnosis, and outcome (alive, died, absconded) were documented. A referral was defined as any patient referred from the PHC (either clinic or short stay); successful referral was defined as any patient referred from PHC who arrived at QECH; and self-referral was defined as any patient sent home from PHC who subsequently arrived at QECH.

For peer review only - http://bmjopen.bmj.com/site/about/guidelines.xhtml

Ethical approval for this study was obtained from the College of Medicine Research Ethics Committee (P·09/16/2021). Since the only patient identifier collected was age, patient-level consent was waived. Facility-level consent was obtained for data collection through the mHealth tool. All data were analysed at aggregate level and anonymised before analysis.

Statistical analysis

Prior to analysis, we removed records with invalid patient IDs and duplicated patient IDs that could not be matched unambiguously between data collection points. Records from the different data collection points were matched by patient IDs to allow for patient journey monitoring. Data for the different collection points were extracted as csv files from the database, then analysed in RStudio version 446 (2023·03·1) using R version 4·3·0 (2023-04-21). Descriptive statistics were used to describe the data. Continuous data were described using means and standard deviations (SD) when normally distributed, and medians and interquartile ranges (IQR) when non-normal. Categorical data was described using proportions.

Results

Between April 2017 to September 2018, a total of 204,924 children attended eight PHCs in urban Blantyre (Figures 1 and Supplementary Figure 1), and 155,931 had complete clinician and mHealth triage outcome data (Table 1 and Supplementary Table 1). Of these, 3,654 (2·3%) were <2 months, 58,337 (37·4%) were 2-12 months, 52,064 (33·4%) were >1-5 years, and 41,876 (26·9%) were >5 years.

At PHCs, 392 (0·3%) received a mHealth triage of Emergency and 48,248 (30·9%) of Priority, and 186 received a clinician triage of Emergency (0·1%) and 33,633 (21·6%) of

For peer review only - http://bmjopen.bmj.com/site/about/guidelines.xhtml

Priority (Figure 2). The proportion of emergency and priority clinician triage were highest among young infants <2 months (7 (0·2%) and 2,974 (81·4%) respectively).

At PHCs, the most common documented presenting symptoms were fever (515; 0·3%), cough (340; 0·2%), and difficulty breathing (309; 0·2%). The most commonly performed test was the malaria rapid diagnostic test (MRDT, 483; 0·3%).

There were a total of 3,004 referrals (1·9%) to QECH. Within age groups, 3·7% of young infants <2 months presenting were referred, compared to 1·2% of 2-12 months, 2·0% of >1-5 years, and 2·7% of >5 years (Supplementary Table 2). Infants 2-12 months comprised the largest proportion of Emergency triage that were referred (8·0%; Supplementary Table 3). Of these referrals, 1,644 (54·7%) were successful. For children ≤5 years (Table 2), the most common emergency signs related to referral were diarrhoea (130; 7·0%), respiratory distress (35; 1·9%) and obstructed breathing (28; 1·5%), and the most common priority signs were trauma (497; 26·7%), temperature (229; 12·3%), and tiny baby (137; 7·4%); these were also the most common emergency and priority signs related to a successful referral.

At QECH, the most commonly documented diagnoses overall were pneumonia (52/2,016; 2·6%) and gastroenteritis (35/2,016; 1·7%). The most commonly performed tests at QECH were the HIV test (148/2,016; 7·3%), malaria parasite screen, and packed cell volume (127/2,016; 6·3% each).

There were 372 self-referrals, the majority of whom were >1-5 years (Table 3). Of these, 1 (1·3%) received a clinician triage of Emergency, 145 (39·0%) Priority, and 222 (59·7%) Queue. The most common symptoms at PHC among those self-referred were temperature

For peer review only

(90; 24·2%) and diarrhoea (54; 14·5%), and the most common diagnoses at QECH was pneumonia (16; 4·3%), malaria (15; 4·0%), and gastroenteritis (14; 3·8%).

Of those successfully referred, 53 arrived by ambulance (3·2%), while 22 who self-referred also arrived by ambulance (5·9%) – this can occur in a setting where an ambulance can provide a dual function to provide local transport in the area.

Forty-one blood and 11 CSF cultures were collected at QECH and 16 were positive (5 blood and 11 CSF). Of the five positive blood cultures, three were triaged as Priority and referred, and two triaged as Queue, sent home, and subsequently self-referred (40%). Of the 11 positive CSF cultures, one was triaged as Emergency and referred, six triaged as Priority and three referred, and five triaged as Queue and two referred; the remainder (45%) were sent home and then self-referred to QECH. Seven of 16 positive cultures (44%) were among children 2 months of age or less.

Two infants 2 months of age died, both at QECH. One was a female that received a clinician triage of Queue at the PHC and was sent home, then self-referred to QECH. At QECH, she was noted to have fever, cough, difficulty breathing, and 'other' problem. CSF culture, blood culture, HIV, MRDT, and other labs were collected, and the child was given a diagnosis of 'other.' The other was a male who received a clinician triage of Priority and was sent home, then self-referred to QECH, where he was noted to have oedema and 'other' problem, and was given a diagnosis of 'other.' CSF culture, blood culture, HIV, MRDT, glucose, and 'other' labs were collected. CSF cultures were positive for both; however, as the PHC patient identifiers did not link up with the microbiology database, further information was not retrieved on the cultures.

For peer review only - http://bmjopen.bmj.com/site/about/guidelines.xhtml

Discussion

This study is, to our knowledge, the largest description of child acute illness presentation at primary level clinics and the first to prospectively and comprehensively describe clinical features, and quantify healthcare utilisation and referral patterns in sub-Saharan Africa. We documented over 155,000 children attending PHCs in urban Blantyre, and noted a referral rate of <2%. Though infants 2-12 months had the highest absolute numbers of attendance, young infants <2 months accounted for the largest proportion of emergency and priority signs, and the highest proportion of referrals, with fever and respiratory symptoms being the most common symptoms.

Comprehensive collection of triage at PHCs and prospective tracking of referrals to successful arrival at the referral hospital in a low-resource setting has not been documented previously. Studies of referral patterns in Uganda, Tanzania, Democratic Republic of the Congo, and Nigeria have been based either on retrospective review of records, interviews, focus group discussions, or follow-up visits, and documented successful referral rates ranging from 0·6% to 67%.[8, 16, 20] The largest prospective evaluation of successful referral involved prereferral rectal artesunate among 6,400 children with suspected severe malaria in the Democratic Republic of the Congo, Nigeria, and Uganda, and determined referral completion with a 28-day follow-up visit.

This study is the first to report successful referrals that have been quantified in a systematic way. We used an mHealth patient surveillance system that allowed us not only to track successful referrals to QECH, but also to identify patients that had been seen in PHCs, sent home, and then self-referred to QECH. Self-referrals made up 18·7% of overall referrals, and

For peer review only - http://bmjopen.bmj.com/site/about/guidelines.xhtml

to our knowledge is the first time this has been quantified prospectively. Self-referral patterns has been described in other sub-Saharan African settings but those studies have focused on bypassing PHCs entirely,[21-23] rather than self-referral after being sent home from PHC.

We found that young infants were not the largest proportion of attendees at PHCs, but they did account for the largest proportion of emergency and priority signs, and referrals. This supports findings from other studies of sub-Saharan Africa where, compared to those 2-59 months, a higher proportion of young infants <2 months were referred from first-level health facilities in Uganda, Tanzania, and Niger.[12] A systematic review of community-to-facility neonatal referral rates in Africa reported high completion rates ranging from 74-93%.[24] Given that neonatal mortality remains high despite gains made by the Sustainable Development Goals, this highlights the importance of the health system referral pathway to support the care of the young infant age group, and suggests that allocation of resources should target young infants as a vulnerable population.

For a healthcare worker applying IMCI guidelines, IMCI symptom referral criteria has a demonstrated sensitivity of 42-74% and specificity of 93-99%.[25] In our study, the most common symptoms at PHC were fever followed by respiratory symptoms, which corresponds with the commonest diagnoses at the referral hospital of pneumonia and malaria. Malaria was investigated for the most frequently at PHCs and diagnosed commonly at tertiary level for children greater than two months of age. This reflects the continued importance of infectious diseases on child morbidity and mortality. Trauma accounted for a sizeable proportion of cases, becoming the predominant cause of referral after the first year of life, highlighting the role and burden of injury in child health.[26]

For peer review only

We collected data on triage, since this has been shown to reduce inpatient mortality by up to two-thirds in low-resource settings.[7] Notably, 59·8% (85/142) of Emergency, 5·3% (1,315/24,939) Priority, and 0·5% (461/88,974) Queue triage cases were referred, which would suggest that there are opportunities to strengthen the IMCI, ETAT, and referral training of healthcare workers. Key health system constraints lead to poor quality paediatric care,[27] and prior assessment of inpatient paediatric care in first-referral level hospitals in Kenya showed that case management guidelines are often not in line with national or international guidelines,[28] which may explain why there were lower than expected rates of referral. It is noteworthy that the two deaths that occurred were both in children 2-12 months of age that had been triaged at PHC, one as Priority and the other as Queue, and then sent home before self-referring to QECH. Given that the Malawi under-5 mortality rate in 2017 was 51 per 1,000 live births, the documentation of only two deaths in this surveillance is low, and may reflect the limitations of the surveillance platform used, as well as prior observations that over half of child deaths were observed to occur at home, and that cases reaching the PHC and hospital may represent only the tip of the iceberg.[8]

This study has limitations. As part of the implementation work around the mHealth surveillance system, existing healthcare workers based at PHCs were trained to collected data as part of their routine duties, but were not research staff trained at the level to conduct a clinical trial. Therefore, the pragmatic approach resulted in issues of data completeness, and did not allow us to interrogate the data more closely. The referral pathway relied on tracking patients through a standard referral stamp; therefore, patients who bypassed the PHC triage system due to acute illness or injury, or who misplaced this stamp would therefore not be captured at the referral hospital, leading to underestimation of successful referrals. The mHealth surveillance system was based on patient identifiers that were separate from the

For peer review only - http://bmjopen.bmj.com/site/about/guidelines.xhtml

For peer review only

QECH system, and therefore we were unable to retrieve the results of investigations to fully chronicle the patient journey and cross-check results.

Nevertheless, this study is one of the first to prospectively and systematically quantify child acute illness presentation, healthcare utilisation, and referral patterns in an urban district setting in a low-resource setting. A strong health system providing quality health service requires an effective referral pathway, to promote timely access, judicious allocation of human and physical resources, cost-effective care, and to optimise clinical outcomes. Our study provides quantitative data that allows us to identify gaps in care, which can form the basis to strengthen the referral pathway through regular monitoring, evaluation, and feedback to improve child health outcomes.

For peer review only

Figure legend

Figure 1. ASPIRE flow diagram of triage and referral from primary health centre to tertiary facility, by age group

Figure 2. Proportion triaged as Emergency, Priority, and Queue, by age group and by facility

Table 1. Characteristics of children attending primary health centre

Table 2. Characteristics of triage outcome and emergency and priority signs with referral for children attending primary health centre for children ≤5 years

Table 3. Characteristics of children discharged from the primary health centre with subsequent self-referral to hospital

Appendix

Supplementary Figure 1. Attendance at primary health centre in Blantyre district, by age group

Supplementary Table 1. Complete characteristics of children attending primary health centre

Supplementary Table 2. Emergency and priority signs, triage outcome, referral, and successful referral for A) <2 months; B) 2-12 months; C) >1-5 years; D) >5 years

Supplementary Table 3. Referrals, and successful referrals by triage outcome and age group

Acknowledgements

We thank Marc Henrion for assistance with the ASPIRE dataset and statistical analysis.

Funding

Meningitis Research Foundation (CSF 19-17 to ND), the Scottish Government (M/15/H/005 to ND), Irish AID, and Wellcome (206545/Z/17/Z).

Data sharing statement

An anonymised, de-identified version of the dataset can be made available on request to allow all results to be reproduced. All requests should be directed to the corresponding author.

Patient and public involvement statement

The ASPIRE mHealth primary ETAT was developed and tested, with the involvement of district and primary health level stakeholders and national policymakers.

Competing interest statement

All authors have no competing interests to disclose.

Contributor statement

PI conceived, led, and wrote the first draft of the manuscript. HHT conducted the statistical analysis. MG, TOB, NL, and ND led the primary ASPIRE study. All authors reviewed and approved the final manuscript.

For peer review only

References

1. Liu L, Oza S, Hogan D, Chu Y, Perin J, Zhu J, Lawn JE, Cousens S, Mathers C, Black RE: **Global, regional, and national causes of under-5 mortality in 2000-15: an updated systematic analysis with implications for the Sustainable Development Goals**. *Lancet* 2016, **388**(10063):3027-3035.
2. WHO: **Integrated management of childhood illness chart booklet**. 2014.
3. WHO: **Joint statement on integrated community case management**. In. Geneva, Switzerland: WHO and UNICEF; 2012.
4. WHO: **Emergency Triage Assessment and Treatment (ETAT) course**. In. Geneva, Switzerland: WHO; 2005.
5. Crouse HL, Torres F, Vaides H, Walsh MT, Ishigami EM, Cruz AT, Torrey SB, Soto MA: **Impact of an Emergency Triage Assessment and Treatment (ETAT)-based triage process in the paediatric emergency department of a Guatemalan public hospital**. *Paediatr Int Child Health* 2016, **36**(3):219-224.
6. Dekker-Boersema J, Hector J, Jefferys LF, Binamo C, Camilo D, Muganga G, Aly MM, Langa EBR, Vounatsou P, Hobbins MA: **Triage conducted by lay-staff and emergency training reduces paediatric mortality in the emergency department of a rural hospital in Northern Mozambique**. *Afr J Emerg Med* 2019, **9**(4):172-176.
7. Molyneux E, Ahmad S, Robertson A: **Improved triage and emergency care for children reduces inpatient mortality in a resource-constrained setting**. *Bull World Health Organ* 2006, **84**(4):314-319.
8. Font F, Quinto L, Masanja H, Nathan R, Ascaso C, Menendez C, Tanner M, Schellenberg J, Alonso P: **Paediatric referrals in rural Tanzania: the Kilombero District Study - a case series**. *BMC Int Health Hum Rights* 2002, **2**(1):4.
9. Kaunda W, Umali T, Chirwa ME, Nyondo-Mipando AL: **Assessing Facilitators and Barriers to Referral of Children Under the Age of Five Years at Ndirande Health Centre in Blantyre, Malawi**. *Glob Pediatr Health* 2021, **8**:2333794X211051815.
10. Johansson EW, Lindsjo C, Weiss DJ, Nsona H, Selling KE, Lufesi N, Hildenwall H: **Accessibility of basic paediatric emergency care in Malawi: analysis of a national facility census**. *BMC Public Health* 2020, **20**(1):992.
11. Nordberg E, Holmberg S, Kiugu S: **Exploring the interface between first and second level of care: referrals in rural Africa**. *Trop Med Int Health* 1996, **1**(1):107-111.
12. Simoes EA, Peterson S, Gamatie Y, Kisanga FS, Mukasa G, Nsungwa-Sabiiti J, Were MW, Weber MW: **Management of severely ill children at first-level health facilities in sub-Saharan Africa when referral is difficult**. *Bull World Health Organ* 2003, **81**(7):522-531.
13. al Fadil SM, Alrahman SH, Cousens S, Bustreo F, Shadoul A, Farhoud S, el Hassan SM: **Integrated Management of Childhood Illnesses strategy: compliance with referral and follow-up recommendations in Gezira State, Sudan**. *Bull World Health Organ* 2003, **81**(10):708-716.
14. Hongoro C, Musonza TG, Macq J, Anozie A: **A qualitative assessment of the referral system at district level in Zimbabwe: implications on efficiency and effective delivery of health services**. *Cent Afr J Med* 1998, **44**(4):93-97.
15. Ifeanyichi M, Broekhuizen H, Cheelo M, Juma A, Mwapasa G, Borgstein E, Kachimba J, Gajewski J, Brugha R, Pittalis C *et al*: **Surgical ambulance referrals in sub-Saharan Africa - financial costs and coping strategies at district hospitals in Tanzania, Malawi and Zambia**. *BMC Health Serv Res* 2021, **21**(1):728.

For peer review only - http://bmjopen.bmj.com/site/about/guidelines.xhtml

16. Peterson S, Nsungwa-Sabiiti J, Were W, Nsabagasani X, Magumba G, Nambooze J, Mukasa G: **Coping with paediatric referral--Ugandan parents' experience**. *Lancet* 2004, **363**(9425):1955-1956.
17. [https://worldpopulationreview.com/]
18. Gondwe MJ, Henrion MYR, O'Byrne T, Masesa C, Lufesi N, Dube Q, Majamanda MD, Makwero M, Lalloo DG, Desmond N: **Clinical diagnosis in paediatric patients at urban primary health care facilities in southern Malawi: a longitudinal observational study**. *BMC Health Serv Res* 2021, **21**(1):150.
19. Majamanda MD, Joshua Gondwe M, Makwero M, Chalira A, Lufesi N, Dube Q, Desmond N: **Capacity Building for Health Care Workers and Support Staff in Pediatric Emergency Triage Assessment and Treatment (ETAT) at Primary Health Care Level in Resource Limited Settings: Experiences from Malawi**. *Compr Child Adolesc Nurs* 2021:1-16.
20. Brunner NC, Omoluabi E, Awor P, Okitawutshu J, Tshefu Kitoto A, Signorell A, Akano B, Ayodeji K, Okon C, Yusuf O *et al*: **Prereferral rectal artesunate and referral completion among children with suspected severe malaria in the Democratic Republic of the Congo, Nigeria and Uganda**. *BMJ Glob Health* 2022, **7**(5).
21. Bell G, Macarayan EK, Ratcliffe H, Kim JH, Otupiri E, Lipsitz S, Hirschhorn L, Awoonor-Williams JK, Nimako BA, Ofosu A *et al*: **Assessment of Bypass of the Nearest Primary Health Care Facility Among Women in Ghana**. *JAMA Netw Open* 2020, **3**(8):e2012552.
22. Eritero AC, Gebreslasie KZ, Asgedom AT, Areba AS, Wudneh A, Bayisa Y, Molla W: **Self-referrals and associated factors among laboring mothers at Dilla University Referral Hospital, Dilla, Gedeo Zone, Ethiopia: a cross-sectional study**. *BMC Womens Health* 2022, **22**(1):417.
23. Okoli H, Obembe T, Osungbade K, Adeniji F, Adewole D: **Self-referral patterns among federal civil servants in oyo state, South-Western Nigeria**. *Pan Afr Med J* 2017, **26**:105.
24. Kozuki N, Guenther T, Vaz L, Moran A, Soofi SB, Kayemba CN, Peterson SS, Bhutta ZA, Khanal S, Tielsch JM *et al*: **A systematic review of community-to-facility neonatal referral completion rates in Africa and Asia**. *BMC Public Health* 2015, **15**:989.
25. **Integrated management of childhood illness: conclusions. WHO Division of Child Health and Development**. *Bull World Health Organ* 1997, **75 Suppl 1**(Suppl 1):119-128.
26. WHO: **Preventing injuries and violence: an overview**. In: *WHO*. Geneva: WHO; 2022.
27. English M, Esamai F, Wasunna A, Were F, Ogutu B, Wamae A, Snow RW, Peshu N: **Assessment of inpatient paediatric care in first referral level hospitals in 13 districts in Kenya**. *Lancet* 2004, **363**(9425):1948-1953.
28. English M, Esamai F, Wasunna A, Were F, Ogutu B, Wamae A, Snow RW, Peshu N: **Delivery of paediatric care at the first-referral level in Kenya**. *Lancet* 2004, **364**(9445):1622-1629.

For peer review only - http://bmjopen.bmj.com/site/about/guidelines.xhtml

Table 1. Main characteristics of children attending primary health centre

Characteristics	Total		<2 months		2-12 months		>1-5 years		>5 years	
	N=155,931	%	N=3,654	%	N=58,337	%	N=52,064	%	N=41,876	%
Male	77,915	50·0	1,889	51·7	29,885	51·2	26,204	50·3	19,937	47·6
Weight, kg (mean | SD)	15·34	8·33	4·09	1·22	8·48	2·39	13·74	3·42	24·59	7·18
**PHC mHealth triage**										
Emergency	392	0·3	19	0·5	162	0·3	136	0·3	75	0·2
Priority	48,248	30·9	2,974	81·4	15,694	26·9	16,912	32·5	12,668	30·3
Queue	107,291	68·8	661	18·1	42,481	72·8	35,016	67·3	29,133	69·6
**PHC clinician triage**										
Emergency	186	0·1	7	0·2	78	0·1	57	0·1	44	0·1
Priority	33,633	21·6	2,005	54·9	11,370	19·5	11,564	22·2	8,694	20·8
Queue	122,112	78·3	1,642	44·9	46,889	80·4	40,443	77·7	33,138	79·1
**PHC symptoms**										
Fever	515	0·3	23	0·7	231	0·4	166	0·3	93	0·2
Convulsions	100	0·1	1	<0·1	26	<0·1	46	0·1	27	0·1
Diarrhoea	149	0·1	1	<0·1	86	0·1	37	0·1	25	0·1
Vomiting	271	0·2	9	0·2	127	0·2	85	0·2	50	0·1
Cough	340	0·2	24	0·7	201	0·3	74	0·1	41	0·1
Difficulty breathing	309	0·2	26	0·7	199	0·3	65	0·1	19	<0·1
Rash	102	0·1	5	0·1	26	<0·1	35	0·1	36	0·1
Pallor	37	<0·1	1	<0·1	10	<0·1	19	<0·1	7	<0·1
Jaundice	13	<0·1	2	0·1	3	<0·1	5	<0·1	3	<0·1
Oedema	27	<0·1	0	0	10	<0·1	11	<0·1	6	<0·1
Urination problems	13	<0·1	3	0·1	1	<0·1	6	<0·1	3	<0·1
Feeding problems	97	0·1	5	0·1	46	0·1	27	0·1	19	<0·1
Other	1,498	1·0	50	1·4	227	0·4	557	1·1	664	1·6
**PHC laboratory test**	503	0·3	10	0·3	203	0·3	168	0·3	122	0·3
Haemoglobin	46	<0·1	1	<0·1	11	<0·1	20	<0·1	14	<0·1
Glucose	7	<0·1	0	0	1	<0·1	4	<0·1	2	<0·1
MPS	16	<0·1	0	0	3	<0·1	9	<0·1	4	<0·1
MRDT	483	0·3	9	0·2	201	0·3	159	0·3	114	0·3
Urine	4	<0·1	0	0	0	0	3	<0·1	1	<0·1
**PHC outcome**										
Admitted to short stay	181	0·1	3	0·1	67	0·1	62	0·1	49	0·1
Referred after triage	2,487	1·6	109	3·0	562	1·0	846	1·6	970	2·3
Referred after short stay	517[a]	0·3	28	0·8	128	0·2	188	0·4	173	0·4
Total referred from HC	3,004	1·9	137	3·7	690	1·2	1,034	2·0	1,143	2·7
Successful referral[b]	1,644/ 3,004	54·7	72/ 137	52·6	393/ 690	57·0	582/ 1,034	56·3	597/ 1,143	52·2
Self-referral[c]	372	0·2	21	0·6	132	0·2	130	0·2	89	0·2

For peer review only - http://bmjopen.bmj.com/site/about/guidelines.xhtml

**QECH A&E**										
Successful referral arrived by ambulance	53/ 1,644	3·2	2/ 137	1·5	24/ 690	3·5	16/ 1,034	1·5	11/ 1,143	1·0
Self-referral arrived by ambulance	22/ 372	5·9	3/ 21	14·3	9/ 132	6·8	5/ 130	3·8	5/ 89	5·6
Total arrived by ambulance	75/ 2,016	3·7	5/ 93	5·4	33/ 525	6·3	21/ 712	4·4	16/ 686	2·3
**QECH symptoms**										
Fever	205/ 2,016	10·2	20/ 93	21·5	111/ 525	21·1	39/ 712	5·5	35/ 686	5·1
Convulsions	32/ 2,016	1·6	1/ 93	1·1	7/ 525	1·3	20/ 712	2·8	4/ 686	0·6
Diarrhoea	49/ 2,016	2·4	2/ 93	2·2	39/ 525	7·4	4/ 712	0·6	4/ 686	0·6
Vomiting	75/ 2,016	3·7	5/ 93	5·4	41/ 525	7·8	13/ 712	1·8	16/ 686	2·3
Cough	159/ 2,016	7·9	15/ 93	16·1	94/ 525	17·9	28/ 712	3·9	22/ 686	2·8
Difficulty breathing	148/ 2,016	7·3	15/ 93	16·1	89/ 525	17·0	25/ 712	3·5	19/ 686	3·2
Rash	7/ 2,016	0·3	0	0	4/ 525	0·8	3/ 712	0·4	0	0
Pallor	25/ 2,016	1·2	0	0	6/ 525	1·1	8/ 712	1·1	11/ 686	1·6
Jaundice	11/ 2,016	0·5	7/ 93	7·5	0	0	3/ 712	0·4	1/ 686	0·1
Oedema	16/ 2,016	0·8	0	0	7/ 525	1·3	3/ 712	0·4	6/ 686	0·9
Urination problems	3/ 2,016	0·1	1/ 93	1·1	0	0	1/ 712	0·1	1/ 686	0·1
Feeding problems	52/ 2,016	2·6	8/ 93	8·6	27/ 525	5·1	6/ 712	0·8	11/ 686	1·6
Other	117/ 2,016	5·8	10/ 93	10·8	42/ 525	8·0	26/ 712	3·7	39/ 686	5·7
**QECH A&E outcome**										
Died	2/ 2290	0·1	0/ 119	0	2/ 663	0·1	0/ 770	0	0/ 738	0

A&E, accident and emergency; CSF, cerebrospinal fluid; HIV, human immunodeficiency virus; mHealth, mobile health; MRDT, malaria rapid diagnostic test; MPS, malaria parasite screen; PCV, packed cell volume; PHC, primary health centre; QECH, Queen Elizabeth Central Hospital; SD, standard deviation

[a]517 cases that were referred to QECH from PHC were still admitted to short stay at the PHC, where they were again referred to QECH.

[b]Referred by PHC and arrived at QECH.

[c]Not referred by PHC but arrived at QECH.

For peer review only

For peer review only - http://bmjopen.bmj.com/site/about/guidelines.xhtml

Table 2. Characteristics of triage outcome and emergency and priority signs with referral for children attending primary health centre for children ≤5 years

Characteristics	Referred		Not referred		Successfully referred		Not successfully referred		Self-referred	
	N=1,861	%	N=112,194	%	N=1,047	%	N=815	%	N=283	%
**Emergency signs**										
**Breathing**										
Obstructed breathing	28	1·5	102	0·1	19	1·8	9	1·1	0	0·0
Central cyanosis	1	0·1	4	<0·1	0	0	1	0·1	0	0
Respiratory distress	35	1·9	82	0·1	21	2·0	14	1·7	5	1·8
**Circulation**										
Cold hands	11	0·6	1,214	1·1	5	0·5	6	0·7	7	2·5
Capillary refill >3 seconds	0	0	8	<0·1	0	0	0	0	0	0
Weak and fast pulse	1	0·1	20	<0·1	1	0·1	0	0	0	0
**Consciousness**										
Coma	3	0·2	3	<0·1	1	0·1	2	0·2	1	0·4
Convulsions	24	1·3	29	<0·1	15	1·4	9	1·1	3	1·1
**Dehydration**										
Diarrhoea	130	7·0	22,321	19·9	85	8·1	45	5·5	48	17·0
Lethargic or unconscious	6	0·3	118	0·1	2	0·2	4	0·5	2	0·7
Sunken eyes	16	0·9	280	0·2	13	1·2	3	0·4	6	2·1
Very slow skin pinch	1	0·1	60	0·1	1	0·1	0	0	0	0·0
**Priority signs**										
Tiny baby	137	7·4	3,856	3·4	76	7·3	62	7·6	26	9·2
Temperature	229	12·3	26,390	23·5	136	13·0	93	11·4	74	26·1
Trauma	497	26·7	1,619	1·4	304	29·1	193	23·7	17	6·0
Severe pallor	3	0·2	241	0·2	0	0	3	0·4	0	0
Poisoning	4	0·2	19	<0·1	3	0·3	1	0·1	0	0
Severe pain	100	5·4	1,050	0·9	52	5·0	48	5·9	14	5·0
Respiratory distress	47	2·5	798	0·7	35	3·3	12	1·5	9	3·2
Restless/lethargic	5	0·3	125	0·1	4	0·4	1	0·1	3	1·1
Urgent referral	7	0·4	16	<0·1	3	0·3	4	0·5	0	0
Malnutrition	12	0·6	41	<0·1	6	0·6	6	0·7	2	0·7
Oedema	9	0·5	46	<0·1	3	0·3	6	0·7	0	0
Burns	55	0·3	274	0·2	19	1·8	36	4·4	2	0·7

For peer review only - http://bmjopen.bmj.com/site/about/guidelines.xhtml

**Triage outcome**										
Emergency	85	4·6	57	0·1	46	4·4	39	4·8	5	1·8
Priority	1,315	70·7	23,624	21·1	770	73·5	546	67·0	116	41·0
Queue	461	24·8	88,513	78·9	231	22·1	230	28·2	162	57·2

For peer review only

For peer review only - http://bmjopen.bmj.com/site/about/guidelines.xhtml

Table 3. Characteristics of children discharged from the primary health centre with subsequent self-referral to hospital

Characteristics	Total		<2 months		2-12 months		>1-5 years		>5 years	
	N=372	%	N=21	%	N=132	%	N=130	%	N=89	%
Male	215	57·9	9	42·9	72	54·5	72	55·4	62	69·7
Weight, kg (mean | SD)	11·80	4·18	-	-	4·90	-	13·03	1·95	15.00	-
**PHC mHealth triage**										
Emergency	11	3·0	0	0	5	3·8	5	3·8	1	1·1
Priority	197	53·0	21	100	68	51·5	58	44·6	50	56·2
Queue	164	44·1	0	0	59	44·7	67	51·5	38	42·7
**PHC clinician triage**										
Emergency	1	1·3	0	0	2	1·5	3	2·3	0	0
Priority	145	39·0	17	81·0	55	41·7	44	33·8	29	32·6
Queue	222	59·7	4	19·0	75	56·8	83	63·8	60	67·4
**Emergency signs**										
**Breathing**										
Obstructed breathing	1	0·3	0	0	0	0	0	0	1	1·1
Central cyanosis	0	0	0	0	0	0	0	0	0	0
Respiratory distress	5	1·3	0	0	2	1·5	3	2·3	0	0
**Circulation**										
Cold hands	9	2·4	0	0	5	3·8	2	1·5	2	2·2
Capillary refill >3 seconds	0	0	0	0	0	0	0	0	0	0
Weak and fast pulse	0	0	0	0	0	0	0	0	0	0
**Consciousness**										
Coma	1	0·3	0	0	1	0·8	0	0	0	0
Convulsions	3	0·8	0	0	2	1·5	1	0·8	0	0
**Dehydration**										
Diarrhoea	54	14·5	3	14·3	35	26·5	10	7·7	6	6·7
Lethargic or unconscious	2	0·5	0	0	1	0·8	1	0·8	0	0
Sunken eyes	6	1·6	0	0	5	3·8	1	0·8	0	0
Very slow skin pinch	0	0	0	0	0	0	0	0	0	0
**Priority signs**										
Tiny baby	26	7·0	19	90·5	6	4·5	1	0·8	0	0
Temperature	90	24·2	2	9·5	40	30·3	32	24·6	16	18·0
Trauma	36	9·7	0	0	5	3·8	12	9·2	19	21·3
Severe pallor	0	0	0	0	0	0	0	0	0	0
Poisoning	0	0	0	0	0	0	0	0	0	0
Severe pain	23	6·2	0	0	5	3·8	9	6·9	9	10·1
Respiratory distress	11	3·0	0	0	7	5·3	2	1·5	2	2·2
Restless/ lethargic	5	1·3	0	0	2	1·5	1	0·8	2	2·2
Urgent referral	1	0·3	0	0	0	0	0	0	1	1·1

For peer review only

Malnutrition	2	0·5	0	0	2	1·5	0	0	0	0
Oedema	0	0	0	0	0	0	0	0	0	0
Burns	3	0·8	0	0	1	0·8	1	0·8	1	1·1
**QECH diagnosis**										
Trauma	1	0·3	0	0	0	0	0	0	1	1·1
Gastroenteritis	14	3·8	1	4·8	12	9·1	1	0·8	0	0
Pneumonia	16	4·3	1	4·8	12	9·1	3	2·3	0	0
Meningitis	2	0·5	0	0	1	0·8	1	0·8	0	0
Malnutrition	10	2·7	0	0	6	4·5	2	1·5	2	2·2
Sepsis	4	1·1	3	14·3	1	0·8	0	0	0	0
Malaria	15	4·0	0	0	5	3·8	7	5·4	3	3·4
Anaemia	6	1·6	0	0	3	2·3	2	1·5	1	1·1
Other	55	14·8	4	19·0	25	18·9	12	9·2	14	15·7
**QECH laboratory test result**										
Positive blood culture	2	0·5	0	0	0	0	0	0	2	2·2
Positive CSF culture	6	1·6	1	4·8	2	1·5	2	1·5	1	1·1
Positive HIV test	0	0	0	0	0	0	0	0	0	0
Positive MRDT	7	1·9	0	0	2	1·5	3	2·3	2	2·2
Positive MPS	9	2·4	0	0	6	4·5	1	0·8	2	2·2
Low blood glucose	2	0·5	0	0	1	0·8	0	0	1	1·1
PCV, (mean | SD)	32·34	6·61	36·25	1·89	31·00	5·89	35·60	2·30	30·80	11·99
**QECH A&E outcome**										
Died	2	0·5	0	0	2	1·5	0	0	0	0

A&E, accident and emergency; CSF, cerebrospinal fluid; HIV, human immunodeficiency virus; mHealth, mobile health; MPS, malaria parasite smear; MRDT, malaria rapid diagnostic test; PCV, packed cell volume; PHC, primary health clinic; QECH, Queen Elizabeth Central Hospital

For peer review only

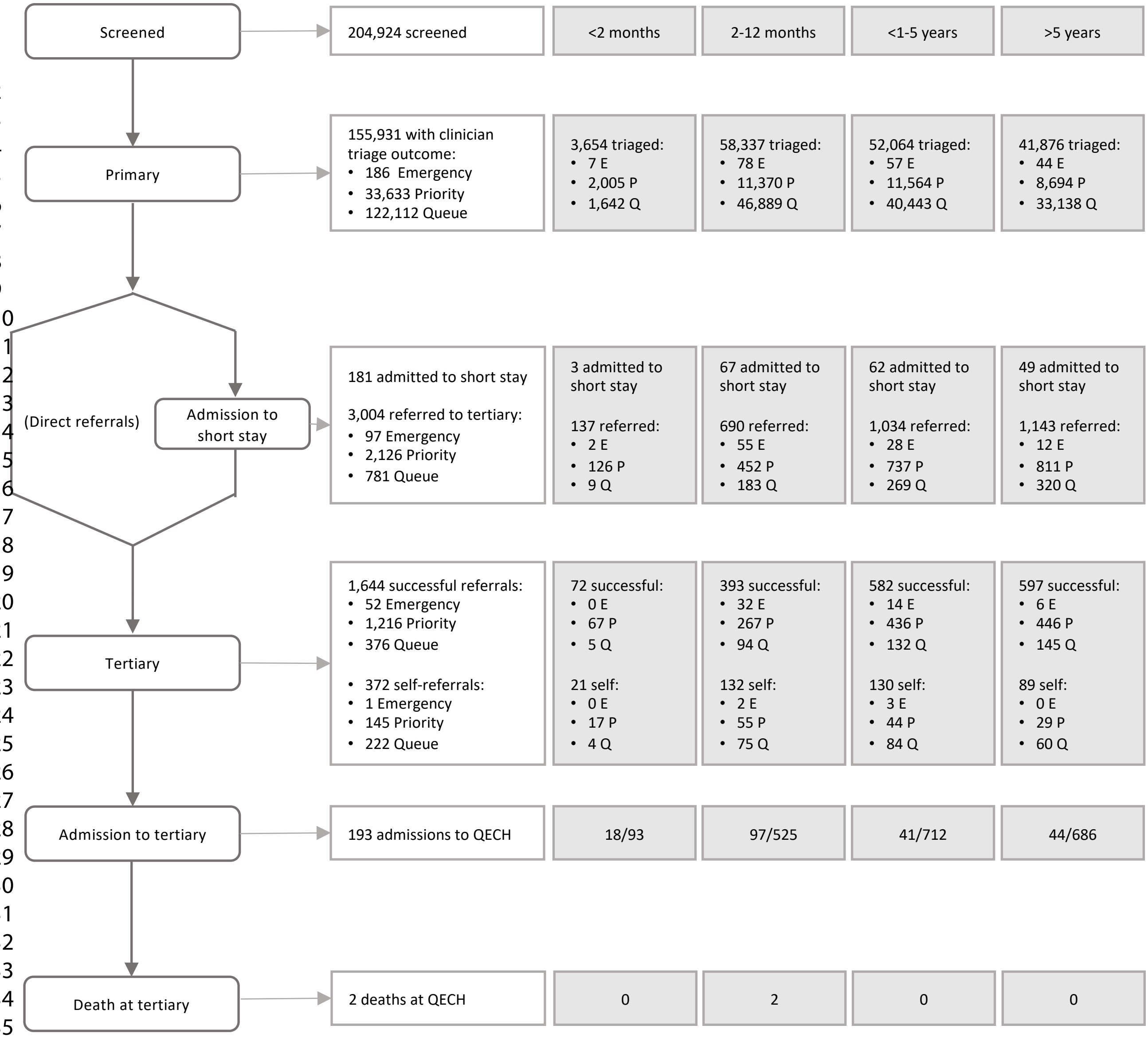
Screened
204,924 screened
<2 months
2-12 months
<1-5 years
>5 years
Primary
155,931 with clinician triage outcome:
• 186 Emergency
• 33,633 Priority
• 122,112 Queue
3,654 triaged:
• 7 E
• 2,005 P
• 1,642 Q
58,337 triaged:
• 78 E
• 11,370 P
• 46,889 Q
52,064 triaged:
• 57 E
• 11,564 P
• 40,443 Q
41,876 triaged:
• 44 E
• 8,694 P
• 33,138 Q
(Direct referrals)
Admission to short stay
181 admitted to short stay
3,004 referred to tertiary:
• 97 Emergency
• 2,126 Priority
• 781 Queue
3 admitted to short stay
137 referred:
• 2 E
• 126 P
• 9 Q
67 admitted to short stay
690 referred:
• 55 E
• 452 P
• 183 Q
62 admitted to short stay
1,034 referred:
• 28 E
• 737 P
• 269 Q
49 admitted to short stay
1,143 referred:
• 12 E
• 811 P
• 320 Q
Tertiary
1,644 successful referrals:
• 52 Emergency
• 1,216 Priority
• 376 Queue
• 372 self-referrals:
• 1 Emergency
• 145 Priority
• 222 Queue
72 successful:
• 0 E
• 67 P
• 5 Q
21 self:
• 0 E
• 17 P
• 4 Q
393 successful:
• 32 E
• 267 P
• 94 Q
132 self:
• 2 E
• 55 P
• 75 Q
582 successful:
• 14 E
• 436 P
• 132 Q
130 self:
• 3 E
• 44 P
• 84 Q
597 successful:
• 6 E
• 446 P
• 145 Q
89 self:
• 0 E
• 29 P
• 60 Q
Admission to tertiary
193 admissions to QECH
18/93
97/525
41/712
44/686
Death at tertiary
2 deaths at QECH
0
2
0
0

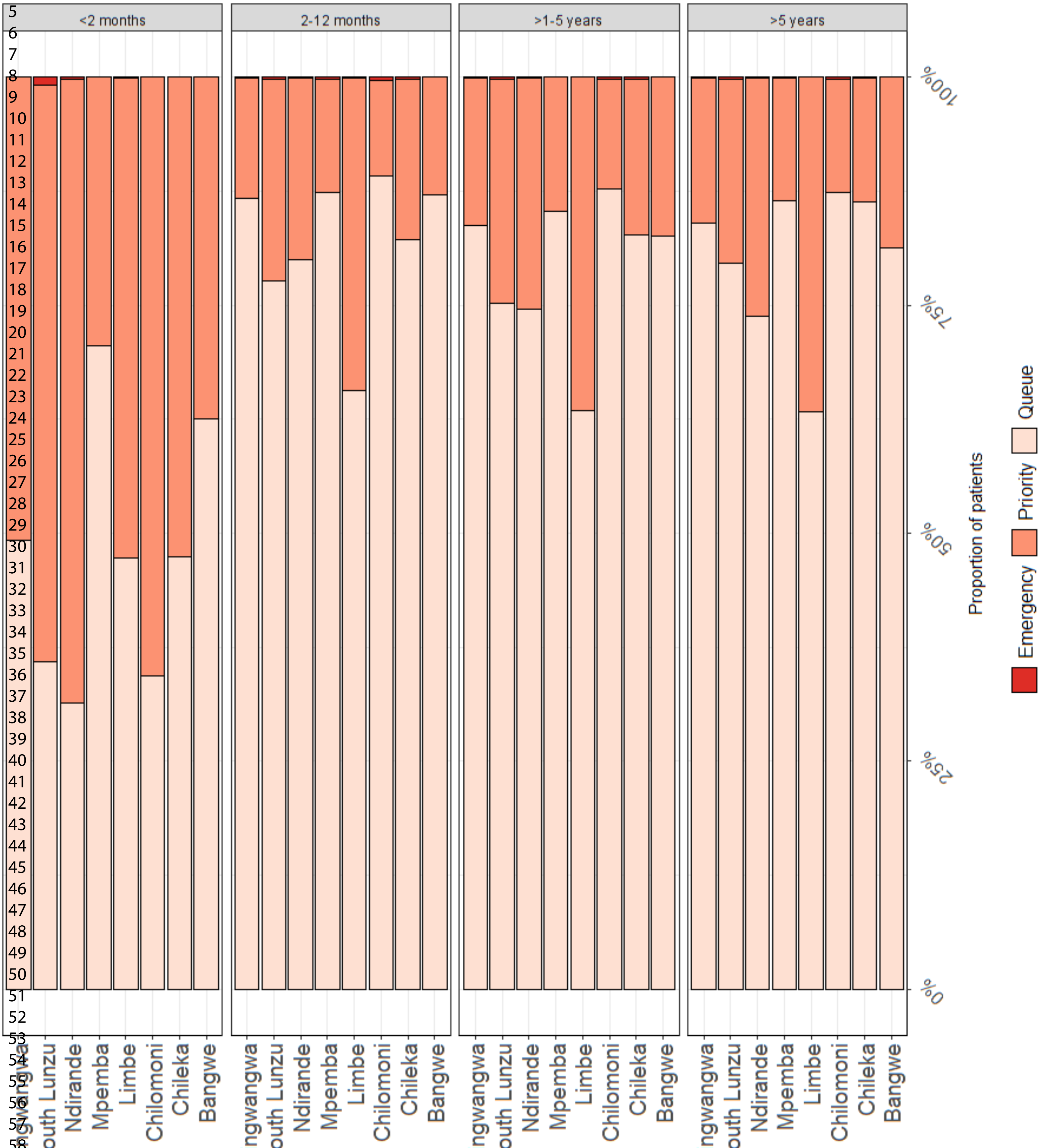

<2 months
2-12 months
>1-5 years
>5 years
Zingwangwa
South Lunzu
Ndirande
Mpemba
Limbe
Chilomoni
Chileka
Bangwe
0%
25%
50%
75%
100%
Proportion of patients
Emergency
Priority
Queue

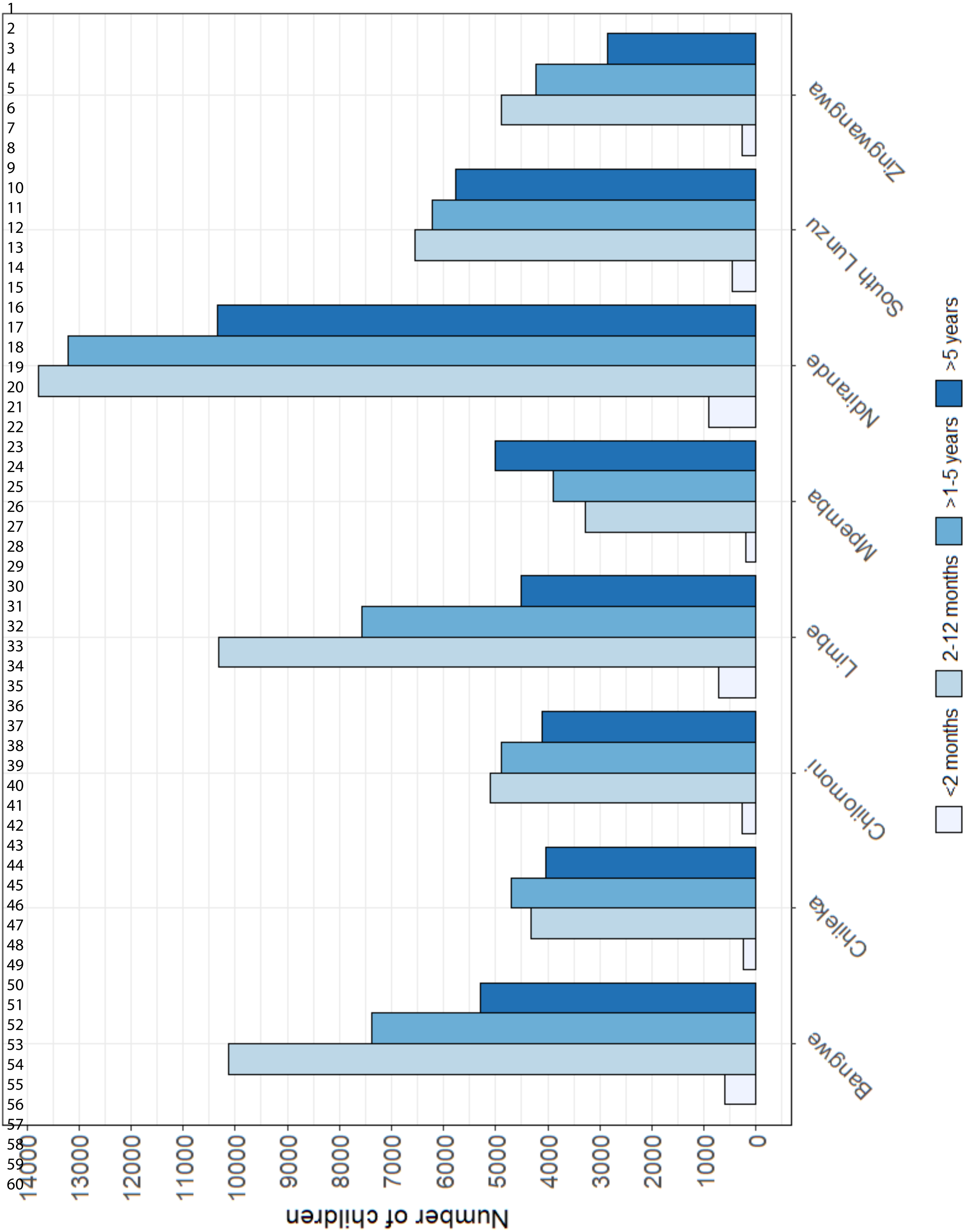
Zingwangwa
South Lunzu
Ndirande
Mpemba
Limbe
Chilomoni
Chileka
Bangwe
<2 months
2-12 months
>1-5 years
>5 years
0
1000
2000
3000
4000
5000
6000
7000
8000
9000
10000
11000
12000
13000
14000
Number of children

Supplementary Table 1. Complete characteristics of children attending primary health centre

Characteristics	Total		<2 months		2-12 months		>1-5 years		>5 years	
	N=155,931	%	N=3,654	%	N=58,337	%	N=52,064	%	N=41,876	%
Male	77,915	50·0	1,889	51·7	29,885	51·2	26,204	50·3	19,937	47·6
Weight, kg (mean | SD)	15·34	8·33	4·09	1·22	8·48	2·39	13·74	3·42	24·59	7·18
**PHC**										
Bangwe	23,389	15·0	604	16·5	10,115	17·3	7,388	14·2	5,282	12·6
Chileka	13,266	8·5	236	6·5	4,309	7·4	4,694	9·0	4,027	9·6
Chilomoni	14,342	9·2	265	7·3	5,097	8·7	4,878	9·4	4,102	9·8
Limbe	23,099	14·8	713	19·5	10,313	17·7	7,564	14·5	4,509	10·8
Mpemba	12,388	7·9	207	5·7	3,277	5·6	3,904	7·5	5,000	11·9
Ndirande	38,243	24·5	912	25·0	13,799	23·6	13,210	25·4	10,342	24·7
South Lunzu	18,990	12·2	457	12·5	6,557	11·2	6,207	11·9	5,769	13·8
Zingwangwa	12,214	7·8	260	7·1	4,890	8·4	4,219	8·1	2,845	6·8
**PHC mHealth triage**										
Emergency	392	0·3	19	0·5	162	0·3	136	0·3	75	0·2
Priority	48,248	30·9	2,974	81·4	15,694	26·9	16,912	32·5	12,668	30·3
Queue	107,291	68·8	661	18·1	42,481	72·8	35,016	67·3	29,133	69·6
**PHC clinician triage**										
Emergency	186	0·1	7	0·2	78	0·1	57	0·1	44	0·1
Priority	33,633	21·6	2,005	54·9	11,370	19·5	11,564	22·2	8,694	20·8
Queue	122,112	78·3	1,642	44·9	46,889	80·4	40,443	77·7	33,138	79·1
**PHC symptoms**										
Fever	515	0·3	23	0·7	231	0·4	166	0·3	93	0·2
Convulsions	100	0·1	1	<0·1	26	<0·1	46	0·1	27	0·1
Diarrhoea	149	0·1	1	<0·1	86	0·1	37	0·1	25	0·1
Vomiting	271	0·2	9	0·2	127	0·2	85	0·2	50	0·1
Cough	340	0·2	24	0·7	201	0·3	74	0·1	41	0·1
Difficulty breathing	309	0·2	26	0·7	199	0·3	65	0·1	19	<0·1
Rash	102	0·1	5	0·1	26	<0·1	35	0·1	36	0·1
Pallor	37	<0·1	1	<0·1	10	<0·1	19	<0·1	7	<0·1
Jaundice	13	<0·1	2	0·1	3	<0·1	5	<0·1	3	<0·1
Oedema	27	<0·1	0	0	10	<0·1	11	<0·1	6	<0·1
Urination problems	13	<0·1	3	0·1	1	<0·1	6	<0·1	3	<0·1
Feeding problems	97	0·1	5	0·1	46	0·1	27	0·1	19	<0·1
Other	1,498	1·0	50	1·4	227	0·4	557	1·1	664	1·6
**PHC laboratory test**	503	0·3	10	0·3	203	0·3	168	0·3	122	0·3
Haemoglobin	46	<0·1	1	<0·1	11	<0·1	20	<0·1	14	<0·1
Glucose	7	<0·1	0	0	1	<0·1	4	<0·1	2	<0·1
MPS	16	<0·1	0	0	3	<0·1	9	<0·1	4	<0·1
MRDT	483	0·3	9	0·2	201	0·3	159	0·3	114	0·3
Urine	4	<0·1	0	0	0	0	3	<0·1	1	<0·1
**PHC outcome**										
Admitted to short stay	181	0·1	3	0·1	67	0·1	62	0·1	49	0·1
Referred after triage	2,487	1·6	109	3·0	562	1·0	846	1·6	970	2·3
Referred after short stay	517[a]	0·3	28	0·8	128	0·2	188	0·4	173	0·4
Total referred from HC	3,004	1·9	137	3·7	690	1·2	1,034	2·0	1,143	2·7
Successful referral[b]	1,644/ 3,004	54·7	72/ 137	52·6	393/ 690	57·0	582/ 1,034	56·3	597/ 1,143	52·2
Self-referral[c]	372	0·2	21	0·6	132	0·2	130	0·2	89	0·2
**QECH A&E**										
Successful referral arrived by ambulance	53/ 1,644	3·2	2/ 137	1·5	24/ 690	3·5	16/ 1,034	1·5	11/ 1,143	1·0
Self-referral arrived by ambulance	22/ 372	5·9	3/ 21	14·3	9/ 132	6·8	5/ 130	3·8	5/ 89	5·6
Total arrived by ambulance	75/ 2,016	3·7	5/ 93	5·4	33/ 525	6·3	21/ 712	4·4	16/ 686	2·3
**QECH symptoms**										

For peer review only

Fever	205/ 2,016	10·2	20/ 93	21·5	111/ 525	21·1	39/ 712	5·5	35/ 686	5·1
Convulsions	32/ 2,016	1·6	1/ 93	1·1	7/ 525	1·3	20/ 712	2·8	4/ 686	0·6
Diarrhoea	49/ 2,016	2·4	2/ 93	2·2	39/ 525	7·4	4/ 712	0·6	4/ 686	0·6
Vomiting	75/ 2,016	3·7	5/ 93	5·4	41/ 525	7·8	13/ 712	1·8	16/ 686	2·3
Cough	159/ 2,016	7·9	15/ 93	16·1	94/ 525	17·9	28/ 712	3·9	22/ 686	2·8
Difficulty breathing	148/ 2,016	7·3	15/ 93	16·1	89/ 525	17·0	25/ 712	3·5	19/ 686	3·2
Rash	7/ 2,016	0·3	0	0	4/ 525	0·8	3/ 712	0·4	0	0
Pallor	25/ 2,016	1·2	0	0	6/ 525	1·1	8/ 712	1·1	11/ 686	1·6
Jaundice	11/ 2,016	0·5	7/ 93	7·5	0	0	3/ 712	0·4	1/ 686	0·1
Oedema	16/ 2,016	0·8	0	0	7/ 525	1·3	3/ 712	0·4	6/ 686	0·9
Urination problems	3/ 2,016	0·1	1/ 93	1·1	0	0	1/ 712	0·1	1/ 686	0·1
Feeding problems	52/ 2,016	2·6	8/ 93	8·6	27/ 525	5·1	6/ 712	0·8	11/ 686	1·6
Other	117/ 2,016	5·8	10/ 93	10·8	42/ 525	8·0	26/ 712	3·7	39/ 686	5·7
**QECH diagnosis**										
Trauma	9/ 2,016	0·4	0	0	2/ 525	0·4	4/ 712	0·6	3/ 686	0·4
Gastroenteritis	35/ 2,016	1·7	2/ 93	2·2	29/ 525	5·5	2/ 712	0·3	2/ 686	0·3
Pneumonia	52/ 2,016	2·6	4/ 93	4·3	37/ 525	7·0	5/ 712	0·7	6/ 686	0·9
Meningitis	6/ 2,016	0·3	0	0	2/ 525	0·4	3/ 712	0·4	1/ 686	0·1
Malnutrition	25/ 2,016	1·2	1/ 93	1·1	16/ 525	3·0	4/ 712	0·6	4/ 686	0·6
Sepsis	16/ 2,016	0·8	13/ 93	14·0	3/ 525	0·6	0	0	0	0
Malaria	41/ 2,016	2·0	0	0	14/ 525	2·7	18/ 712	2·5	9/ 686	1·3
Anaemia	26/ 2,016	1·3	1/ 93	1·1	8/ 525	1·5	7/ 712	0·9	10/ 686	1·5
Other	201/ 2,016	10·0	19/ 93	2-·4	97/ 525	18·5	41/ 712	5·8	44/ 686	6·4
**QECH laboratory test**	193/ 2,016	9·6	18/ 93	19·4	97/ 525	18·5	47/ 712	5·8	36/ 686	5·2
CSF culture	11/ 2,016	0·5	4/ 93	4·3	2/ 525	0·4	3/ 712	0·4	2/ 686	0·3
Blood culture	41/ 2,016	2·0	10/ 93	10·8	15/ 525	2·9	5/ 712	0·7	11/ 686	1·6
Blood glucose	23/ 2,016	1·1	2/ 93	2·2	15/ 525	2·9	3/ 712	0·4	3/ 686	0·4
HIV	148/ 2,016	7·3	12/ 93	12·9	74/ 525	14·1	32/ 712	4·5	30/ 686	4·4
MRDT	90/ 2,016	4·5	7/ 93	7·5	42/ 525	8·0	22/ 712	3·1	19/ 686	2·8
MPS	127/ 2,016	6·3	13/ 93	14·0	66/ 525	12·6	23/ 712	3·2	25/ 686	3·6
PCV	127/ 2,016	6·3	13/ 93	14·0	67/ 525	12·8	22/ 712	3·1	25/ 686	3·6
Other	38/ 2,016	1·9	1/ 93	1·1	13/ 525	2·5	8/ 712	1·1	16/ 686	2·3
**QECH laboratory test result**										
Positive blood culture	5/ 41	12·2	1/ 10	10	0/ 15	0	1/ 5	20	3/ 11	27
Positive CSF culture	11/ 11	100	4/ 4	100	2/ 2	100	3/ 3	100	2/2	100
Positive HIV test	8/ 148	5·4	0/ 12	0	2/ 74	27	0/ 32	0	6/ 30	20
Positive MRDT	17/ 90	18·9	0/ 7	0	5/ 42	12	7/ 22	32	5/ 19	26
Positive MPS	19/ 127	15·0	0/ 13	0	5/ 66	8	7/ 23	30	5/ 25	20
Low blood glucose	5/ 23	21·7	1/ 2	50	2/ 15	14	0/ 3	0	2/ 3	67
PCV (mean | SD)	32·17	8·11	35·62	7·59	32·76	6·11	33·68	8·12	27·44	11·12
**QECH A&E outcome**										
Died	2/ 2290	0·1	0/ 119	0	2/ 663	0·1	0/ 770	0	0/ 738	0

A&E, accident and emergency; CSF, cerebrospinal fluid; HIV, human immunodeficiency virus; mHealth, mobile health; MRDT, malaria rapid diagnostic test; MPS, malaria parasite screen; PCV, packed cell volume; PHC, primary health centre; QECH, Queen Elizabeth Central Hospital; SD, standard deviation

[a]517 cases that were referred to QECH from PHC were still admitted to short stay at the PHC, where they were again referred to QECH.

[b]Referred by PHC and arrived at QECH.

[c]Not referred by PHC but arrived at QECH.

For peer review only

Supplementary Table 2A. For young infants <2 months, emergency and priority signs, referral, and successful referral

	Referred		Not referred		Successfully referred		Not successfully referred	
	N=137	%	N=3,517	%	N=72	%	N=65	%
**Emergency signs**								
**Breathing**								
Obstructed breathing	1	0.7	8	0.2	0	0	1	1.5
Central cyanosis	0	0	1	<0.1	0	0	0	0
Respiratory distress	1	0.7	8	0.2	0	0	1	1.5
**Circulation**								
Cold hands	0	0	45	1.3	0	0	0	0
Capillary refill >3 secs	0	0	1	2.2	0	0	0	0
Weak and fast pulse	0	0	1	2.2	0	0	0	0
**Consciousness**								
Coma	0	0	0	0	0	0	0	0
Convulsions	1	0.7	0	0	0	0	1	1.5
**Dehydration**								
Diarrhoea	4	3.0	195	5.6	1	1.4	3	4.6
Lethargic or unconscious	0	0	3,306	94.0	0	0	0	0
Sunken eyes	0	0	0	0	0	0	0	0
Very slow skin pinch	0	0	0	0	0	0	0	0
**Priority signs**								
Tiny baby	108	78.2	2,616	74.4	60	83.3	49	74.2
Temperature	7	5.1	181	5.1	3	4.2	4	6.1
Trauma	2	1.5	10	0.3	1	1.4	1	1.5
Severe pain	0	0	28	0.8	0	0	0	0
Respiratory distress	0	0	21	0.6	0	0	0	0
Burns	0	0	1	<0.1	0	0	0	0

Supplementary Table 2B. For infants 2-12 months, emergency and priority signs, referral, and successful referral

	Referred		Not referred		Successfully referred		Not successfully referred	
	N=690	%	N=57,647	%	N=393	%	N=297	%
**Emergency signs**								
**Breathing**								
Obstructed breathing	23	3.3	49	<0.1	17	4.3	6	2.0
Central cyanosis	0	0	0	0	0	0	0	0

For peer review only - http://bmjopen.bmj.com/site/about/guidelines.xhtml

For peer review only

Respiratory distress	25	3.6	44	<0.1	16	4.1	9	3.0
**Circulation**								
Cold hands	3	0.4	621	1.1	1	0.3	2	0.7
Capillary refill >3 secs	0	0	5	<0.1	0	0	0	0
Weak and fast pulse	0	0	8	<0.1	0	0	0	0
**Consciousness**								
Coma	0	0	1	<0.1	0	0	0	0
Convulsions	7	1.1	9	<0.1	5	1.3	2	0.7
**Dehydration**								
Diarrhoea	89	12.9	13,828	24.0	62	15.8	27	9.1
Lethargic or unconscious	3	0.4	66	0.1	2	0.5	1	0.3
Sunken eyes	15	2.2	182	0.3	13	3.3	2	0.7
Very slow skin pinch	1	0.1	34	0.1	1	0.3	0	0
**Priority signs**								
Tiny baby	29	4.2	1,152	2.0	16	4.1	13	4.4
Temperature	133	19.3	12,383	21.5	80	20.4	53	17.8
Trauma	94	13.6	580	1.0	61	15.5	33	11.1
Severe pallor	1	0.1	32	<0.1	0	0	1	0.3
Poisoning	3	0.4	7	<0.1	3	0.8	0	0
Severe pain	33	4.8	391	0.7	16	4.1	17	5.7
Respiratory distress	31	4.5	533	0.9	22	5.6	9	3.0
Restless/lethargic	4	0.6	81	0.1	4	1.0	0	0
Urgent referral	4	0.6	8	<0.1	3	0.8	1	0.3
Malnutrition	7	1.0	26	<0.1	6	1.5	1	0.3
Oedema	2	0.3	18	<0.1	1	0.3	1	0.3
Burns	17	2.5	125	0.2	3	0.8	14	4.7

For peer review only

Supplementary Table 2C. For children >1-5 years, emergency and priority signs, referral, and successful referral

	Referred		Not referred		Successfully referred		Not successfully referred	
	N=1,034	%	N=51,030	%	N=582	%	N=452	%
**Emergency signs**								
**Breathing**								
Obstructed breathing	4	0.4	45	<0.1	2	0.3	2	0.4
Central cyanosis	1	<0.1	3	<0.1	0	0	1	0.2
Respiratory distress	9	0.9	30	<0.1	5	0.9	4	0.9
**Circulation**								
Cold hands	8	0.8	548	1.1	4	0.7	4	0.9
Capillary refill >3 secs	0	0	2	<0.1	0	0	0	0
Weak and fast pulse	1	0.1	11	<0.1	1	0.2	0	0
**Consciousness**								
Coma	3	0.3	2	<0.1	1	0.2	2	0.4
Convulsions	16	1.6	20	<0.1	10	1.7	6	1.3
**Dehydration**								

For peer review only - http://bmjopen.bmj.com/site/about/guidelines.xhtml

Diarrhoea	37	3.7	8,298	16.3	22	3.8	15	3.3
Lethargic or unconscious	3	0.3	52	0.1	0	0	3	0.7
Sunken eyes	1	0.1	98	0.2	0	0	1	0.2
Very slow skin pinch	0	0	26	0.1	0	0	0	0
**Priority signs**								
Tiny baby	0	0	88	0.2	0	0	0	0
Temperature	89	8.6	13,826	27.1	53	9.1	36	8.0
Trauma	401	38.8	1,029	2.0	242	41.6	159	35.2
Severe pallor	2	0.2	209	0.4	0	0	2	0.4
Poisoning	1	<0.1	12	<0.1	0	0	1	0.2
Severe pain	67	6.5	631	1.2	36	6.2	31	6.9
Respiratory distress	16	1.5	244	0.5	13	2.2	3	0.7
Restless/lethargic	1	<0.1	44	<0.1	0	0	1	0.2
Urgent referral	3	0.3	8	<0.1	0	0	3	0.7
Malnutrition	5	0.5	15	<0.1	0	0	5	1.1
Oedema	7	0.7	28	<0.1	2	0.3	5	1.1
Burns	38	3.7	148	0.3	16	2.7	22	4.9

Supplementary Table 2D. For children >5 years, emergency and priority signs, referral, and successful referral

	Referred		Not referred		Successfully referred		Not successfully referred	
	N=1,143	%	N=40,733	%	N=597	%	N=546	%
**Emergency signs**								
**Breathing**								
Obstructed breathing	1	<0.1	31	<0.1	0	0	1	0.2
Central cyanosis	0	0	0	0	0	0	0	0
Respiratory distress	1	<0.1	13	<0.1	1	0.2	0	0
**Circulation**								
Cold hands	2	0.2	367	0.9	1	0.2	1	0.2
Capillary refill >3 secs	0	0	4	1.1	0	0	0	0
Weak and fast pulse	1	<0.1	10	2.7	1	0.2	0	0
**Consciousness**								
Coma	3	0.3	8	<0.1	2	0.3	1	0.2
Convulsions	4	0.4	14	<0.1	3	0.5	1	0.2
**Dehydration**								
Diarrhoea	30	2.6	5,040	12.4	16	2.7	14	2.6
Lethargic or unconscious	0	0	18	0.4	0	0	0	0
Sunken eyes	0	0	46	0.9	0	0	0	0
Very slow skin pinch	0	0	9	0.2	0	0	0	0
**Priority signs**								
Tiny baby	0	0	36	<0.1	0	0	0	0
Temperature	84	7.3	9,928	24.3	46	7.7	38	7.0
Trauma	514	45.0	920	2.3	294	49.2	220	40.3
Severe pallor	0	0	55	0.1	0	0	0	0

For peer review only

Poisoning	2	0.2	11	<0.1	1	0.2	1	0.2
Severe pain	105	9.2	750	1.8	58	9.7	47	8.6
Respiratory distress	2	0.2	128	0.3	2	0.3	0	0
Restless/lethargic	1	<0.1	29	<0.1	0	0	1	0.2
Urgent referral	1	<0.1	7	<0.1	1	0.2	0	0
Malnutrition	0	0	6	<0.1	0	0	0	0
Oedema	4	0.3	8	<0.1	2	0.3	2	0.4
Burns	10	0.9	67	0.2	2	0.3	8	1.5

For peer review only

Supplementary Table 3. Referrals, and successful referrals by triage outcome and age group

Age group	Triage outcome	Referred		Not referred		Successfully referred		Not successfully referred	
		N	%	N	%	N	%	N	%
Overall	Emergency	97/ 3,004	3.2	89/ 152,927	0.1	52/ 1,644	3.2	45/ 1,361	3.3
	Priority	2,126/ 3,004	70.8	31,507/ 152,927	20.6	1,216/ 1,644	74.0	911/ 1,361	66.9
	Queue	781/ 3,002	26.0	121,331/ 152,927	79.3	376/ 1,644	22.9	405/ 1,361	29.8
<2 months	Emergency	2/ 137	1.5	5/ 3,517	0.1	0/ 72	0	2/ 65	3.0
	Priority	126/ 137	92.0	1,879	53.4	67/ 72	93.1	59/ 65	90.8
	Queue	9/ 137	6.6	1,633	46.4	5/ 72	6.9	4/ 65	6.2
2-12 months	Emergency	55/ 690	8.0	23/ 57,647	<0.1	32/ 393	8.1	23/ 297	7.7
	Priority	452/ 690	65.5	10,918/ 57,647	18.9	267/ 393	67.9	185/ 297	62.3
	Queue	183/ 690	26.5	46,706/ 57,647	81.0	94/ 393	23.9	89/ 297	30.0
>1-5 years	Emergency	28/ 1,034	2.7	29/ 51,030	0.1	14/ 582	2.4	14/ 452	3.1
	Priority	737/ 1,034	71.3	10,827/ 51,030	21.2	436/ 582	74.9	301/ 452	66.6
	Queue	269/ 1,034	26.0	40,174/ 51,030	78.7	132/ 582	22.7	137/ 452	30.3
>5 years	Emergency	12/ 1,143	1.0	32/ 40,733	0.1	6/ 597	1.0	6/ 546	1.1
	Priority	811/ 1,143	71.0	7,883/ 40,733	19.4	446/ 597	74.7	365/ 546	66.8
	Queue	320/ 1,143	28.0	32,818/ 40,733	80.6	145/ 597	24.3	175/ 546	32.1

STROBE Statement—checklist of items that should be included in reports of observational studies

	Item No.	Recommendation	Page No.	Relevant text from manuscript
**Title and abstract**	1	(*a*) Indicate the study's design with a commonly used term in the title or the abstract	1	
		(*b*) Provide in the abstract an informative and balanced summary of what was done and what was found	2	
**Introduction**				
Background/rationale	2	Explain the scientific background and rationale for the investigation being reported	4	
Objectives	3	State specific objectives, including any prespecified hypotheses	4-5	
**Methods**				
Study design	4	Present key elements of study design early in the paper	5	
Setting	5	Describe the setting, locations, and relevant dates, including periods of recruitment, exposure, follow-up, and data collection	5-6	
Participants	6	(*a*) *Cohort study*—Give the eligibility criteria, and the sources and methods of selection of participants. Describe methods of follow-up<br>*Case-control study*—Give the eligibility criteria, and the sources and methods of case ascertainment and control selection. Give the rationale for the choice of cases and controls<br>*Cross-sectional study*—Give the eligibility criteria, and the sources and methods of selection of participants	6	
		(*b*) *Cohort study*—For matched studies, give matching criteria and number of exposed and unexposed<br>*Case-control study*—For matched studies, give matching criteria and the number of controls per case	NA	
Variables	7	Clearly define all outcomes, exposures, predictors, potential confounders, and effect modifiers. Give diagnostic criteria, if applicable	6	
Data sources/ measurement	8*	For each variable of interest, give sources of data and details of methods of assessment (measurement). Describe comparability of assessment methods if there is more than one group	*6*	
Bias	9	Describe any efforts to address potential sources of bias	6-7	
Study size	10	Explain how the study size was arrived at	7	

Continued on next page

For peer review only - http://bmjopen.bmj.com/site/about/guidelines.xhtml

Quantitative variables	11	Explain how quantitative variables were handled in the analyses. If applicable, describe which groupings were chosen and why	6-7
Statistical methods	12	(*a*) Describe all statistical methods, including those used to control for confounding	6-7
		(*b*) Describe any methods used to examine subgroups and interactions	6-7
		(*c*) Explain how missing data were addressed	6-7
		(*d*) *Cohort study*—If applicable, explain how loss to follow-up was addressed<br>*Case-control study*—If applicable, explain how matching of cases and controls was addressed<br>*Cross-sectional study*—If applicable, describe analytical methods taking account of sampling strategy	6-7
		(*e*) Describe any sensitivity analyses	NA
**Results**			
Participants	13*	(a) Report numbers of individuals at each stage of study—eg numbers potentially eligible, examined for eligibility, confirmed eligible, included in the study, completing follow-up, and analysed	7
		(b) Give reasons for non-participation at each stage	NA
		(c) Consider use of a flow diagram	NA
Descriptive data	14*	(a) Give characteristics of study participants (eg demographic, clinical, social) and information on exposures and potential confounders	7
		(b) Indicate number of participants with missing data for each variable of interest	7
		(c) *Cohort study*—Summarise follow-up time (eg, average and total amount)	NA
Outcome data	15*	*Cohort study*—Report numbers of outcome events or summary measures over time	*NA*
		Case-control study—Report numbers in each exposure category, or summary measures of exposure	*NA*
		Cross-sectional study—Report numbers of outcome events or summary measures	*7*
Main results	16	(*a*) Give unadjusted estimates and, if applicable, confounder-adjusted estimates and their precision (eg, 95% confidence interval). Make clear which confounders were adjusted for and why they were included	7-9
		(*b*) Report category boundaries when continuous variables were categorized	7-9
		(*c*) If relevant, consider translating estimates of relative risk into absolute risk for a meaningful time period	NA

Continued on next page

For peer review only - http://bmjopen.bmj.com/site/about/guidelines.xhtml

Other analyses	17	Report other analyses done—eg analyses of subgroups and interactions, and sensitivity analyses	NA
**Discussion**			
Key results	18	Summarise key results with reference to study objectives	9
Limitations	19	Discuss limitations of the study, taking into account sources of potential bias or imprecision. Discuss both direction and magnitude of any potential bias	12
Interpretation	20	Give a cautious overall interpretation of results considering objectives, limitations, multiplicity of analyses, results from similar studies, and other relevant evidence	9-13
Generalisability	21	Discuss the generalisability (external validity) of the study results	12-13
**Other information**			
Funding	22	Give the source of funding and the role of the funders for the present study and, if applicable, for the original study on which the present article is based	14

*Give information separately for cases and controls in case-control studies and, if applicable, for exposed and unexposed groups in cohort and cross-sectional studies.

Note: An Explanation and Elaboration article discusses each checklist item and gives methodological background and published examples of transparent reporting. The STROBE checklist is best used in conjunction with this article (freely available on the Web sites of PLoS Medicine at http://www.plosmedicine.org/, Annals of Internal Medicine at http://www.annals.org/, and Epidemiology at http://www.epidem.com/). Information on the STROBE Initiative is available at www.strobe-statement.org.

For peer review only

BMJ Open

Child acute illness presentation and referrals at primary health clinics in Malawi: a secondary analysis of ASPIRE

Journal:	*BMJ Open*
Manuscript ID	bmjopen-2023-079589.R2
Article Type:	Original research
Date Submitted by the Author:	28-Mar-2024

Complete List of Authors:	Iroh Tam, Pui-Ying ; Malawi-Liverpool-Wellcome Trust Clinical Research Programme, ; Liverpool School of Tropical Medicine,<br>Twabi, Hussein; Malawi-Liverpool-Wellcome Trust Clinical Research Programme<br>Gondwe, Mtisunge; Malawi-Liverpool-Wellcome Trust Clinical Research Programme<br>O'Byrne, Thomasena; Malawi-Liverpool-Wellcome Trust Clinical Research Programme<br>Lufesi, Norman; Government of Malawi Ministry of Health, Acute Respiratory Illness Unit<br>Desmond, Nicola; Liverpool School of Tropical Medicine, International Public Health

**Primary Subject Heading**:	Paediatrics
Secondary Subject Heading:	Global health, Public health
Keywords:	PAEDIATRICS, EPIDEMIOLOGY, International health services < HEALTH SERVICES ADMINISTRATION & MANAGEMENT, Community child health < PAEDIATRICS, Primary Care < Primary Health Care, PUBLIC HEALTH

SCHOLARONE™
Manuscripts

I, the Submitting Author has the right to grant and does grant on behalf of all authors of the Work (as defined in the below author licence), an exclusive licence and/or a non-exclusive licence for contributions from authors who are: i) UK Crown employees; ii) where BMJ has agreed a CC-BY licence shall apply, and/or iii) in accordance with the terms applicable for US Federal Government officers or employees acting as part of their official duties; on a worldwide, perpetual, irrevocable, royalty-free basis to BMJ Publishing Group Ltd ("BMJ") its licensees and where the relevant Journal is co-owned by BMJ to the co-owners of the Journal, to publish the Work in this journal and any other BMJ products and to exploit all rights, as set out in our licence.

The Submitting Author accepts and understands that any supply made under these terms is made by BMJ to the Submitting Author unless you are acting as an employee on behalf of your employer or a postgraduate student of an affiliated institution which is paying any applicable article publishing charge ("APC") for Open Access articles. Where the Submitting Author wishes to make the Work available on an Open Access basis (and intends to pay the relevant APC), the terms of reuse of such Open Access shall be governed by a Creative Commons licence – details of these licences and which Creative Commons licence will apply to this Work are set out in our licence referred to above.

Other than as permitted in any relevant BMJ Author's Self Archiving Policies, I confirm this Work has not been accepted for publication elsewhere, is not being considered for publication elsewhere and does not duplicate material already published. I confirm all authors consent to publication of this Work and authorise the granting of this licence.

For peer review only

Child acute illness presentation and referrals at primary health clinics in Malawi: a secondary analysis of ASPIRE

Pui-Ying Iroh Tam, DMed,[1,2,3*] Hussein H Twabi, MSc,[1,3,5] Mtisunge Gondwe, PhD,[1,3] Thomasena O'Byrne, PhD,[1,2] Norman Lufesi, MPhil,[4] Nicola Desmond, PhD[2]

*Corresponding author: irohtam@mlw.mw

1. Malawi-Liverpool-Wellcome Research Programme, Blantyre, Malawi
2. Liverpool School of Tropical Medicine, Liverpool, United Kingdom
3. Kamuzu University of Health Sciences, Blantyre, Malawi
4. Ministry of Health Malawi, Lilongwe, Malawi
5. University of Liverpool, Liverpool, United Kingdom

Word count: 2,599

For peer review only

Objectives: We aimed to assess the prevalence, presentation, and referral patterns of children with acute illness attending primary health centres (PHCs) in a low-resource setting.

Design, setting, and participants: We conducted a secondary analysis of ASPIRE. Children presenting at eight PHCs in urban Blantyre district in southern Malawi with both recorded clinician and mHealth (non-clinician) triage data were included, and patient records from different data collection points along the patient healthcare seeking pathway were consolidated and analysed.

Results: Between April 2017 to September 2018, a total of 204,924 children were triaged, of whom 155,931 had both recorded clinician and mHealth triage data. The most common presenting symptoms at PHCs were fever (0·3%), cough (0·2%), and difficulty breathing (0·2%). The most common signs associated with referral for under-5 children were trauma (26·7%) and temperature (7·4%). The proportion of emergency and priority clinician triage were highest among young infants <2 months ((0·2%) and 81·4%, respectively). Of the 3,004 referrals (1·9%), 1,644 successfully reached the referral facility (54·7%). Additionally, 372 children were sent home from PHC who subsequently self-referred to the referral facility (18·7%).

Conclusions: Fever and respiratory symptoms were the most common presenting symptoms, and trauma was the most common reason for referral. Rates of referral were low, and of successful referral were moderate. Self-referrals constituted a substantial proportion of attendance at the referral facility. Reducing gaps in care and addressing dropouts as well as self-referrals along the referral pathway could improve child health outcomes.

Word count: 243

For peer review only

Strengths and limitations of this study:

- This study prospectively and systematically quantifies child acute illness presentation and referral patterns in an urban district setting in sub-Saharan Africa.
- Analyses a mHealth triage and surveillance dataset of 155,931 children.
- Leverages a mHealth patient surveillance system that tracked those successful referred to the referral facility, as well as patients that presented to primary health centres, were sent home, and then later self-referred to the referral facility.
- Limitations were those inherent in a mHealth surveillance system reliant on existing healthcare workers rather than research-trained staff, including issues of data completeness.

Keywords: primary level; child; global health

For peer review only

Introduction

The leading global cause of mortality in children under five year is preventable infections, namely pneumonia and diarrhoea,[1] and WHO developed the Integrated Management of Childhood Illness (IMCI)[2] to reduce mortality due to these causes. The guidelines provided by IMCI, the integrated Community Case Management (iCCM)[3] and Emergency Triage Assessment and Treatment (ETAT)[4] are part of a comprehensive effort to improve the quality of care of ill children at different levels of the health system in low-resource settings, and to promote access and early initiation of first-line treatment or referral of severe cases to inpatient facilities, and to reduce mortality.[5-7]

Referral is a critical component of the IMCI strategy, intended to recognise children with severe conditions and ensure appropriate management and optimise quality care at the proper level of the health system.[8, 9] In Malawi in 2013-2014, only 3.5% of hospitals were equipped to provide basic paediatric emergency care in a national facility census.[10] In low-resource settings in sub-Saharan Africa prior to the implementation of IMCI, <2% of children presenting at primary level facilities were referred;[8, 11] after the introduction of IMCI, referral rates for children were 6-20%.[12] Challenges with the referral pathway range from parental education[9, 13] to communication,[9] transport,[9, 14-16] and cost.[14, 16] Therefore, for children who have been referred, successful referral (i.e. arrival at the referral facility) has been documented to be as low as 28%.[16]

While social factors affect presentation at and referrals from PHCs, the clinical features, healthcare utilization, and referral patterns in this setting have been poorly quantified. Elucidating these features can help identify where to allocate scarce resources, and reduce

For peer review only - http://bmjopen.bmj.com/site/about/guidelines.xhtml

inequities in access. We sought to determine the most common features of acute illness among children presenting to primary health centres (PHCs) in an urban, low-resource setting within reasonable proximity to a referral facility, and what features are related to referral and successful referral by age group.

Methods

Study design, setting, and participants

The current study is a secondary analysis of data from the Achieving Sustainable Primary Improvement and Engagement in health (ASPIRE) project. At the primary level in Malawi, clinical care is provided by clinically trained health workers (clinicians, nurses, medical assistants) and non-clinically trained health workers (health surveillance assistants, security guards, and other health centre staff). Human resource shortages mean that non-clinically trained health workers are often required to fill the gaps in service provision.

ASPIRE implemented a mobile health (mHealth) triage algorithm based on Emergency Triage and Treatment (ETAT) for primary-level care. For ASPIRE, non-clinical health workers positioned at both PHCs and the referral facility (Queen Elizabeth Central Hospital – QECH) were trained and used the mHealth algorithm to document the characteristics of all patients up to 14 years of age, including their symptoms, investigations, triage outcomes, and referral statuses as they presented at these sites in urban Blantyre. As part of the same PHC visit, data were first collected by the non-clinical health worker using the mHealth application (mHealth triage), and then separately by the clinical health worker (clinician triage).

For peer review only - http://bmjopen.bmj.com/site/about/guidelines.xhtml

In 2014, the district of Blantyre had a population of 1·9 million,[17] about half of whom were under 15 years of age, with the majority residing in peri-urban townships. QECH is a tertiary-level hospital and referral facility in the southern region of the country; patients are thus expected to first be seen in a PHC or district hospital before presentation to QECH.

Public involvement

Between 2013 and 2017, the ASPIRE mHealth primary ETAT was developed and tested, with the involvement of district and primary health level stakeholders and national policymakers, and then implemented from 2017 to 2018 across eight PHCs in Blantyre district in southern Malawi. Details of the study have been published previously.[18, 19]

Procedures

An mHealth patient surveillance system was established to monitor patients from initial presentation at the PHC through referral to QECH and final outcome. Participants were entered into the surveillance system as they entered the PHC and assigned a unique identification number (ID), and the mHealth triage outcome following implementation of the algorithm would be recorded by the healthcare worker, together with the facility name, sex and age of the patient. After review by a clinician, the clinician triage and consultation outcome (sent home, admitted for short stay, or referred) were documented, as well as any laboratory tests that were conducted. For all referrals, patients were tracked through a standard referral stamp to arrival at QECH. At QECH, presenting features, laboratory tests conducted, final diagnosis, and outcome (alive, died, absconded) were documented. A referral was defined as any patient referred from the PHC (either clinic or short stay); successful referral was defined as any patient referred from PHC who arrived at QECH; and

For peer review only

self-referral was defined as any patient sent home from PHC who subsequently arrived at QECH.

Ethical approval for this study was obtained from the College of Medicine Research Ethics Committee (P·09/16/2021). Since the only patient identifier collected was age, patient-level consent was waived. Facility-level consent was obtained for data collection through the mHealth tool. All data were analysed at aggregate level and anonymised before analysis.

Statistical analysis

Prior to analysis, we removed records with invalid patient IDs and duplicated patient IDs that could not be matched unambiguously between data collection points. Records from the different data collection points were matched by patient IDs to allow for patient journey monitoring. Data for the different collection points were extracted as csv files from the database, then analysed in RStudio version 446 (2023·03·1) using R version 4·3·0 (2023-04-21). Descriptive statistics were used to describe the data. Continuous data were described using means and standard deviations (SD) when normally distributed, and medians and interquartile ranges (IQR) when non-normal. Categorical data was described using proportions.

Results

Between April 2017 to September 2018, a total of 204,924 children attended eight PHCs in urban Blantyre (Figures 1 and Supplementary Figure 1), and 155,931 had complete clinician and mHealth triage outcome data (Table 1 and Supplementary Table 1). Of these, 3,654 (2·3%) were <2 months, 58,337 (37·4%) were 2-12 months, 52,064 (33·4%) were >1-5 years, and 41,876 (26·9%) were >5 years.

For peer review only - http://bmjopen.bmj.com/site/about/guidelines.xhtml

At PHCs, 392 (0·3%) received a mHealth triage of Emergency and 48,248 (30·9%) of Priority, and 186 received a clinician triage of Emergency (0·1%) and 33,633 (21·6%) of Priority (Figure 2). The proportion of emergency and priority clinician triage were highest among young infants <2 months (7 (0·2%) and 2,974 (81·4%) respectively).

At PHCs, the most common documented presenting symptoms were fever (515; 0·3%), cough (340; 0·2%), and difficulty breathing (309; 0·2%). The most commonly performed test was the malaria rapid diagnostic test (MRDT, 483; 0·3%).

There were a total of 3,004 referrals (1·9%) to QECH. Within age groups, 3·7% of young infants <2 months presenting were referred, compared to 1·2% of 2-12 months, 2·0% of >1-5 years, and 2·7% of >5 years (Supplementary Table 2). Infants 2-12 months comprised the largest proportion of Emergency triage that were referred (8·0%; Supplementary Table 3). Of these referrals, 1,644 (54·7%) were successful. For children ≤5 years (Table 2), the most common emergency signs related to referral were diarrhoea (130; 7·0%), respiratory distress (35; 1·9%) and obstructed breathing (28; 1·5%), and the most common priority signs were trauma (497; 26·7%), temperature (229; 12·3%), and tiny baby (137; 7·4%); these were also the most common emergency and priority signs related to a successful referral.

At QECH, the most commonly documented diagnoses overall were pneumonia (52/2,016; 2·6%) and gastroenteritis (35/2,016; 1·7%). The most commonly performed tests at QECH were the HIV test (148/2,016; 7·3%), malaria parasite screen, and packed cell volume (127/2,016; 6·3% each).

For peer review only - http://bmjopen.bmj.com/site/about/guidelines.xhtml

There were 372 self-referrals, the majority of whom were >1-5 years (Table 3). Of these, 1 (1·3%) received a clinician triage of Emergency, 145 (39·0%) Priority, and 222 (59·7%) Queue. The most common symptoms at PHC among those self-referred were temperature (90; 24·2%) and diarrhoea (54; 14·5%), and the most common diagnoses at QECH was pneumonia (16; 4·3%), malaria (15; 4·0%), and gastroenteritis (14; 3·8%).

Of those successfully referred, 53 arrived by ambulance (3·2%), while 22 who self-referred also arrived by ambulance (5·9%) – this can occur in a setting where an ambulance can provide a dual function to provide local transport in the area.

Forty-one blood and 11 CSF cultures were collected at QECH and 16 were positive (5 blood and 11 CSF). Of the five positive blood cultures, three were triaged as Priority and referred, and two triaged as Queue, sent home, and subsequently self-referred (40%). Of the 11 positive CSF cultures, one was triaged as Emergency and referred, six triaged as Priority and three referred, and five triaged as Queue and two referred; the remainder (45%) were sent home and then self-referred to QECH. Seven of 16 positive cultures (44%) were among children 2 months of age or less.

Two infants 2 months of age died, both at QECH. One was a female that received a clinician triage of Queue at the PHC and was sent home, then self-referred to QECH. At QECH, she was noted to have fever, cough, difficulty breathing, and 'other' problem. CSF culture, blood culture, HIV, MRDT, and other labs were collected, and the child was given a diagnosis of 'other.' The other was a male who received a clinician triage of Priority and was sent home, then self-referred to QECH, where he was noted to have oedema and 'other' problem, and was given a diagnosis of 'other.' CSF culture, blood culture, HIV, MRDT, glucose, and

For peer review only

'other' labs were collected. CSF cultures were positive for both; however, as the PHC patient identifiers did not link up with the microbiology database, further information was not retrieved on the cultures.

Discussion

This study is, to our knowledge, the largest description of child acute illness presentation at primary level clinics and the first to prospectively and comprehensively describe clinical features, and quantify healthcare utilisation and referral patterns in sub-Saharan Africa. We documented over 155,000 children attending PHCs in urban Blantyre, and noted a referral rate of <2%. Though infants 2-12 months had the highest absolute numbers of attendance, young infants <2 months accounted for the largest proportion of emergency and priority signs, and the highest proportion of referrals, with fever and respiratory symptoms being the most common symptoms.

Comprehensive collection of triage at PHCs and prospective tracking of referrals to successful arrival at the referral hospital in a low-resource setting has not been documented previously. Studies of referral patterns in Uganda, Tanzania, Democratic Republic of the Congo, and Nigeria have been based either on retrospective review of records, interviews, focus group discussions, or follow-up visits, and documented successful referral rates ranging from 0·6% to 67%.[8, 16, 20] The largest prospective evaluation of successful referral involved prereferral rectal artesunate among 6,400 children with suspected severe malaria in the Democratic Republic of the Congo, Nigeria, and Uganda, and determined referral completion with a 28-day follow-up visit.

For peer review only - http://bmjopen.bmj.com/site/about/guidelines.xhtml

This study is the first to report successful referrals that have been quantified in a systematic way. We used an mHealth patient surveillance system that allowed us not only to track successful referrals to QECH, but also to identify patients that had been seen in PHCs, sent home, and then self-referred to QECH. Self-referrals made up 18·7% of overall referrals, and to our knowledge is the first time this has been quantified prospectively. Self-referral patterns has been described in other sub-Saharan African settings but those studies have focused on bypassing PHCs entirely,[21-23] rather than self-referral after being sent home from PHC.

We found that young infants were not the largest proportion of attendees at PHCs, but they did account for the largest proportion of emergency and priority signs, and referrals. This supports findings from other studies of sub-Saharan Africa where, compared to those 2-59 months, a higher proportion of young infants <2 months were referred from first-level health facilities in Uganda, Tanzania, and Niger.[12] A systematic review of community-to-facility neonatal referral rates in Africa reported high completion rates ranging from 74-93%.[24] Given that neonatal mortality remains high despite gains made by the Sustainable Development Goals, this highlights the importance of the health system referral pathway to support the care of the young infant age group, and suggests that allocation of resources should target young infants as a vulnerable population.

For a healthcare worker applying IMCI guidelines, IMCI symptom referral criteria has a demonstrated sensitivity of 42-74% and specificity of 93-99%.[25] In our study, the most common symptoms at PHC were fever followed by respiratory symptoms, which corresponds with the commonest diagnoses at the referral hospital of pneumonia and malaria. Malaria was investigated for the most frequently at PHCs and diagnosed commonly at tertiary level for children greater than two months of age. This reflects the continued importance of infectious

For peer review only - http://bmjopen.bmj.com/site/about/guidelines.xhtml

diseases on child morbidity and mortality. Trauma accounted for a sizeable proportion of cases, becoming the predominant cause of referral after the first year of life, highlighting the role and burden of injury in child health.[26]

We collected data on triage, since this has been shown to reduce inpatient mortality by up to two-thirds in low-resource settings.[7] Notably, 59·8% (85/142) of Emergency, 5·3% (1,315/24,939) Priority, and 0·5% (461/88,974) Queue triage cases were referred, which would suggest that there are opportunities to strengthen the IMCI, ETAT, and referral training of healthcare workers. Key health system constraints lead to poor quality paediatric care,[27] and prior assessment of inpatient paediatric care in first-referral level hospitals in Kenya showed that case management guidelines are often not in line with national or international guidelines,[28] which may explain why there were lower than expected rates of referral. It is noteworthy that the two deaths that occurred were both in children 2-12 months of age that had been triaged at PHC, one as Priority and the other as Queue, and then sent home before self-referring to QECH. Given that the Malawi under-5 mortality rate in 2017 was 51 per 1,000 live births, the documentation of only two deaths in this surveillance is low, and may reflect the limitations of the surveillance platform used, as well as prior observations that over half of child deaths were observed to occur at home, and that cases reaching the PHC and hospital may represent only the tip of the iceberg.[8]

This study has limitations. As part of the implementation work around the mHealth surveillance system, existing healthcare workers based at PHCs were trained to collected data as part of their routine duties, but were not research staff trained at the level to conduct a clinical trial. Therefore, the pragmatic approach resulted in issues of data completeness, and did not allow us to interrogate the data more closely. The referral pathway relied on tracking

For peer review only - http://bmjopen.bmj.com/site/about/guidelines.xhtml

patients through a standard referral stamp; therefore, patients who bypassed the PHC triage system due to acute illness or injury, or who misplaced this stamp would therefore not be captured at the referral hospital. The lack of data completeness may therefore affect the interpretation of results by leading to an underestimation of cases and successful referrals. The mHealth surveillance system was based on patient identifiers that were separate from the QECH system, and therefore we were unable to retrieve the results of investigations to fully chronicle the patient journey and cross-check results.

Nevertheless, this study is one of the first to prospectively and systematically quantify child acute illness presentation, healthcare utilisation, and referral patterns in an urban district setting in a low-resource setting. A strong health system providing quality health service requires an effective referral pathway, to promote timely access, judicious allocation of human and physical resources, cost-effective care, and to optimise clinical outcomes. Our study provides quantitative data that allows us to identify gaps in care, which can form the basis to strengthen the referral pathway through regular monitoring, evaluation, and feedback to improve child health outcomes.

For peer review only - http://bmjopen.bmj.com/site/about/guidelines.xhtml

Figure legend

Figure 1. ASPIRE flow diagram of triage and referral from primary health centre to tertiary facility, by age group

Figure 2. Proportion triaged as Emergency, Priority, and Queue, by age group and by facility

Table 1. Characteristics of children attending primary health centre

Table 2. Characteristics of triage outcome and emergency and priority signs with referral for children attending primary health centre for children ≤5 years

Table 3. Characteristics of children discharged from the primary health centre with subsequent self-referral to hospital

Appendix

Supplementary Figure 1. Attendance at primary health centre in Blantyre district, by age group

Supplementary Table 1. Complete characteristics of children attending primary health centre

Supplementary Table 2. Emergency and priority signs, triage outcome, referral, and successful referral for A) <2 months; B) 2-12 months; C) >1-5 years; D) >5 years

Supplementary Table 3. Referrals, and successful referrals by triage outcome and age group

Acknowledgements

We thank Marc Henrion for assistance with the ASPIRE dataset and statistical analysis.

Funding

Meningitis Research Foundation (CSF 19-17 to ND), the Scottish Government (M/15/H/005 to ND), Irish AID, and Wellcome (206545/Z/17/Z).

Data sharing statement

An anonymised, de-identified version of the dataset can be made available on request to allow all results to be reproduced. All requests should be directed to the corresponding author.

Patient and public involvement statement

The ASPIRE mHealth primary ETAT was developed and tested, with the involvement of district and primary health level stakeholders and national policymakers.

Competing interest statement

All authors have no competing interests to disclose.

For peer review only

Contributor statement
PI conceived, led, and wrote the first draft of the manuscript. HHT conducted the statistical analysis. MG, TOB, NL, and ND led the primary ASPIRE study. All authors reviewed and approved the final manuscript.

References

1. Liu L, Oza S, Hogan D, Chu Y, Perin J, Zhu J, Lawn JE, Cousens S, Mathers C, Black RE: **Global, regional, and national causes of under-5 mortality in 2000-15: an updated systematic analysis with implications for the Sustainable Development Goals**. *Lancet* 2016, **388**(10063):3027-3035.
2. WHO: **Integrated management of childhood illness chart booklet**. 2014.
3. WHO: **Joint statement on integrated community case management**. In. Geneva, Switzerland: WHO and UNICEF; 2012.
4. WHO: **Emergency Triage Assessment and Treatment (ETAT) course**. In. Geneva, Switzerland: WHO; 2005.
5. Crouse HL, Torres F, Vaides H, Walsh MT, Ishigami EM, Cruz AT, Torrey SB, Soto MA: **Impact of an Emergency Triage Assessment and Treatment (ETAT)-based triage process in the paediatric emergency department of a Guatemalan public hospital**. *Paediatr Int Child Health* 2016, **36**(3):219-224.
6. Dekker-Boersema J, Hector J, Jefferys LF, Binamo C, Camilo D, Muganga G, Aly MM, Langa EBR, Vounatsou P, Hobbins MA: **Triage conducted by lay-staff and emergency training reduces paediatric mortality in the emergency department of a rural hospital in Northern Mozambique**. *Afr J Emerg Med* 2019, **9**(4):172-176.
7. Molyneux E, Ahmad S, Robertson A: **Improved triage and emergency care for children reduces inpatient mortality in a resource-constrained setting**. *Bull World Health Organ* 2006, **84**(4):314-319.
8. Font F, Quinto L, Masanja H, Nathan R, Ascaso C, Menendez C, Tanner M, Schellenberg J, Alonso P: **Paediatric referrals in rural Tanzania: the Kilombero District Study - a case series**. *BMC Int Health Hum Rights* 2002, **2**(1):4.
9. Kaunda W, Umali T, Chirwa ME, Nyondo-Mipando AL: **Assessing Facilitators and Barriers to Referral of Children Under the Age of Five Years at Ndirande Health Centre in Blantyre, Malawi**. *Glob Pediatr Health* 2021, **8**:2333794X211051815.
10. Johansson EW, Lindsjo C, Weiss DJ, Nsona H, Selling KE, Lufesi N, Hildenwall H: **Accessibility of basic paediatric emergency care in Malawi: analysis of a national facility census**. *BMC Public Health* 2020, **20**(1):992.
11. Nordberg E, Holmberg S, Kiugu S: **Exploring the interface between first and second level of care: referrals in rural Africa**. *Trop Med Int Health* 1996, **1**(1):107-111.
12. Simoes EA, Peterson S, Gamatie Y, Kisanga FS, Mukasa G, Nsungwa-Sabiiti J, Were MW, Weber MW: **Management of severely ill children at first-level health facilities in sub-Saharan Africa when referral is difficult**. *Bull World Health Organ* 2003, **81**(7):522-531.

For peer review only - http://bmjopen.bmj.com/site/about/guidelines.xhtml

13. al Fadil SM, Alrahman SH, Cousens S, Bustreo F, Shadoul A, Farhoud S, el Hassan SM: **Integrated Management of Childhood Illnesses strategy: compliance with referral and follow-up recommendations in Gezira State, Sudan**. *Bull World Health Organ* 2003, **81**(10):708-716.
14. Hongoro C, Musonza TG, Macq J, Anozie A: **A qualitative assessment of the referral system at district level in Zimbabwe: implications on efficiency and effective delivery of health services**. *Cent Afr J Med* 1998, **44**(4):93-97.
15. Ifeanyichi M, Broekhuizen H, Cheelo M, Juma A, Mwapasa G, Borgstein E, Kachimba J, Gajewski J, Brugha R, Pittalis C *et al*: **Surgical ambulance referrals in sub-Saharan Africa - financial costs and coping strategies at district hospitals in Tanzania, Malawi and Zambia**. *BMC Health Serv Res* 2021, **21**(1):728.
16. Peterson S, Nsungwa-Sabiiti J, Were W, Nsabagasani X, Magumba G, Nambooze J, Mukasa G: **Coping with paediatric referral--Ugandan parents' experience**. *Lancet* 2004, **363**(9425):1955-1956.
17. [https://worldpopulationreview.com/]
18. Gondwe MJ, Henrion MYR, O'Byrne T, Masesa C, Lufesi N, Dube Q, Majamanda MD, Makwero M, Lalloo DG, Desmond N: **Clinical diagnosis in paediatric patients at urban primary health care facilities in southern Malawi: a longitudinal observational study**. *BMC Health Serv Res* 2021, **21**(1):150.
19. Majamanda MD, Joshua Gondwe M, Makwero M, Chalira A, Lufesi N, Dube Q, Desmond N: **Capacity Building for Health Care Workers and Support Staff in Pediatric Emergency Triage Assessment and Treatment (ETAT) at Primary Health Care Level in Resource Limited Settings: Experiences from Malawi**. *Compr Child Adolesc Nurs* 2021:1-16.
20. Brunner NC, Omoluabi E, Awor P, Okitawutshu J, Tshefu Kitoto A, Signorell A, Akano B, Ayodeji K, Okon C, Yusuf O *et al*: **Prereferral rectal artesunate and referral completion among children with suspected severe malaria in the Democratic Republic of the Congo, Nigeria and Uganda**. *BMJ Glob Health* 2022, **7**(5).
21. Bell G, Macarayan EK, Ratcliffe H, Kim JH, Otupiri E, Lipsitz S, Hirschhorn L, Awoonor-Williams JK, Nimako BA, Ofosu A *et al*: **Assessment of Bypass of the Nearest Primary Health Care Facility Among Women in Ghana**. *JAMA Netw Open* 2020, **3**(8):e2012552.
22. Eritero AC, Gebreslasie KZ, Asgedom AT, Areba AS, Wudneh A, Bayisa Y, Molla W: **Self-referrals and associated factors among laboring mothers at Dilla University Referral Hospital, Dilla, Gedeo Zone, Ethiopia: a cross-sectional study**. *BMC Womens Health* 2022, **22**(1):417.
23. Okoli H, Obembe T, Osungbade K, Adeniji F, Adewole D: **Self-referral patterns among federal civil servants in oyo state, South-Western Nigeria**. *Pan Afr Med J* 2017, **26**:105.
24. Kozuki N, Guenther T, Vaz L, Moran A, Soofi SB, Kayemba CN, Peterson SS, Bhutta ZA, Khanal S, Tielsch JM *et al*: **A systematic review of community-to-facility neonatal referral completion rates in Africa and Asia**. *BMC Public Health* 2015, **15**:989.
25. **Integrated management of childhood illness: conclusions. WHO Division of Child Health and Development**. *Bull World Health Organ* 1997, **75 Suppl 1**(Suppl 1):119-128.
26. WHO: **Preventing injuries and violence: an overview**. In: *WHO*. Geneva: WHO; 2022.

For peer review only - http://bmjopen.bmj.com/site/about/guidelines.xhtml

27. English M, Esamai F, Wasunna A, Were F, Ogutu B, Wamae A, Snow RW, Peshu N: **Assessment of inpatient paediatric care in first referral level hospitals in 13 districts in Kenya**. *Lancet* 2004, **363**(9425):1948-1953.
28. English M, Esamai F, Wasunna A, Were F, Ogutu B, Wamae A, Snow RW, Peshu N: **Delivery of paediatric care at the first-referral level in Kenya**. *Lancet* 2004, **364**(9445):1622-1629.

For peer review only

Table 1. Main characteristics of children attending primary health centre

**Characteristics**	**Total**		**<2 months**		**2-12 months**		**>1-5 years**		**>5 years**	
	N=155,931	%	N=3,654	%	N=58,337	%	N=52,064	%	N=41,876	%
Male	77,915	50·0	1,889	51·7	29,885	51·2	26,204	50·3	19,937	47·6
Weight, kg (mean | SD)	15·34	8·33	4·09	1·22	8·48	2·39	13·74	3·42	24·59	7·18
**PHC mHealth triage**										
Emergency	392	0·3	19	0·5	162	0·3	136	0·3	75	0·2
Priority	48,248	30·9	2,974	81·4	15,694	26·9	16,912	32·5	12,668	30·3
Queue	107,291	68·8	661	18·1	42,481	72·8	35,016	67·3	29,133	69·6
**PHC clinician triage**										
Emergency	186	0·1	7	0·2	78	0·1	57	0·1	44	0·1
Priority	33,633	21·6	2,005	54·9	11,370	19·5	11,564	22·2	8,694	20·8
Queue	122,112	78·3	1,642	44·9	46,889	80·4	40,443	77·7	33,138	79·1
**PHC symptoms**										
Fever	515	0·3	23	0·7	231	0·4	166	0·3	93	0·2
Convulsions	100	0·1	1	<0·1	26	<0·1	46	0·1	27	0·1
Diarrhoea	149	0·1	1	<0·1	86	0·1	37	0·1	25	0·1
Vomiting	271	0·2	9	0·2	127	0·2	85	0·2	50	0·1
Cough	340	0·2	24	0·7	201	0·3	74	0·1	41	0·1
Difficulty breathing	309	0·2	26	0·7	199	0·3	65	0·1	19	<0·1
Rash	102	0·1	5	0·1	26	<0·1	35	0·1	36	0·1
Pallor	37	<0·1	1	<0·1	10	<0·1	19	<0·1	7	<0·1
Jaundice	13	<0·1	2	0·1	3	<0·1	5	<0·1	3	<0·1
Oedema	27	<0·1	0	0	10	<0·1	11	<0·1	6	<0·1
Urination problems	13	<0·1	3	0·1	1	<0·1	6	<0·1	3	<0·1
Feeding problems	97	0·1	5	0·1	46	0·1	27	0·1	19	<0·1
Other	1,498	1·0	50	1·4	227	0·4	557	1·1	664	1·6
**PHC laboratory test**	503	0·3	10	0·3	203	0·3	168	0·3	122	0·3
Haemoglobin	46	<0·1	1	<0·1	11	<0·1	20	<0·1	14	<0·1
Glucose	7	<0·1	0	0	1	<0·1	4	<0·1	2	<0·1
MPS	16	<0·1	0	0	3	<0·1	9	<0·1	4	<0·1
MRDT	483	0·3	9	0·2	201	0·3	159	0·3	114	0·3
Urine	4	<0·1	0	0	0	0	3	<0·1	1	<0·1
**PHC outcome**										
Admitted to short stay	181	0·1	3	0·1	67	0·1	62	0·1	49	0·1
Referred after triage	2,487	1·6	109	3·0	562	1·0	846	1·6	970	2·3
Referred after short stay	517[a]	0·3	28	0·8	128	0·2	188	0·4	173	0·4
Total referred from HC	3,004	1·9	137	3·7	690	1·2	1,034	2·0	1,143	2·7
Successful referral[b]	1,644/ 3,004	54·7	72/ 137	52·6	393/ 690	57·0	582/ 1,034	56·3	597/ 1,143	52·2
Self-referral[c]	372	0·2	21	0·6	132	0·2	130	0·2	89	0·2

For peer review only

For peer review only - http://bmjopen.bmj.com/site/about/guidelines.xhtml

**QECH A&E**										
Successful referral arrived by ambulance	53/ 1,644	3·2	2/ 137	1·5	24/ 690	3·5	16/ 1,034	1·5	11/ 1,143	1·0
Self-referral arrived by ambulance	22/ 372	5·9	3/ 21	14·3	9/ 132	6·8	5/ 130	3·8	5/ 89	5·6
Total arrived by ambulance	75/ 2,016	3·7	5/ 93	5·4	33/ 525	6·3	21/ 712	4·4	16/ 686	2·3
**QECH symptoms**										
Fever	205/ 2,016	10·2	20/ 93	21·5	111/ 525	21·1	39/ 712	5·5	35/ 686	5·1
Convulsions	32/ 2,016	1·6	1/ 93	1·1	7/ 525	1·3	20/ 712	2·8	4/ 686	0·6
Diarrhoea	49/ 2,016	2·4	2/ 93	2·2	39/ 525	7·4	4/ 712	0·6	4/ 686	0·6
Vomiting	75/ 2,016	3·7	5/ 93	5·4	41/ 525	7·8	13/ 712	1·8	16/ 686	2·3
Cough	159/ 2,016	7·9	15/ 93	16·1	94/ 525	17·9	28/ 712	3·9	22/ 686	2·8
Difficulty breathing	148/ 2,016	7·3	15/ 93	16·1	89/ 525	17·0	25/ 712	3·5	19/ 686	3·2
Rash	7/ 2,016	0·3	0	0	4/ 525	0·8	3/ 712	0·4	0	0
Pallor	25/ 2,016	1·2	0	0	6/ 525	1·1	8/ 712	1·1	11/ 686	1·6
Jaundice	11/ 2,016	0·5	7/ 93	7·5	0	0	3/ 712	0·4	1/ 686	0·1
Oedema	16/ 2,016	0·8	0	0	7/ 525	1·3	3/ 712	0·4	6/ 686	0·9
Urination problems	3/ 2,016	0·1	1/ 93	1·1	0	0	1/ 712	0·1	1/ 686	0·1
Feeding problems	52/ 2,016	2·6	8/ 93	8·6	27/ 525	5·1	6/ 712	0·8	11/ 686	1·6
Other	117/ 2,016	5·8	10/ 93	10·8	42/ 525	8·0	26/ 712	3·7	39/ 686	5·7
**QECH A&E outcome**										
Died	2/ 2290	0·1	0/ 119	0	2/ 663	0·1	0/ 770	0	0/ 738	0

A&E, accident and emergency; CSF, cerebrospinal fluid; HIV, human immunodeficiency virus; mHealth, mobile health; MRDT, malaria rapid diagnostic test; MPS, malaria parasite screen; PCV, packed cell volume; PHC, primary health centre; QECH, Queen Elizabeth Central Hospital; SD, standard deviation

[a]517 cases that were referred to QECH from PHC were still admitted to short stay at the PHC, where they were again referred to QECH.

[b]Referred by PHC and arrived at QECH.

[c]Not referred by PHC but arrived at QECH.

For peer review only

Table 2. Characteristics of triage outcome and emergency and priority signs with referral for children attending primary health centre for children ≤5 years

Characteristics	Referred		Not referred		Successfully referred		Not successfully referred		Self-referred	
	N=1,861	%	N=112,194	%	N=1,047	%	N=815	%	N=283	%
**Emergency signs**										
**Breathing**										
Obstructed breathing	28	1·5	102	0·1	19	1·8	9	1·1	0	0·0
Central cyanosis	1	0·1	4	<0·1	0	0	1	0·1	0	0
Respiratory distress	35	1·9	82	0·1	21	2·0	14	1·7	5	1·8
**Circulation**										
Cold hands	11	0·6	1,214	1·1	5	0·5	6	0·7	7	2·5
Capillary refill >3 seconds	0	0	8	<0·1	0	0	0	0	0	0
Weak and fast pulse	1	0·1	20	<0·1	1	0·1	0	0	0	0
**Consciousness**										
Coma	3	0·2	3	<0·1	1	0·1	2	0·2	1	0·4
Convulsions	24	1·3	29	<0·1	15	1·4	9	1·1	3	1·1
**Dehydration**										
Diarrhoea	130	7·0	22,321	19·9	85	8·1	45	5·5	48	17·0
Lethargic or unconscious	6	0·3	118	0·1	2	0·2	4	0·5	2	0·7
Sunken eyes	16	0·9	280	0·2	13	1·2	3	0·4	6	2·1
Very slow skin pinch	1	0·1	60	0·1	1	0·1	0	0	0	0·0
**Priority signs**										
Tiny baby	137	7·4	3,856	3·4	76	7·3	62	7·6	26	9·2
Temperature	229	12·3	26,390	23·5	136	13·0	93	11·4	74	26·1
Trauma	497	26·7	1,619	1·4	304	29·1	193	23·7	17	6·0
Severe pallor	3	0·2	241	0·2	0	0	3	0·4	0	0
Poisoning	4	0·2	19	<0·1	3	0·3	1	0·1	0	0
Severe pain	100	5·4	1,050	0·9	52	5·0	48	5·9	14	5·0
Respiratory distress	47	2·5	798	0·7	35	3·3	12	1·5	9	3·2
Restless/lethargic	5	0·3	125	0·1	4	0·4	1	0·1	3	1·1
Urgent referral	7	0·4	16	<0·1	3	0·3	4	0·5	0	0
Malnutrition	12	0·6	41	<0·1	6	0·6	6	0·7	2	0·7
Oedema	9	0·5	46	<0·1	3	0·3	6	0·7	0	0
Burns	55	0·3	274	0·2	19	1·8	36	4·4	2	0·7

For peer review only

For peer review only - http://bmjopen.bmj.com/site/about/guidelines.xhtml

Triage outcome										
Emergency	85	4·6	57	0·1	46	4·4	39	4·8	5	1·8
Priority	1,315	70·7	23,624	21·1	770	73·5	546	67·0	116	41·0
Queue	461	24·8	88,513	78·9	231	22·1	230	28·2	162	57·2

For peer review only

Table 3. Characteristics of children discharged from the primary health centre with subsequent self-referral to hospital

Characteristics	Total		<2 months		2-12 months		>1-5 years		>5 years	
	N=372	%	N=21	%	N=132	%	N=130	%	N=89	%
Male	215	57·9	9	42·9	72	54·5	72	55·4	62	69·7
Weight, kg (mean | SD)	11·80	4·18	-	-	4·90	-	13·03	1·95	15.00	-
**PHC mHealth triage**										
Emergency	11	3·0	0	0	5	3·8	5	3·8	1	1·1
Priority	197	53·0	21	100	68	51·5	58	44·6	50	56·2
Queue	164	44·1	0	0	59	44·7	67	51·5	38	42·7
**PHC clinician triage**										
Emergency	1	1·3	0	0	2	1·5	3	2·3	0	0
Priority	145	39·0	17	81·0	55	41·7	44	33·8	29	32·6
Queue	222	59·7	4	19·0	75	56·8	83	63·8	60	67·4
**Emergency signs**										
**Breathing**										
Obstructed breathing	1	0·3	0	0	0	0	0	0	1	1·1
Central cyanosis	0	0	0	0	0	0	0	0	0	0
Respiratory distress	5	1·3	0	0	2	1·5	3	2·3	0	0
**Circulation**										
Cold hands	9	2·4	0	0	5	3·8	2	1·5	2	2·2
Capillary refill >3 seconds	0	0	0	0	0	0	0	0	0	0
Weak and fast pulse	0	0	0	0	0	0	0	0	0	0
**Consciousness**										
Coma	1	0·3	0	0	1	0·8	0	0	0	0
Convulsions	3	0·8	0	0	2	1·5	1	0·8	0	0
**Dehydration**										
Diarrhoea	54	14·5	3	14·3	35	26·5	10	7·7	6	6·7
Lethargic or unconscious	2	0·5	0	0	1	0·8	1	0·8	0	0
Sunken eyes	6	1·6	0	0	5	3·8	1	0·8	0	0
Very slow skin pinch	0	0	0	0	0	0	0	0	0	0
**Priority signs**										
Tiny baby	26	7·0	19	90·5	6	4·5	1	0·8	0	0
Temperature	90	24·2	2	9·5	40	30·3	32	24·6	16	18·0
Trauma	36	9·7	0	0	5	3·8	12	9·2	19	21·3
Severe pallor	0	0	0	0	0	0	0	0	0	0
Poisoning	0	0	0	0	0	0	0	0	0	0
Severe pain	23	6·2	0	0	5	3·8	9	6·9	9	10·1
Respiratory distress	11	3·0	0	0	7	5·3	2	1·5	2	2·2
Restless/ lethargic	5	1·3	0	0	2	1·5	1	0·8	2	2·2
Urgent referral	1	0·3	0	0	0	0	0	0	1	1·1

For peer review only - http://bmjopen.bmj.com/site/about/guidelines.xhtml

For peer review only

Malnutrition	2	0·5	0	0	2	1·5	0	0	0	0
Oedema	0	0	0	0	0	0	0	0	0	0
Burns	3	0·8	0	0	1	0·8	1	0·8	1	1·1
**QECH diagnosis**										
Trauma	1	0·3	0	0	0	0	0	0	1	1·1
Gastroenteritis	14	3·8	1	4·8	12	9·1	1	0·8	0	0
Pneumonia	16	4·3	1	4·8	12	9·1	3	2·3	0	0
Meningitis	2	0·5	0	0	1	0·8	1	0·8	0	0
Malnutrition	10	2·7	0	0	6	4·5	2	1·5	2	2·2
Sepsis	4	1·1	3	14·3	1	0·8	0	0	0	0
Malaria	15	4·0	0	0	5	3·8	7	5·4	3	3·4
Anaemia	6	1·6	0	0	3	2·3	2	1·5	1	1·1
Other	55	14·8	4	19·0	25	18·9	12	9·2	14	15·7
**QECH laboratory test result**										
Positive blood culture	2	0·5	0	0	0	0	0	0	2	2·2
Positive CSF culture	6	1·6	1	4·8	2	1·5	2	1·5	1	1·1
Positive HIV test	0	0	0	0	0	0	0	0	0	0
Positive MRDT	7	1·9	0	0	2	1·5	3	2·3	2	2·2
Positive MPS	9	2·4	0	0	6	4·5	1	0·8	2	2·2
Low blood glucose	2	0·5	0	0	1	0·8	0	0	1	1·1
PCV, (mean | SD)	32·34	6·61	36·25	1·89	31·00	5·89	35·60	2·30	30·80	11·99
**QECH A&E outcome**										
Died	2	0·5	0	0	2	1·5	0	0	0	0

A&E, accident and emergency; CSF, cerebrospinal fluid; HIV, human immunodeficiency virus; mHealth, mobile health; MPS, malaria parasite smear; MRDT, malaria rapid diagnostic test; PCV, packed cell volume; PHC, primary health clinic; QECH, Queen Elizabeth Central Hospital

For peer review only

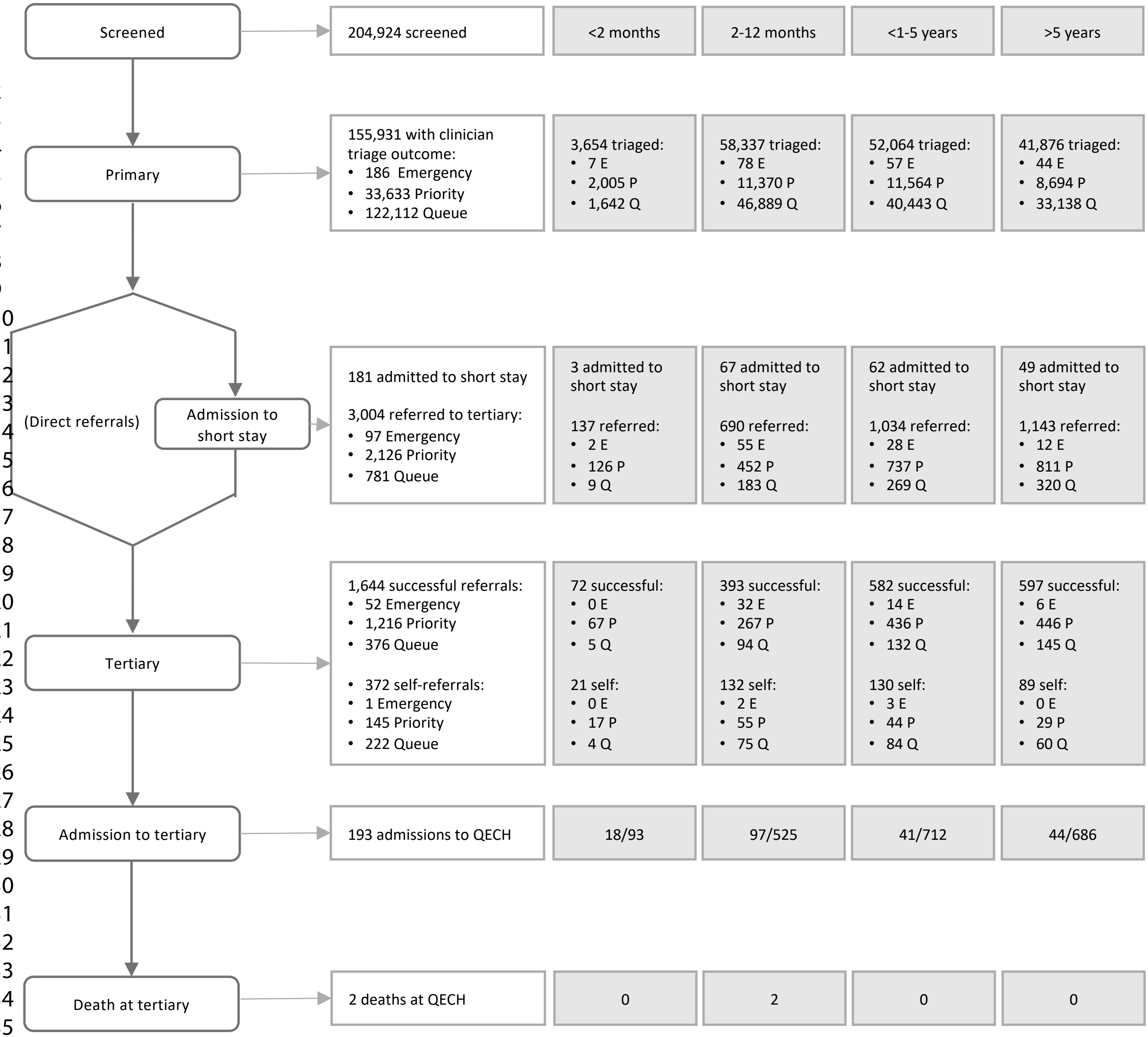
Screened
204,924 screened
<2 months
2-12 months
<1-5 years
>5 years
Primary
155,931 with clinician triage outcome:
• 186 Emergency
• 33,633 Priority
• 122,112 Queue
3,654 triaged:
• 7 E
• 2,005 P
• 1,642 Q
58,337 triaged:
• 78 E
• 11,370 P
• 46,889 Q
52,064 triaged:
• 57 E
• 11,564 P
• 40,443 Q
41,876 triaged:
• 44 E
• 8,694 P
• 33,138 Q
(Direct referrals)
Admission to short stay
181 admitted to short stay
3,004 referred to tertiary:
• 97 Emergency
• 2,126 Priority
• 781 Queue
3 admitted to short stay
137 referred:
• 2 E
• 126 P
• 9 Q
67 admitted to short stay
690 referred:
• 55 E
• 452 P
• 183 Q
62 admitted to short stay
1,034 referred:
• 28 E
• 737 P
• 269 Q
49 admitted to short stay
1,143 referred:
• 12 E
• 811 P
• 320 Q
Tertiary
1,644 successful referrals:
• 52 Emergency
• 1,216 Priority
• 376 Queue
• 372 self-referrals:
• 1 Emergency
• 145 Priority
• 222 Queue
72 successful:
• 0 E
• 67 P
• 5 Q
21 self:
• 0 E
• 17 P
• 4 Q
393 successful:
• 32 E
• 267 P
• 94 Q
132 self:
• 2 E
• 55 P
• 75 Q
582 successful:
• 14 E
• 436 P
• 132 Q
130 self:
• 3 E
• 44 P
• 84 Q
597 successful:
• 6 E
• 446 P
• 145 Q
89 self:
• 0 E
• 29 P
• 60 Q
Admission to tertiary
193 admissions to QECH
18/93
97/525
41/712
44/686
Death at tertiary
2 deaths at QECH
0
2
0
0

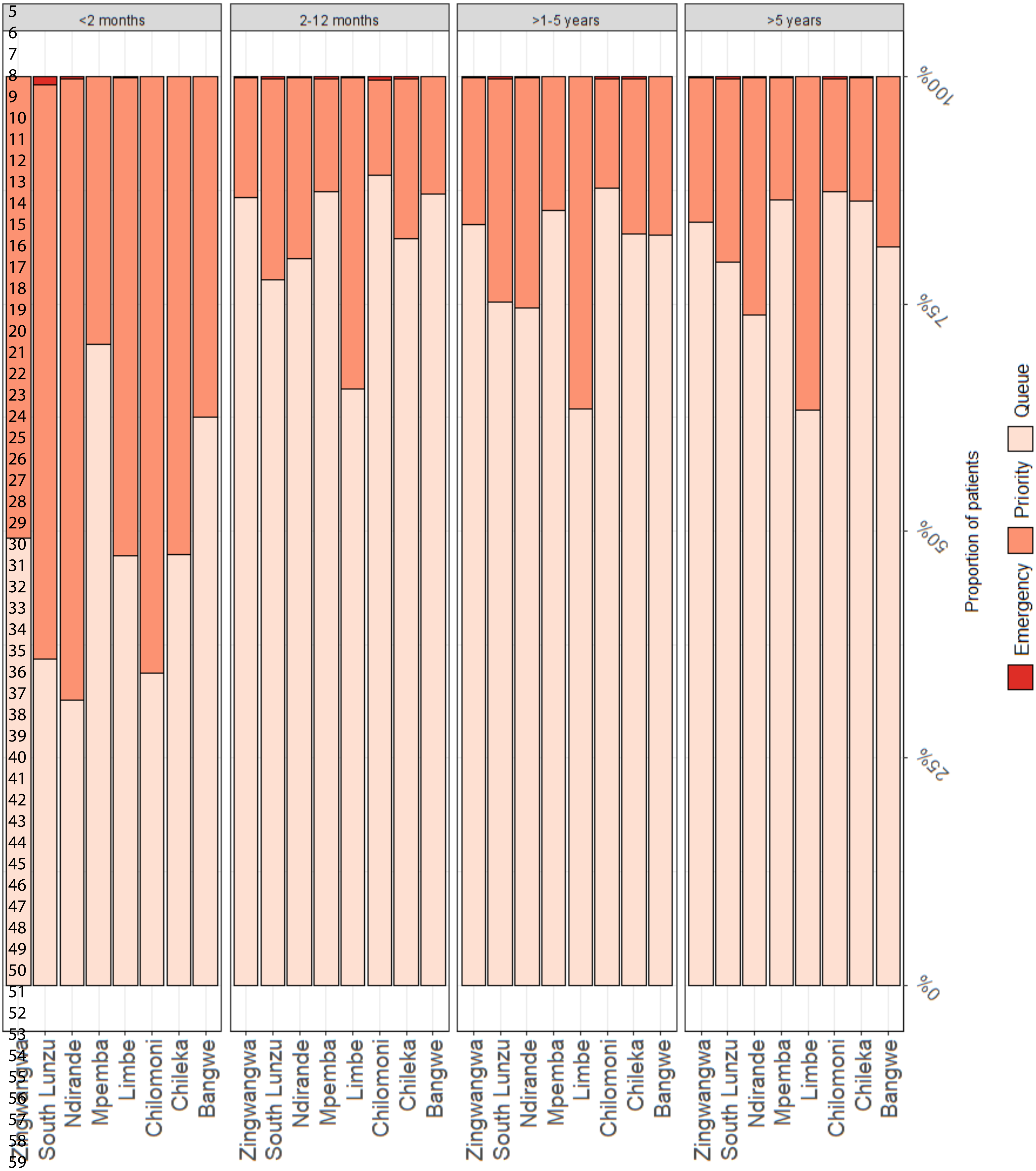
<2 months
2-12 months
>1-5 years
>5 years
Zingwangwa
South Lunzu
Ndirande
Mpemba
Limbe
Chilomoni
Chileka
Bangwe
100%
75%
50%
25%
0%
Proportion of patients
Emergency
Priority
Queue

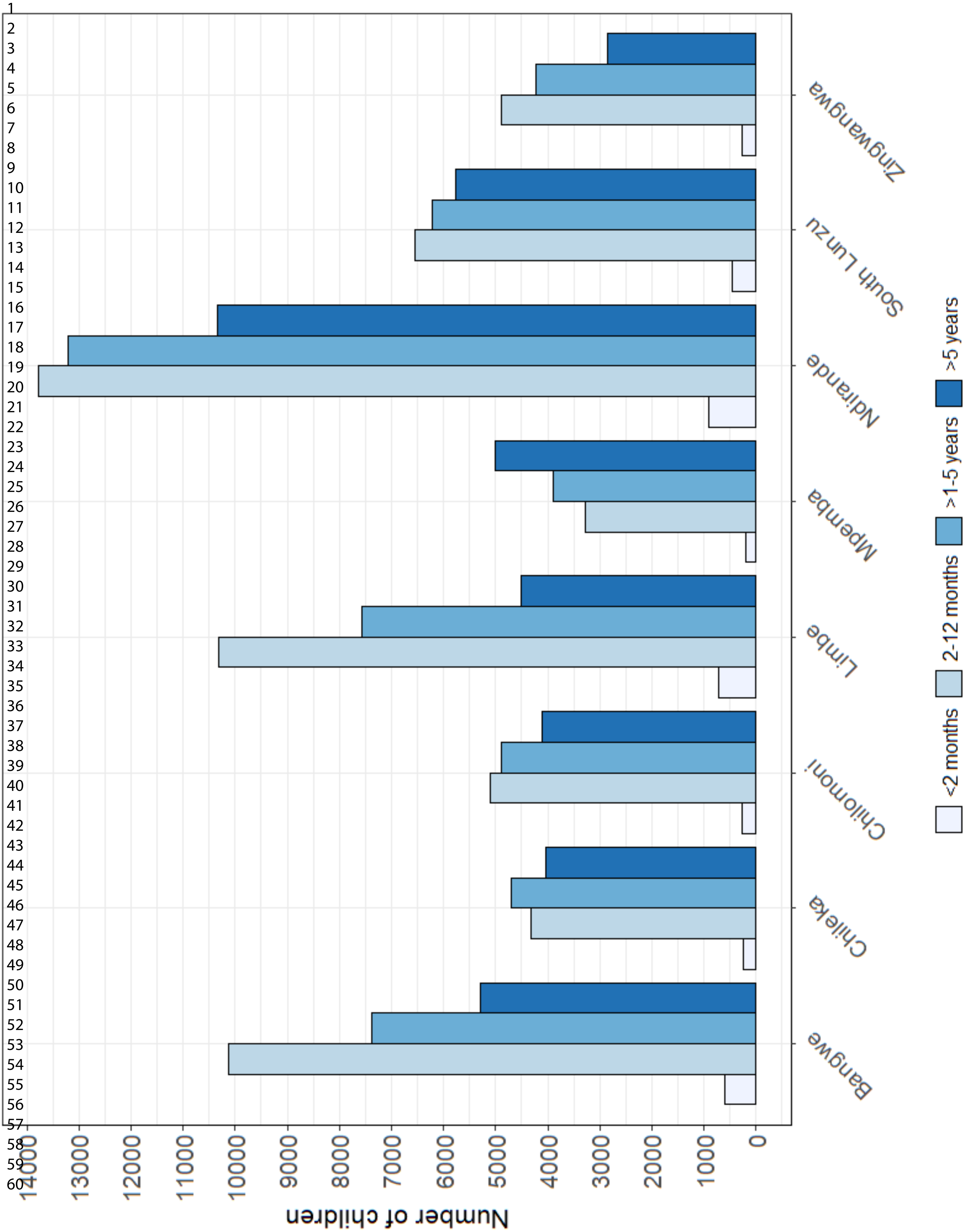
Zingwangwa
South Lunzu
Ndirande
Mpemba
Limbe
Chilomoni
Chileka
Bangwe
<2 months
2-12 months
>1-5 years
>5 years
Number of children
14000
13000
12000
11000
10000
9000
8000
7000
6000
5000
4000
3000
2000
1000
0

Supplementary Table 1. Complete characteristics of children attending primary health centre

Characteristics	Total		<2 months		2-12 months		>1-5 years		>5 years	
	N=155,931	%	N=3,654	%	N=58,337	%	N=52,064	%	N=41,876	%
Male	77,915	50·0	1,889	51·7	29,885	51·2	26,204	50·3	19,937	47·6
Weight, kg (mean | SD)	15·34	8·33	4·09	1·22	8·48	2·39	13·74	3·42	24·59	7·18
**PHC**										
Bangwe	23,389	15·0	604	16·5	10,115	17·3	7,388	14·2	5,282	12·6
Chileka	13,266	8·5	236	6·5	4,309	7·4	4,694	9·0	4,027	9·6
Chilomoni	14,342	9·2	265	7·3	5,097	8·7	4,878	9·4	4,102	9·8
Limbe	23,099	14·8	713	19·5	10,313	17·7	7,564	14·5	4,509	10·8
Mpemba	12,388	7·9	207	5·7	3,277	5·6	3,904	7·5	5,000	11·9
Ndirande	38,243	24·5	912	25·0	13,799	23·6	13,210	25·4	10,342	24·7
South Lunzu	18,990	12·2	457	12·5	6,557	11·2	6,207	11·9	5,769	13·8
Zingwangwa	12,214	7·8	260	7·1	4,890	8·4	4,219	8·1	2,845	6·8
**PHC mHealth triage**										
Emergency	392	0·3	19	0·5	162	0·3	136	0·3	75	0·2
Priority	48,248	30·9	2,974	81·4	15,694	26·9	16,912	32·5	12,668	30·3
Queue	107,291	68·8	661	18·1	42,481	72·8	35,016	67·3	29,133	69·6
**PHC clinician triage**										
Emergency	186	0·1	7	0·2	78	0·1	57	0·1	44	0·1
Priority	33,633	21·6	2,005	54·9	11,370	19·5	11,564	22·2	8,694	20·8
Queue	122,112	78·3	1,642	44·9	46,889	80·4	40,443	77·7	33,138	79·1
**PHC symptoms**										
Fever	515	0·3	23	0·7	231	0·4	166	0·3	93	0·2
Convulsions	100	0·1	1	<0·1	26	<0·1	46	0·1	27	0·1
Diarrhoea	149	0·1	1	<0·1	86	0·1	37	0·1	25	0·1
Vomiting	271	0·2	9	0·2	127	0·2	85	0·2	50	0·1
Cough	340	0·2	24	0·7	201	0·3	74	0·1	41	0·1
Difficulty breathing	309	0·2	26	0·7	199	0·3	65	0·1	19	<0·1
Rash	102	0·1	5	0·1	26	<0·1	35	0·1	36	0·1
Pallor	37	<0·1	1	<0·1	10	<0·1	19	<0·1	7	<0·1
Jaundice	13	<0·1	2	0·1	3	<0·1	5	<0·1	3	<0·1
Oedema	27	<0·1	0	0	10	<0·1	11	<0·1	6	<0·1
Urination problems	13	<0·1	3	0·1	1	<0·1	6	<0·1	3	<0·1
Feeding problems	97	0·1	5	0·1	46	0·1	27	0·1	19	<0·1
Other	1,498	1·0	50	1·4	227	0·4	557	1·1	664	1·6
**PHC laboratory test**	503	0·3	10	0·3	203	0·3	168	0·3	122	0·3
Haemoglobin	46	<0·1	1	<0·1	11	<0·1	20	<0·1	14	<0·1
Glucose	7	<0·1	0	0	1	<0·1	4	<0·1	2	<0·1
MPS	16	<0·1	0	0	3	<0·1	9	<0·1	4	<0·1
MRDT	483	0·3	9	0·2	201	0·3	159	0·3	114	0·3
Urine	4	<0·1	0	0	0	0	3	<0·1	1	<0·1
**PHC outcome**										
Admitted to short stay	181	0·1	3	0·1	67	0·1	62	0·1	49	0·1
Referred after triage	2,487	1·6	109	3·0	562	1·0	846	1·6	970	2·3
Referred after short stay	517[a]	0·3	28	0·8	128	0·2	188	0·4	173	0·4
Total referred from HC	3,004	1·9	137	3·7	690	1·2	1,034	2·0	1,143	2·7
Successful referral[b]	1,644/ 3,004	54·7	72/ 137	52·6	393/ 690	57·0	582/ 1,034	56·3	597/ 1,143	52·2
Self-referral[c]	372	0·2	21	0·6	132	0·2	130	0·2	89	0·2
**QECH A&E**										
Successful referral arrived by ambulance	53/ 1,644	3·2	2/ 137	1·5	24/ 690	3·5	16/ 1,034	1·5	11/ 1,143	1·0
Self-referral arrived by ambulance	22/ 372	5·9	3/ 21	14·3	9/ 132	6·8	5/ 130	3·8	5/ 89	5·6
Total arrived by ambulance	75/ 2,016	3·7	5/ 93	5·4	33/ 525	6·3	21/ 712	4·4	16/ 686	2·3
**QECH symptoms**										

For peer review only - http://bmjopen.bmj.com/site/about/guidelines.xhtml

Fever	205/ 2,016	10·2	20/ 93	21·5	111/ 525	21·1	39/ 712	5·5	35/ 686	5·1
Convulsions	32/ 2,016	1·6	1/ 93	1·1	7/ 525	1·3	20/ 712	2·8	4/ 686	0·6
Diarrhoea	49/ 2,016	2·4	2/ 93	2·2	39/ 525	7·4	4/ 712	0·6	4/ 686	0·6
Vomiting	75/ 2,016	3·7	5/ 93	5·4	41/ 525	7·8	13/ 712	1·8	16/ 686	2·3
Cough	159/ 2,016	7·9	15/ 93	16·1	94/ 525	17·9	28/ 712	3·9	22/ 686	2·8
Difficulty breathing	148/ 2,016	7·3	15/ 93	16·1	89/ 525	17·0	25/ 712	3·5	19/ 686	3·2
Rash	7/ 2,016	0·3	0	0	4/ 525	0·8	3/ 712	0·4	0	0
Pallor	25/ 2,016	1·2	0	0	6/ 525	1·1	8/ 712	1·1	11/ 686	1·6
Jaundice	11/ 2,016	0·5	7/ 93	7·5	0	0	3/ 712	0·4	1/ 686	0·1
Oedema	16/ 2,016	0·8	0	0	7/ 525	1·3	3/ 712	0·4	6/ 686	0·9
Urination problems	3/ 2,016	0·1	1/ 93	1·1	0	0	1/ 712	0·1	1/ 686	0·1
Feeding problems	52/ 2,016	2·6	8/ 93	8·6	27/ 525	5·1	6/ 712	0·8	11/ 686	1·6
Other	117/ 2,016	5·8	10/ 93	10·8	42/ 525	8·0	26/ 712	3·7	39/ 686	5·7
**QECH diagnosis**										
Trauma	9/ 2,016	0·4	0	0	2/ 525	0·4	4/ 712	0·6	3/ 686	0·4
Gastroenteritis	35/ 2,016	1·7	2/ 93	2·2	29/ 525	5·5	2/ 712	0·3	2/ 686	0·3
Pneumonia	52/ 2,016	2·6	4/ 93	4·3	37/ 525	7·0	5/ 712	0·7	6/ 686	0·9
Meningitis	6/ 2,016	0·3	0	0	2/ 525	0·4	3/ 712	0·4	1/ 686	0·1
Malnutrition	25/ 2,016	1·2	1/ 93	1·1	16/ 525	3·0	4/ 712	0·6	4/ 686	0·6
Sepsis	16/ 2,016	0·8	13/ 93	14·0	3/ 525	0·6	0	0	0	0
Malaria	41/ 2,016	2·0	0	0	14/ 525	2·7	18/ 712	2·5	9/ 686	1·3
Anaemia	26/ 2,016	1·3	1/ 93	1·1	8/ 525	1·5	7/ 712	0·9	10/ 686	1·5
Other	201/ 2,016	10·0	19/ 93	2-·4	97/ 525	18·5	41/ 712	5·8	44/ 686	6·4
**QECH laboratory test**	193/ 2,016	9·6	18/ 93	19·4	97/ 525	18·5	47/ 712	5·8	36/ 686	5·2
CSF culture	11/ 2,016	0·5	4/ 93	4·3	2/ 525	0·4	3/ 712	0·4	2/ 686	0·3
Blood culture	41/ 2,016	2·0	10/ 93	10·8	15/ 525	2·9	5/ 712	0·7	11/ 686	1·6
Blood glucose	23/ 2,016	1·1	2/ 93	2·2	15/ 525	2·9	3/ 712	0·4	3/ 686	0·4
HIV	148/ 2,016	7·3	12/ 93	12·9	74/ 525	14·1	32/ 712	4·5	30/ 686	4·4
MRDT	90/ 2,016	4·5	7/ 93	7·5	42/ 525	8·0	22/ 712	3·1	19/ 686	2·8
MPS	127/ 2,016	6·3	13/ 93	14·0	66/ 525	12·6	23/ 712	3·2	25/ 686	3·6
PCV	127/ 2,016	6·3	13/ 93	14·0	67/ 525	12·8	22/ 712	3·1	25/ 686	3·6
Other	38/ 2,016	1·9	1/ 93	1·1	13/ 525	2·5	8/ 712	1·1	16/ 686	2·3
**QECH laboratory test result**										
Positive blood culture	5/ 41	12·2	1/ 10	10	0/ 15	0	1/ 5	20	3/ 11	27
Positive CSF culture	11/ 11	100	4/ 4	100	2/ 2	100	3/ 3	100	2/2	100
Positive HIV test	8/ 148	5·4	0/ 12	0	2/ 74	27	0/ 32	0	6/ 30	20
Positive MRDT	17/ 90	18·9	0/ 7	0	5/ 42	12	7/ 22	32	5/ 19	26
Positive MPS	19/ 127	15·0	0/ 13	0	5/ 66	8	7/ 23	30	5/ 25	20
Low blood glucose	5/ 23	21·7	1/ 2	50	2/ 15	14	0/ 3	0	2/ 3	67
PCV (mean | SD)	32·17	8·11	35·62	7·59	32·76	6·11	33·68	8·12	27·44	11·12
**QECH A&E outcome**										
Died	2/ 2290	0·1	0/ 119	0	2/ 663	0·1	0/ 770	0	0/ 738	0

A&E, accident and emergency; CSF, cerebrospinal fluid; HIV, human immunodeficiency virus; mHealth, mobile health; MRDT, malaria rapid diagnostic test; MPS, malaria parasite screen; PCV, packed cell volume; PHC, primary health centre; QECH, Queen Elizabeth Central Hospital; SD, standard deviation

[a]517 cases that were referred to QECH from PHC were still admitted to short stay at the PHC, where they were again referred to QECH.

[b]Referred by PHC and arrived at QECH.

[c]Not referred by PHC but arrived at QECH.

For peer review only

Supplementary Table 2A. For young infants <2 months, emergency and priority signs, referral, and successful referral

	Referred		Not referred		Successfully referred		Not successfully referred	
	N=137	%	N=3,517	%	N=72	%	N=65	%
**Emergency signs**								
**Breathing**								
Obstructed breathing	1	0.7	8	0.2	0	0	1	1.5
Central cyanosis	0	0	1	<0.1	0	0	0	0
Respiratory distress	1	0.7	8	0.2	0	0	1	1.5
**Circulation**								
Cold hands	0	0	45	1.3	0	0	0	0
Capillary refill >3 secs	0	0	1	2.2	0	0	0	0
Weak and fast pulse	0	0	1	2.2	0	0	0	0
**Consciousness**								
Coma	0	0	0	0	0	0	0	0
Convulsions	1	0.7	0	0	0	0	1	1.5
**Dehydration**								
Diarrhoea	4	3.0	195	5.6	1	1.4	3	4.6
Lethargic or unconscious	0	0	3,306	94.0	0	0	0	0
Sunken eyes	0	0	0	0	0	0	0	0
Very slow skin pinch	0	0	0	0	0	0	0	0
**Priority signs**								
Tiny baby	108	78.2	2,616	74.4	60	83.3	49	74.2
Temperature	7	5.1	181	5.1	3	4.2	4	6.1
Trauma	2	1.5	10	0.3	1	1.4	1	1.5
Severe pain	0	0	28	0.8	0	0	0	0
Respiratory distress	0	0	21	0.6	0	0	0	0
Burns	0	0	1	<0.1	0	0	0	0

Supplementary Table 2B. For infants 2-12 months, emergency and priority signs, referral, and successful referral

	Referred		Not referred		Successfully referred		Not successfully referred	
	N=690	%	N=57,647	%	N=393	%	N=297	%
**Emergency signs**								
**Breathing**								
Obstructed breathing	23	3.3	49	<0.1	17	4.3	6	2.0
Central cyanosis	0	0	0	0	0	0	0	0

For peer review only

Respiratory distress	25	3.6	44	<0.1	16	4.1	9	3.0
**Circulation**								
Cold hands	3	0.4	621	1.1	1	0.3	2	0.7
Capillary refill >3 secs	0	0	5	<0.1	0	0	0	0
Weak and fast pulse	0	0	8	<0.1	0	0	0	0
**Consciousness**								
Coma	0	0	1	<0.1	0	0	0	0
Convulsions	7	1.1	9	<0.1	5	1.3	2	0.7
**Dehydration**								
Diarrhoea	89	12.9	13,828	24.0	62	15.8	27	9.1
Lethargic or unconscious	3	0.4	66	0.1	2	0.5	1	0.3
Sunken eyes	15	2.2	182	0.3	13	3.3	2	0.7
Very slow skin pinch	1	0.1	34	0.1	1	0.3	0	0
**Priority signs**								
Tiny baby	29	4.2	1,152	2.0	16	4.1	13	4.4
Temperature	133	19.3	12,383	21.5	80	20.4	53	17.8
Trauma	94	13.6	580	1.0	61	15.5	33	11.1
Severe pallor	1	0.1	32	<0.1	0	0	1	0.3
Poisoning	3	0.4	7	<0.1	3	0.8	0	0
Severe pain	33	4.8	391	0.7	16	4.1	17	5.7
Respiratory distress	31	4.5	533	0.9	22	5.6	9	3.0
Restless/lethargic	4	0.6	81	0.1	4	1.0	0	0
Urgent referral	4	0.6	8	<0.1	3	0.8	1	0.3
Malnutrition	7	1.0	26	<0.1	6	1.5	1	0.3
Oedema	2	0.3	18	<0.1	1	0.3	1	0.3
Burns	17	2.5	125	0.2	3	0.8	14	4.7

For peer review only

Supplementary Table 2C. For children >1-5 years, emergency and priority signs, referral, and successful referral

	Referred		Not referred		Successfully referred		Not successfully referred	
	N=1,034	%	N=51,030	%	N=582	%	N=452	%
**Emergency signs**								
**Breathing**								
Obstructed breathing	4	0.4	45	<0.1	2	0.3	2	0.4
Central cyanosis	1	<0.1	3	<0.1	0	0	1	0.2
Respiratory distress	9	0.9	30	<0.1	5	0.9	4	0.9
**Circulation**								
Cold hands	8	0.8	548	1.1	4	0.7	4	0.9
Capillary refill >3 secs	0	0	2	<0.1	0	0	0	0
Weak and fast pulse	1	0.1	11	<0.1	1	0.2	0	0
**Consciousness**								
Coma	3	0.3	2	<0.1	1	0.2	2	0.4
Convulsions	16	1.6	20	<0.1	10	1.7	6	1.3
**Dehydration**								

Diarrhoea	37	3.7	8,298	16.3	22	3.8	15	3.3
Lethargic or unconscious	3	0.3	52	0.1	0	0	3	0.7
Sunken eyes	1	0.1	98	0.2	0	0	1	0.2
Very slow skin pinch	0	0	26	0.1	0	0	0	0
**Priority signs**								
Tiny baby	0	0	88	0.2	0	0	0	0
Temperature	89	8.6	13,826	27.1	53	9.1	36	8.0
Trauma	401	38.8	1,029	2.0	242	41.6	159	35.2
Severe pallor	2	0.2	209	0.4	0	0	2	0.4
Poisoning	1	<0.1	12	<0.1	0	0	1	0.2
Severe pain	67	6.5	631	1.2	36	6.2	31	6.9
Respiratory distress	16	1.5	244	0.5	13	2.2	3	0.7
Restless/lethargic	1	<0.1	44	<0.1	0	0	1	0.2
Urgent referral	3	0.3	8	<0.1	0	0	3	0.7
Malnutrition	5	0.5	15	<0.1	0	0	5	1.1
Oedema	7	0.7	28	<0.1	2	0.3	5	1.1
Burns	38	3.7	148	0.3	16	2.7	22	4.9

Supplementary Table 2D. For children >5 years, emergency and priority signs, referral, and successful referral

	Referred		Not referred		Successfully referred		Not successfully referred	
	N=1,143	%	N=40,733	%	N=597	%	N=546	%
**Emergency signs**								
**Breathing**								
Obstructed breathing	1	<0.1	31	<0.1	0	0	1	0.2
Central cyanosis	0	0	0	0	0	0	0	0
Respiratory distress	1	<0.1	13	<0.1	1	0.2	0	0
**Circulation**								
Cold hands	2	0.2	367	0.9	1	0.2	1	0.2
Capillary refill >3 secs	0	0	4	1.1	0	0	0	0
Weak and fast pulse	1	<0.1	10	2.7	1	0.2	0	0
**Consciousness**								
Coma	3	0.3	8	<0.1	2	0.3	1	0.2
Convulsions	4	0.4	14	<0.1	3	0.5	1	0.2
**Dehydration**								
Diarrhoea	30	2.6	5,040	12.4	16	2.7	14	2.6
Lethargic or unconscious	0	0	18	0.4	0	0	0	0
Sunken eyes	0	0	46	0.9	0	0	0	0
Very slow skin pinch	0	0	9	0.2	0	0	0	0
**Priority signs**								
Tiny baby	0	0	36	<0.1	0	0	0	0
Temperature	84	7.3	9,928	24.3	46	7.7	38	7.0
Trauma	514	45.0	920	2.3	294	49.2	220	40.3
Severe pallor	0	0	55	0.1	0	0	0	0

For peer review only

Poisoning	2	0.2	11	<0.1	1	0.2	1	0.2
Severe pain	105	9.2	750	1.8	58	9.7	47	8.6
Respiratory distress	2	0.2	128	0.3	2	0.3	0	0
Restless/lethargic	1	<0.1	29	<0.1	0	0	1	0.2
Urgent referral	1	<0.1	7	<0.1	1	0.2	0	0
Malnutrition	0	0	6	<0.1	0	0	0	0
Oedema	4	0.3	8	<0.1	2	0.3	2	0.4
Burns	10	0.9	67	0.2	2	0.3	8	1.5

For peer review only

Supplementary Table 3. Referrals, and successful referrals by triage outcome and age group

Age group	Triage outcome	Referred		Not referred		Successfully referred		Not successfully referred	
		N	%	N	%	N	%	N	%
Overall	Emergency	97/ 3,004	3.2	89/ 152,927	0.1	52/ 1,644	3.2	45/ 1,361	3.3
	Priority	2,126/ 3,004	70.8	31,507/ 152,927	20.6	1,216/ 1,644	74.0	911/ 1,361	66.9
	Queue	781/ 3,002	26.0	121,331/ 152,927	79.3	376/ 1,644	22.9	405/ 1,361	29.8
<2 months	Emergency	2/ 137	1.5	5/ 3,517	0.1	0/ 72	0	2/ 65	3.0
	Priority	126/ 137	92.0	1,879	53.4	67/ 72	93.1	59/ 65	90.8
	Queue	9/ 137	6.6	1,633	46.4	5/ 72	6.9	4/ 65	6.2
2-12 months	Emergency	55/ 690	8.0	23/ 57,647	<0.1	32/ 393	8.1	23/ 297	7.7
	Priority	452/ 690	65.5	10,918/ 57,647	18.9	267/ 393	67.9	185/ 297	62.3
	Queue	183/ 690	26.5	46,706/ 57,647	81.0	94/ 393	23.9	89/ 297	30.0
>1-5 years	Emergency	28/ 1,034	2.7	29/ 51,030	0.1	14/ 582	2.4	14/ 452	3.1
	Priority	737/ 1,034	71.3	10,827/ 51,030	21.2	436/ 582	74.9	301/ 452	66.6
	Queue	269/ 1,034	26.0	40,174/ 51,030	78.7	132/ 582	22.7	137/ 452	30.3
>5 years	Emergency	12/ 1,143	1.0	32/ 40,733	0.1	6/ 597	1.0	6/ 546	1.1
	Priority	811/ 1,143	71.0	7,883/ 40,733	19.4	446/ 597	74.7	365/ 546	66.8
	Queue	320/ 1,143	28.0	32,818/ 40,733	80.6	145/ 597	24.3	175/ 546	32.1

For peer review only

STROBE Statement—checklist of items that should be included in reports of observational studies

	Item No.	Recommendation	Page No.	Relevant text from manuscript
**Title and abstract**	1	(*a*) Indicate the study's design with a commonly used term in the title or the abstract	1	
		(*b*) Provide in the abstract an informative and balanced summary of what was done and what was found	2	
**Introduction**				
Background/rationale	2	Explain the scientific background and rationale for the investigation being reported	4	
Objectives	3	State specific objectives, including any prespecified hypotheses	4-5	
**Methods**				
Study design	4	Present key elements of study design early in the paper	5	
Setting	5	Describe the setting, locations, and relevant dates, including periods of recruitment, exposure, follow-up, and data collection	5-6	
Participants	6	(*a*) *Cohort study*—Give the eligibility criteria, and the sources and methods of selection of participants. Describe methods of follow-up<br>*Case-control study*—Give the eligibility criteria, and the sources and methods of case ascertainment and control selection. Give the rationale for the choice of cases and controls<br>*Cross-sectional study*—Give the eligibility criteria, and the sources and methods of selection of participants	6	
		(*b*) *Cohort study*—For matched studies, give matching criteria and number of exposed and unexposed<br>*Case-control study*—For matched studies, give matching criteria and the number of controls per case	NA	
Variables	7	Clearly define all outcomes, exposures, predictors, potential confounders, and effect modifiers. Give diagnostic criteria, if applicable	6	
Data sources/ measurement	8*	For each variable of interest, give sources of data and details of methods of assessment (measurement). Describe comparability of assessment methods if there is more than one group	*6*	
Bias	9	Describe any efforts to address potential sources of bias	6-7	
Study size	10	Explain how the study size was arrived at	7	

Continued on next page

For peer review only - http://bmjopen.bmj.com/site/about/guidelines.xhtml
For peer review only

Quantitative variables	11	Explain how quantitative variables were handled in the analyses. If applicable, describe which groupings were chosen and why	6-7
Statistical methods	12	(*a*) Describe all statistical methods, including those used to control for confounding	6-7
		(*b*) Describe any methods used to examine subgroups and interactions	6-7
		(*c*) Explain how missing data were addressed	6-7
		(*d*) *Cohort study*—If applicable, explain how loss to follow-up was addressed<br>*Case-control study*—If applicable, explain how matching of cases and controls was addressed<br>*Cross-sectional study*—If applicable, describe analytical methods taking account of sampling strategy	6-7
		(*e*) Describe any sensitivity analyses	NA
**Results**			
Participants	13*	(a) Report numbers of individuals at each stage of study—eg numbers potentially eligible, examined for eligibility, confirmed eligible, included in the study, completing follow-up, and analysed	7
		(b) Give reasons for non-participation at each stage	NA
		(c) Consider use of a flow diagram	NA
Descriptive data	14*	(a) Give characteristics of study participants (eg demographic, clinical, social) and information on exposures and potential confounders	7
		(b) Indicate number of participants with missing data for each variable of interest	7
		(c) *Cohort study*—Summarise follow-up time (eg, average and total amount)	NA
Outcome data	15*	*Cohort study*—Report numbers of outcome events or summary measures over time	*NA*
		Case-control study—Report numbers in each exposure category, or summary measures of exposure	*NA*
		Cross-sectional study—Report numbers of outcome events or summary measures	*7*
Main results	16	(*a*) Give unadjusted estimates and, if applicable, confounder-adjusted estimates and their precision (eg, 95% confidence interval). Make clear which confounders were adjusted for and why they were included	7-9
		(*b*) Report category boundaries when continuous variables were categorized	7-9
		(*c*) If relevant, consider translating estimates of relative risk into absolute risk for a meaningful time period	NA

Continued on next page

For peer review only - http://bmjopen.bmj.com/site/about/guidelines.xhtml

For peer review only

Other analyses	17	Report other analyses done—eg analyses of subgroups and interactions, and sensitivity analyses	NA
**Discussion**			
Key results	18	Summarise key results with reference to study objectives	9
Limitations	19	Discuss limitations of the study, taking into account sources of potential bias or imprecision. Discuss both direction and magnitude of any potential bias	12
Interpretation	20	Give a cautious overall interpretation of results considering objectives, limitations, multiplicity of analyses, results from similar studies, and other relevant evidence	9-13
Generalisability	21	Discuss the generalisability (external validity) of the study results	12-13
**Other information**			
Funding	22	Give the source of funding and the role of the funders for the present study and, if applicable, for the original study on which the present article is based	14

*Give information separately for cases and controls in case-control studies and, if applicable, for exposed and unexposed groups in cohort and cross-sectional studies.

Note: An Explanation and Elaboration article discusses each checklist item and gives methodological background and published examples of transparent reporting. The STROBE checklist is best used in conjunction with this article (freely available on the Web sites of PLoS Medicine at http://www.plosmedicine.org/, Annals of Internal Medicine at http://www.annals.org/, and Epidemiology at http://www.epidem.com/). Information on the STROBE Initiative is available at www.strobe-statement.org.

For peer review only
